# Stable inheritance of *Sinorhizobium meliloti* cell growth polarity requires an FtsN-like protein and an amidase

**DOI:** 10.1038/s41467-020-20739-3

**Published:** 2021-01-22

**Authors:** Elizaveta Krol, Lisa Stuckenschneider, Joana M. Kästle Silva, Peter L. Graumann, Anke Becker

**Affiliations:** 1 Center for Synthetic Microbiology (SYNMIKRO), Philipps-Universität Marburg, Marburg, Germany; 2 Department of Biology, Philipps-Universität Marburg, Marburg, Germany; 3 Department of Chemistry, Philipps-Universität Marburg, Marburg, Germany; 4 Present Address: Max Planck Institute for Terrestrial Microbiology, Marburg, Germany

**Keywords:** Cell growth, Cell polarity, Cellular microbiology

## Abstract

In Rhizobiales bacteria, such as *Sinorhizobium meliloti*, cell elongation takes place only at new cell poles, generated by cell division. Here, we show that the role of the FtsN-like protein RgsS in *S. meliloti* extends beyond cell division. RgsS contains a conserved SPOR domain known to bind amidase-processed peptidoglycan. This part of RgsS and peptidoglycan amidase AmiC are crucial for reliable selection of the new cell pole as cell elongation zone. Absence of these components increases mobility of RgsS molecules, as well as abnormal RgsS accumulation and positioning of the growth zone at the old cell pole in about one third of the cells. These cells with inverted growth polarity are able to complete the cell cycle but show partially impaired chromosome segregation. We propose that amidase-processed peptidoglycan provides a landmark for RgsS to generate cell polarity in unipolarly growing Rhizobiales.

## Abstract (web summary)

In *Sinorhizobium* bacteria, cell elongation takes place only at new cell poles, generated by cell division. Here, Krol et al. show that an FtsN-like protein and a peptidoglycan amidase are crucial for reliable selection of the new cell pole as cell elongation zone.

## Introduction

In all three kingdoms of life, cell polarization is a dynamic phenomenon characterized by active accumulation or confinement of proteins within a part of the cell, resulting in their asymmetric distribution and formation of molecule gradients^1,2^. Binary fission of bacterial cells itself is a source of asymmetry^3^. Polar positioning of a monotrichous flagellum^4,5^, polarized chromosome segregation^6–8^ and unipolar cell wall growth^9^ are striking examples of bacterial cell polarity.

Implicit in bacterial cell growth is an increase in cell volume and surface, involving expansion of the peptidoglycan (PG) sacculus. Insertion of new PG into the existing mesh is mediated by tightly controlled PG hydrolysis, synthesis, and maturation enzymes^10^. Cell elongation of most rod-shaped bacteria takes place in a dispersed manner along the sidewall, using filaments of the actin homolog MreB as scaffold for the PG biosynthesis machinery^11,12^. However, MreB-independent polar cell wall expansion has been reported for a considerable share of bacteria, such as Gram-positive Streptomyces, Mycobacteria and Actinobacteria, and alphaproteobacterial Rhizobiales^9,13–15^. The latter include the plant pathogen *Agrobacterium tumefaciens*, the plant symbiont *Sinorhizobium meliloti* and the animal pathogen *Brucella abortus*, which exhibit unipolar cell wall growth^9,16,17^.

In most of the bacteria, cell division is mediated by a complex multiprotein assembly that includes PG synthesis and remodeling enzymes, designated as divisome^18^. A number of core divisome components are conserved in the majority of bacteria^19^. PG amidases play an important role in septum splitting during cell division in γ-proteobacterial *Escherichia coli*^20^. These enzymes require activation by cognate enzymatically inactive LytM (Lysostaphin-like metalloproteases) domain proteins^21^. PG amidases generate glycan chains free of peptide stems, referred to as denuded PG^22^. The latter serves as binding substrate for the non-essential C-terminal SPOR domain of the essential divisome protein FtsN^22–24^. FtsN was initially identified in *E. coli*, followed by discovery of highly variable FtsN-like proteins in α, β, and δ-proteobacteria^25,26^.

Unraveling the pivotal processes of regulation and scaffolding of unipolar cell wall growth is key to understanding the molecular basis of asymmetric cellular organization that enables coordination of this growth mode with faithful replication and segregation of the genomic DNA and cell division. In *S. meliloti*, polar growth zones are placed at the new cell poles, generated by cell division^9,16^. The chromosomal origin of replication is located at the old cell pole and the newly replicated chromosomal origin migrates to the new, growing cell pole^16^. How stable inheritance of the PG growth zones at the new cell poles is achieved in unipolarly growing Rhizobiales is still enigmatic. A promising candidate for a polar growth scaffold protein is the *A. tumefaciens* growth pole ring protein GPR^27^. Moreover, we recently identified eleven novel essential *S. meliloti* Rhizobial growth and septation (Rgs) proteins with yet-unknown functions, which localized to sites of zonal cell wall synthesis^16,28^. They constitute a protein interaction network, including the FtsN-like cell division protein RgsS, GPR homolog RgsE, and inner membrane components of the Tol-Pal system^28^.

In this study, we show that in *S. meliloti*, polar positioning of the cell wall growth zone correlates with positioning of the FtsN-like protein RgsS. Faithful localization of RgsS to the new cell pole requires its SPOR domain, amidase AmiC and AmiC cofactor LytM domain protein AmcA. Furthermore, our data indicate that the chromosome segregation process is influenced by the polar positioning of RgsS and the cell wall growth zone.

## Results

### *S. meliloti* AmiC generates binding targets for the RgsS SPOR domain at the growth pole and septum

Previously, we observed mVenus-RgsS localization at the sites of zonal PG synthesis at the growth pole and the septum in a *S. meliloti* strain carrying the *mVenus*-*rgsS* gene fusion in place of the *rgsS* wild type allele (Rm2011 *mVenus-rgsS*)^28^. RgsS is a FtsN-like protein, indispensable for cell division^28^. RgsS contains a conserved SPOR domain at the periplasmic C-terminus, designated here as SPOR_RgsS_ (Supplementary Fig. 1). SPOR_RgsS_ shares sequence similarities with SPOR domains of *E. coli* FtsN (FtsN_Ec_), *Pseudomonas aeruginosa* RlpA and *Bacillus subtilis* cell wall amidase CwlC^29–31^, including residues involved in binding of denuded PG in these proteins (Supplementary Fig. 1).

**Fig. 1:**
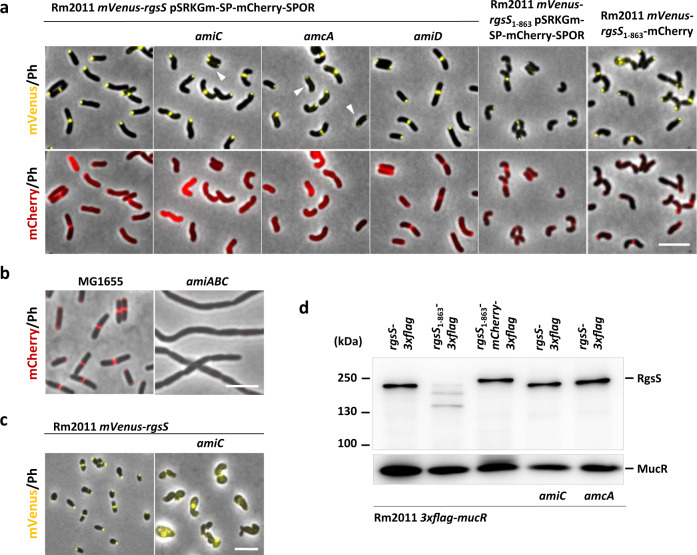
Cell morphology and localization of mVenus-RgsS and mCherry-SPOR_RgsS_ in strains, sufficient or deficient in AmiC, AmcA, or SPOR_RgsS_. **a** Fluorescence microscopy images of exponentially growing TY cultures of indicated *S. meliloti* strains. White arrowheads indicate cells with bipolar mVenus-RgsS localization. Scale bar, 5 µm; Ph, phase contrast. The images are representative of two independent cultivations and microscopy analyses. **b** Fluorescence microscopy images of cells from M9 cultures of indicated *E. coli* strains, carrying pSRKGm-SP-mCherry-SPOR. Scale bar, 5 µm; Ph phase contrast. The images are representative of two independent cultivations and microscopy analyses. **c** Fluorescence microscopy images of cells from exponential phase LB cultures of *S. meliloti* Rm2011 *mVenus-rgsS* and its *amiC*-deficient derivative. Scale bar, 5 µm; Ph phase contrast. The images are representative of three independent cultivations and microscopy analyses. **d** Western blot analysis with α-FLAG antibody of Rm2011 *3xflag-mucR* strain, expressing genes encoding C-terminal 3XFLAG tag-RgsS fusions from the *rgsS* promoter at the native genomic location. Strains were grown in TY supplemented with gentamicin. 3×FLAG-MucR produced from *3xflag-mucR* at the native genomic location was used as a loading control. The result is representative of three biological replicates.

To analyze if SPOR_RgsS_ was able to accumulate at cell division sites, similar to the SPOR domain of FtsN_Ec_^32^, we designed plasmid pSRKGm-SP-mCherry-SPOR for ectopic production of periplasmic mCherry-SPOR_RgsS_. In the Rm2011 *mVenus-rgsS* strain, we observed robust septal mCherry-SPOR_RgsS_ colocalization with mVenus-RgsS and in a major part of the cells also polar colocalization (Fig. 1a and Supplementary Table 1), suggesting that SPOR_RgsS_ binding targets are present at both these sites. mCherry-SPOR_RgsS_ accumulated at the septum in *E. coli* wild-type strain MG1655, but not in a MG1655 mutant strain lacking the PG amidases AmiA, AmiB, and AmiC (Fig. 1b). Thus, SPOR_RgsS_ is likely able to bind denuded PG, generated by amidases in the *E. coli* septum.

Next, we asked if *S. meliloti* SPOR_RgsS_ binding targets were produced by *S. meliloti* PG amidases. As deduced from genome annotation, *S. meliloti* possesses the putative PG amidases AmiC and AmiD, homologous to *E. coli* AmiC and AmiD (Supplementary Figs. 2 and 3), but no AmiA and AmiB homologs. Whereas *E. coli* AmiC is involved in PG splitting during cell division^33^, AmiD is not required for this process^34^. Septal and polar foci of ectopically produced mCherry-SPOR_RgsS_ were still observed in the *amiD* knockout mutant Rm2011 *mVenus-rgsS amiD* but were absent in the *amiC* deletion strain Rm2011 *mVenus-rgsS amiC* (Fig. 1a). These results suggest that *S. meliloti* AmiC but not AmiD activity generates denuded PG that serves as mCherry-SPOR_RgsS_ binding substrate within polar and septal cell wall growth zones.

### AmiC with an intact catalytic site is required for straight rod cell shape and envelope integrity

To further characterize *amiC* and *amiD* mutant strains, we analyzed growth and cell morphology. In TY medium, strains lacking either functional *amiC* or *amiD* were not affected in growth, however the double *amiC amiD* mutation resulted in a minor slow-down of growth (Supplementary Fig. 4). In TY broth, the *amiD* mutant showed wild type-like straight rod cell morphology, whereas the *amiC* mutation resulted in cells with increased curvature (Fig. 1a, Supplementary Figs. 5 and 6, and Supplementary Table 2). Unlike the amidase-deficient *E. coli*^35^, *S. meliloti amiC*-deficient strains showed no cell chaining phenotype. Thus, AmiC is not strictly required for *S. meliloti* cell division.

While TY is a standard medium for *S. meliloti* propagation, we previously observed that cultivation in LB augmented growth and cell morphology defects of strains affected in cell envelope integrity^16,28^. This effect was likely caused by outer membrane destabilization in the absence of divalent cations^36,37^ and was alleviated by addition of CaCl_2_^16,28^. The *amiD* mutant strain grew nearly normally on LB and showed wild-type-like cell morphology in LB broth (Supplementary Figs. 4 and 5). In contrast, the *mVenus-rgsS amiC* strain showed a strong growth defect on LB (Supplementary Fig. 4), and in LB broth produced enlarged cells, which partially lost mVenus-RgsS localization (Fig. 1c, and Supplementary Fig. 5). These defects were alleviated by addition of 2.5 mM CaCl_2_ (Supplementary Figs. 4 and 5). The augmented cell morphology defect of the *amiC* mutant in the absence of CaCl_2_ implies that AmiC has a function in maintaining cell envelope integrity.

To test if the phenotypic alterations observed in the *amiC* mutant resulted from a lack of AmiC enzymatic activity, we generated complementation constructs on the single-copy vector pABC-Psyn. These constructs included the native *amiC* promoter and comprised the native gene or variants encoding AmiC with alanine replacements at the conserved catalytic histidine residues H206 and H276 (Supplementary Fig. 2). The catalytic residues were determined by similarity to *E. coli* AmiC^38^. Western blot analysis using similar constructs, additionally carrying a 3xFLAG tag, showed that H206A and H276A mutations did not affect AmiC protein stability (Supplementary Fig. 7). Complementation with native *amiC*, but not *amiC*_H206A_ or *amiC*_H276A_ restored wild type-like growth, cell morphology and septal localization of mCherry-SPOR_RgsS_ in the *mVenus-rgsS amiC* strain (Supplementary Figs. 4, 5, and 6). This implies that processing of PG by AmiC is required for normal cell growth and morphology as well as septal localization of mCherry-SPOR_RgsS_.

### EnvC-like LytM domain protein AmcA is required for AmiC function

PG amidases in *E. coli*, *Xanthomonas campestris* and *Neisseria gonorrhoeae* require allosteric activation by cognate enzymatically inactive LytM domain proteins^38–41^. In *S. meliloti*, deletion of *SMc03782*, encoding a LytM domain protein, phenocopied the effect of *amiC* deletion. It did not affect growth on TY, abolished septal localization of mCherry-SPOR_RgsS_ and resulted in a curved cell phenotype (Fig. 1a, Supplementary Figs. 4 and 5, and Supplementary Table 2). Moreover, the *SMc03782* mutant grew poorly on LB and showed enlarged cells in liquid LB culture resembling the phenotype of the *amiC* strains (Supplementary Figs. 4 and 5). These defects were relieved upon complementation with an ectopic gene copy driven by the native promoter on single-copy plasmid pABC-Psyn (Supplementary Figs. 4, 5, and 6). Western blot analysis revealed that deletion of *SMc03782* did not affect abundance of 3xFLAG-tagged AmiC (Supplementary Fig. 7), thus *SMc03782* is likely required for AmiC function. Therefore, the gene was named *amcA*, for “amidase C cofactor A”. The LytM domain of AmcA shares similarities with the corresponding regions of *E. coli* PG amidase cofactors EnvC and NlpD^39^ (Supplementary Fig. 8). Like EnvC and NlpD, it lacks the HxH motif, conserved in enzymatically active LytM domains^42^. The remaining AmcA sequence is non-homologous to either EnvC or NlpD, however it contains two predicted coiled-coil-forming alpha-helical regions (Supplementary Fig. 8). This constitutes a similarity to *E. coli* EnvC, which contains two coiled-coil domains^21^. Moreover, the four residues identified as crucial for amidase activation by EnvC^43^ are represented by identical or similar amino acids in AmcA (Supplementary Fig. 8). These findings corroborate the assumption that AmcA is required for AmiC activation.

### SPOR_RgsS_ is non-essential for cell propagation, but is required for straight rod cell morphology

Binding to denuded PG via the SPOR domain was suggested to stabilize septal positioning of FtsN_Ec_^44^. To gain insight into the role of the SPOR domain for RgsS function, we generated the strain Rm2011 *mVenus-rgsS*_1–__863_, producing an mVenus-RgsS variant lacking the SPOR domain. Viability of this strain suggests that the RgsS SPOR domain is non-essential for cell propagation, reminiscent of the FtsN_Ec_ SPOR domain^45^. This truncation of mVenus-RgsS did not result in a growth defect when the *mVenus-rgsS*_1−863_ strain was cultivated on TY agar, whereas growth on LB was substantially reduced (Supplementary Fig. 4). When grown in liquid TY and LB media, *mVenus-rgsS*_1−863_ cells appeared curved, and in LB, a proportion of enlarged cells was observed (Supplementary Fig. 5 and Supplementary Table 2). mCherry-SPOR_RgsS_, produced from pSRKGm-SP-mCherry-SPOR, was detected at the septum in *mVenus-rgsS*_1−863_ cells (Fig. 1a), implying that the truncation of mVenus-RgsS did not affect AmiC activity, which is assumed to generate denuded PG serving as binding substrate for the SPOR domain^22^.

The mVenus fluorescence signal in the *mVenus-rgsS*_1−863_ strain appeared weaker than in the *mVenus-rgsS* wild type and its *amiC*- and *amcA*-deficient derivatives (Fig. 1a and Supplementary Fig. 5). This indicates that RgsS abundance was affected by the lack of the SPOR domain, but not by the *amiC* and *amcA* mutations. We exchanged *rgsS* and *rgsS*_1−863_ with *rgsS-3xflag* and *rgsS*_1−863_*-3xflag*, respectively, at the native genomic location and analyzed protein abundance in the resulting strains by Western blots. In contrast to RgsS-3xFLAG, RgsS_1−863_-3xFLAG was hardly detectable indicating proteolysis (Fig. 1d). A similar result was obtained when 3xFLAG-RgsS and 3xFLAG-RgsS_1−863_ were ectopically produced (Supplementary Fig. 9). The FtsN_Ec_ SPOR domain contains two disulfide bond-forming cysteines that are important for FtsN_Ec_ stability^45,46^. SPOR_RgsS_ contains cysteines at positions 931 and 941. C931A and C941A mutations in the SPOR domain of 3xFLAG-RgsS negatively affected protein abundance similarly to removal of the SPOR domain (Supplementary Fig. 9). This suggests that the intact SPOR domain plays a role in protecting RgsS from degradation, similar to the SPOR domains of FtsN_Ec_ and FtsN-like *C. crescentus* protein CC2007^26,45^.

Since RgsS is an essential protein, we reasoned that its destabilization in the *mVenus-rgsS*_1−863_ strain might have caused physiological alterations, not directly related to the functional link between RgsS and AmiC. Thus, we inserted the mCherry coding sequence into the *rgsS*_1−863_-*3xflag* sequence, generating Rm2011 *rgsS*_1−863_-*mCherry-3xflag*. This modification restored the stability of RgsS lacking the SPOR domain (Fig. 1d). This data shows that although protection of RgsS from degradation required intact SPOR_RgsS_, it is not unique to this domain, since it was also achieved by its replacement with the unrelated mCherry protein.

RgsS_1−863_-mCherry represents a protein, likely unable to bind amidase-processed PG, but retaining stability similar to the full-length RgsS. To gain insight into the function of SPOR_RgsS_, not related to RgsS protein stability, we replaced *mVenus*-*rgsS* with *mVenus-rgsS*_1−863_-*mCherry* at the native genomic location. This modification resulted in cell morphology alterations, similar to the phenotype caused by the *amiC* and *amcA* mutations (Fig. 1e, Supplementary Fig. 5, and Supplementary Table 2). Although *mVenus-rgsS*_1−863_-*mCherry* cells showed a moderate slow-down of growth in LB, no cell swelling was observed (Supplementary Figs. 4 and 5). Thus, maintaining a straight rod cell morphology may require interaction between SPOR_RgsS_ and AmiC-processed PG, whereas AmiC and AmcA likely have additional functions related to maintaining cell envelope integrity.

### AmiC, AmcA, and SPOR_RgsS_ facilitate cell division

**Fig. 2:**
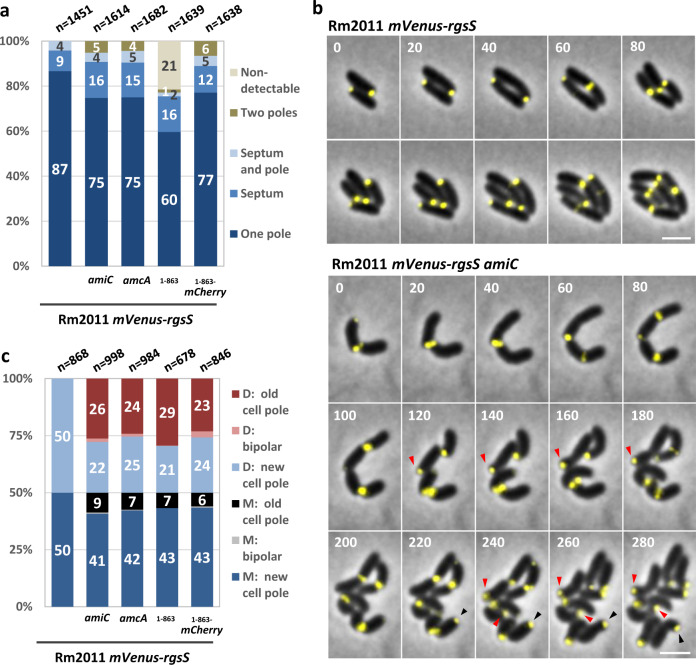
AmiC, AmcA, and the SPOR_RgsS_ are required for stable inheritance of RgsS to the newly formed cell poles after cell division. **a** mVenus-RgsS localization patterns in cells from exponential phase TY cultures. n, total number of analyzed cells. The values represent the mean values of three biological replicates. The standard deviation values and statistical comparison between the strains are shown in Supplementary Table 3. **b** Time lapse microscopy images of Rm2011 *mVenus-rgsS* and its *amiC* mutant derivative, growing on MM-agarose. Merged mVenus fluorescence and phase contrast pictures are shown. Red and black arrowheads point to cells with localization of mVenus-RgsS foci to old cell poles of the daughter and the mother cells, respectively. Time is shown in minutes. Scale bar, 2 µm. The images are representative of three independent cultivations and microscopy analyses. **c** mVenus-RgsS foci inheritance observed in time-lapse microscopy. D daughter cell, M mother cell. n total number of analyzed cells. The values represent the mean values of three biological replicates. The standard deviation values and statistic comparison between the strains are shown in Supplementary Table 5.

In TY cultures of exponentially growing *mVenus-rgsS amiC, mVenus-rgsS amcA, mVenus-rgsS*_1−863_ and *mVenus-rgsS*_1−863_-*mCherry* strains, cells with septal mVenus-RgsS localization were overrepresented compared to the *mVenus-rgsS* wild-type strain, implying a prolonged cell division period (Fig. 2a and Supplementary Table 3). 21% of the *mVenus-rgsS*_1−863_ cells showed no distinct polar fluorescence focus, whereas septal foci were consistently detected. Time-lapse microscopy revealed that in *amiC-* and *amcA-*deficient cells the estimated average duration of the septal mVenus signal increased to 164% and in *mVenus-rgsS*_1−863_ and *mVenus-rgsS*_1−863_-*mCherry* cells to 121% and 118% of that of the *mVenus-rgsS* wild type, respectively (Supplementary Table 4). The mutant strains showed an increase in the estimated average doubling time, consistent with prolonged cell division (Supplementary Table 4). These findings point to an accessory function of AmiC and SPOR_RgsS_ in cell division and corroborate the assumed role of AmcA in AmiC activation.

### AmiC, AmcA, and SPOR_RgsS_ are required for faithful persistence of mVenus-RgsS at the new cell poles after cell division

In the course of the microscopy analysis of *mVenus-rgsS amiC, mVenus-rgsS amcA, mVenus-rgsS*_1−863_, and *mVenus-rgsS*_1−863_-*mCherry* cell populations, exponentially growing in TY medium, we noticed that one to six percent of the cells showed bipolar localization of mVenus-RgsS, which was not observed in *mVenus-rgsS* cells (Figs. 1a and 2a, Supplementary Fig. 5, and Supplementary Table 3). Thus, we analyzed these strains in time-lapse microscopy. Consistent with our previous finding^28^, in the *mVenus-rgsS* strain, mVenus-RgsS persisted at the growing cell pole during the cell elongation phase and in predivisional cells, mVenus-RgsS fluorescence signal accumulated at the septal site and vanished at the cell pole. After septum constriction and cell division, the mVenus-RgsS focus was retained at the new cell pole of each sibling, which became the growing pole (Fig. 2b). Hereafter we define the progeny cell that originated from the compartment that contained the growing pole as the daughter cell and the former compartment that contained the non-growing pole as the mother cell.

In the *amiC*-deficient strain, mVenus-RgsS was located at the growing cell pole during cell elongation and accumulated in the mid-cell area of predivisional cells in a similar manner as in the wild type. However, after cell division, only 63% of *mVenus-rgsS amiC* cells retained the mVenus-RgsS focus at the new cell poles. In 26% of the cells, the mVenus-RgsS fluorescence focus was observed at the former growing pole (old pole of the daughter cells), and in 9% of the cells, the mVenus-RgsS fluorescence focus was located at the former non-growing pole (old pole of the mother cell; Fig. 2b, c and Supplementary Table 5). Moreover, 0.5% of mother cells and 1.5% of daughter cells established a second mVenus-RgsS focus at the old cell pole while retaining a focus at the new cell pole, resulting in bipolar localization. mVenus-RgsS localization at the old cell pole did not detrimentally affect cell growth and division, since these cells were able to elongate and to produce viable progeny. The *amcA-*deficient strain showed similar alterations in mVenus-RgsS focus positioning as the *amiC* mutant (Fig. 2c, Supplementary Fig. 10, and Supplementary Table 5), consistent with the suggested role of AmcA in AmiC activation. Likewise, *mVenus-rgsS*_1−863_ and *mVenus-rgsS*_1−863_*-mCherry* cells showed aberrant mVenus-RgsS focus inheritance (Fig. 2c, Supplementary Figs. 11 and 12, and Supplementary Table 5). Taken together, this data indicates that AmiC-processed PG and SPOR_RgsS_ are crucial for faithful localization of mVenus-RgsS to the new cell poles of both progeny cells.

### Polar positioning of RgsS correlates with polar positioning of other Rgs proteins and PG insertion zones

**Fig. 3:**
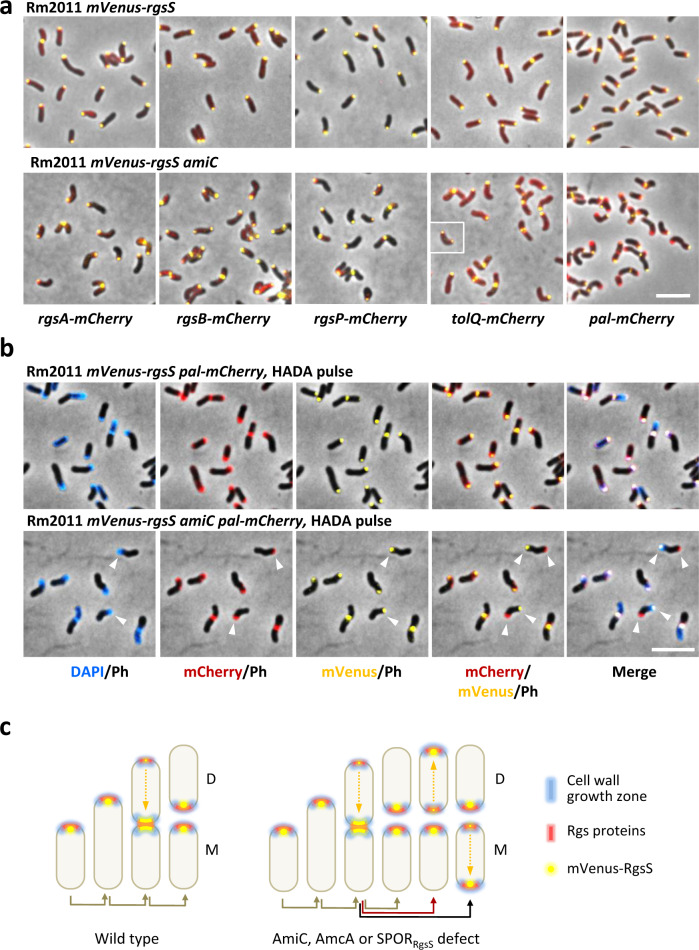
Rgs proteins, TolQ and PG incorporation zones colocalize with RgsS in both wild type and *amiC* cells. **a** Fluorescence microscopy images of exponentially growing Rm2011 *mVenus-rgsS* and Rm2011 *mVenus-rgsS amiC* cells, producing mCherry fusions of the indicated proteins from gene fusions at the native genome locations. Cell samples were taken from exponential phase TY cultures. The insert shows an additional cell of the same strain representative of cells with bipolar colocalization of mVenus-RgsS and TolQ-mCherry fluorescence foci. The images are representative of two independent cultivations and microscopy analyses. Scale bar, 5 µm; Ph phase contrast. **b** Fluorescence microscopy of Rm2011 *mVenus-rgsS* and Rm2011 *mVenus-rgsS amiC* cells, carrying *pal-mCherry* at the native genomic location, pulse-labeled with HADA for 3 min. Cell samples were taken from exponential phase TY cultures. Arrowheads show cells with non-colocalized mVenus-RgsS and Pal-mCherry. Scale bar, 5 µm; Ph phase contrast. The images are representative of three independent cultivations, HADA staining and microscopy analyses. **c** Schematic representation of cell growth polarity inheritance inferred from data shown in this figure, Fig. 2 and Supplementary Figs. 1^0^–12 and 18. In wild-type cells, Rgs proteins and zones of PG biosynthesis are inherited to the new cell pole. In cells, lacking AmiC, AmcA, or the SPOR domain of RgsS, the Rgs proteins and PG synthesis zones are occasionally observed at the old cell pole, representing cells with inverted growth polarity. D daughter cell, M mother cell.

We have previously reported that RgsS, other Rgs proteins and the Tol-Pal system colocalize with RgsP^28^, and that RgsP was exclusively found within the cell wall growth zones at the growth pole and septum^16^. Since we had observed that after cell division, 36% of the *amiC*-deficient cells displayed the mVenus-RgsS focus at the old cell pole (Fig. 2c), we asked if localization of other Rgs and Tol-Pal proteins and polar cell wall growth zones was affected by the *amiC* mutation. Therefore, the corresponding genes were fused to *mCherry* at their native genome locations in both *amiC*-sufficient and -deficient *mVenus-rgsS* strains. In exponentially growing cells of both strains, the polar signals of mCherry-tagged RgsP, RgsA, RgsB, RgsD, RgsE, and TolQ colocalized with mVenus-RgsS (Fig. 3a and Supplementary Fig. 13). Time-lapse microscopy analysis of mCherry-tagged RgsP, RgsA and TolQ confirmed that their localization dynamics correlated with the one of mVenus-RgsS both in *amiC*-sufficient and *amiC-*deficient cells (Supplementary Figs. 14–16). This indicates coordinated conjoint positioning of Rgs proteins and TolQ after cell division, in case of both correct and aberrant polar mVenus-RgsS focus inheritance. Polar Pal-mCherry and mVenus-RgsS foci consistently colocalized only in *amiC*-sufficient cells, whereas in *amiC*-deficient cells, also divergent polar localization of mVenus-RgsS and Pal-mCherry foci was observed (Fig. 3b). Time-lapse microscopy revealed that Pal-mCherry faithfully colocalized with mVenus-RgsS in wild type cells (Supplementary Fig. 17). In *amiC*-deficient cells, which inherited mVenus-RgsS at the old cell pole, Pal-mCherry was retained at the new cell pole and accumulated de novo in the course of cell elongation at the old cell pole (Supplementary Fig. 17). Thus, we considered divergent mVenus-RgsS and Pal-mCherry localization as a marker of cells with mVenus-RgsS focus at the old cell pole. Pulse-labeling with HADA revealed conjoint positioning of PG incorporation zones with mVenus-RgsS in both *amiC*-sufficient and *amiC*-deficient cells, independent of Pal-mCherry focus localization (Fig. 3b, Supplementary Fig. 18, and Supplementary Table 6). Interestingly, a minor fraction of both *amiC*-sufficient and *amiC*-deficient cells exhibited diffuse HADA staining, despite presence of mVenus-RgsS and Pal-mCherry foci in either conjoint or divergent location. These may represent newborn cells that did not start the cell elongation yet. The major proportion of elongating *amiC*-deficient cells contained the single mVenus-RgsS focus, HADA staining zone and Pal-mCherry focus at the same cell pole, indicating cells with normal growth polarity (Supplementary Fig. 18 and Supplementary Table 6). In a minor fraction of the cells, the single mVenus-RgsS focus and a HADA staining zone were found at the cell pole opposite to the one containing the Pal-mCherry focus, indicating cells with inverted growth polarity (Fig. 3c, Supplementary Fig. 18, and Supplementary Table 6). Moreover, bipolar HADA staining was detected in cells with bipolar mVenus-RgsS localization (Supplementary Fig. 18 and Supplementary Table 6). Taken together these data and aberrant polar localization patterns of RgsS in cells lacking functional *amiC* or *amcA* or containing an *rgsS* variant encoding SPOR domain deficient RgsS (Fig. 2a, b, Supplementary Figs. 10–12, and Supplementary Tables 3, 5, and 6), we infer that aberrant positioning of mVenus-RgsS at the old cell pole is promoted by these genetic alterations and correlates with positioning of the PG growth zone, other Rgs proteins and TolQ at the same cell pole (Fig. 3c).

### Inverted cell growth polarity affects positioning of the chromosomal origins of replication

In the time-lapse microscopy experiments, we observed that cells with inverted growth polarity were able to successfully complete the cell cycle and produce viable progeny. Thus, these cells were able to replicate and segregate their genomic DNA. In a newborn wild type *S. meliloti* cell, the chromosomal replication origin (*oriC*, designated here as *oriC*_*1*_) is located at the old, non-growing cell pole and persists there throughout the cell cycle^8,16^. The newly replicated *oriC* (designated here as *oriC*_*2*_) relocates from the old cell pole towards the new, growing cell pole^8,16^. ParB, fused to fluorescent proteins, can be used to visualize the *oriC* due to ParB binding at and around the *oriC* region^47^. We asked how the two *oriC*s were localized in the cells with inverted growth polarity. Therefore, we replaced *parB* with *parB-cerulean* at the native genome location in the *mVenus-rgsS* wild-type strain, in its *amiC* and *amcA* mutant derivatives as well as in the *mVenus-rgsS*_1−863_-*mCherry* strain.

**Fig. 4:**
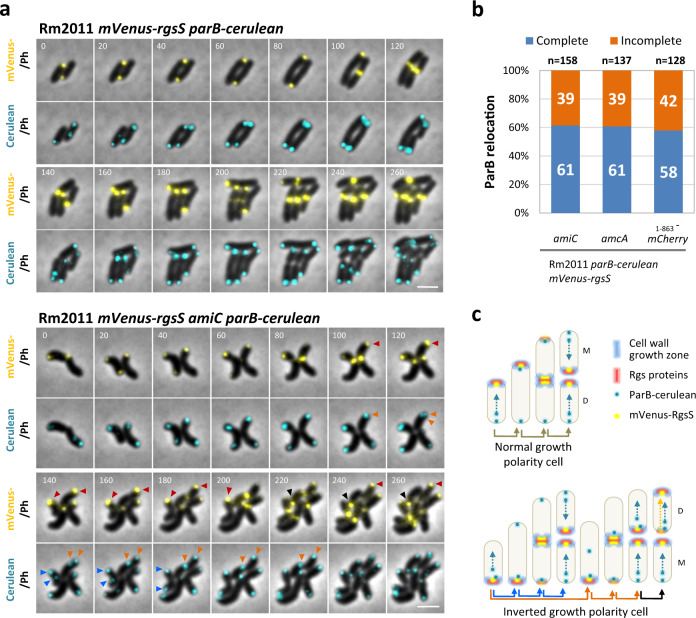
Effects of inverted cell growth polarity on segregation of chromosomal origins. **a** Time-lapse fluorescence microscopy images of Rm2011 *mVenus-rgsS* wild type and its *amiC* mutant derivative, carrying *parB-cerulean* at the native genome location, growing on MM-agarose. Red and black arrowheads indicate accumulation of mVenus-RgsS at the old cell pole of the daughter cell (red) or a mother cell (black). Blue and orange arrowheads show complete (blue) or incomplete (orange) ParB-cerulean focus relocation to the opposite cell pole of a cell with inverted growth polarity. Time is shown in minutes. Scale bar, 2 µm; Ph phase contrast. The images are representative of three independent cultivations and microscopy analyses. **b** ParB-cerulean focus migration towards the new cell pole in cells with mVenus-RgsS focus at the old cell pole. n, total number of analyzed cells. The values represent the mean values of three biological replicates. The standard deviation values and statistic comparison between the strains are shown in Supplementary Table 7. **c** Schematic representation of ParB-cerulean spatiotemporal patterns observed in **a**. In case of cells with normal growth polarity, newborn cells contained a ParB-cerulean focus marking *oriC*_*1*_ at the old cell pole and the migration of a second ParB-cerulean focus marking *oriC*_*2*_ proceeded towards the growing cell pole. In cells with inverted growth polarity, which accumulated the Rgs proteins, which are considered markers for the PG growth zones, at the old cell pole, the migration of the second ParB-cerulean focus marking *oriC*_*2*_ proceeded towards the non-growing cell pole. This resulted in either complete (blue arrows) or incomplete (orange arrows) polar relocation of the second ParB-cerulean focus. A part of the cells that inherited a ParB-cerulean focus at the new cell pole accumulated mVenus-RgsS at the former non-growing pole (black arrow). The color coding is consistent with **a**. D daughter cell, M mother cell.

In *mVenus-rgsS parB-cerulean* cells, time-lapse microscopy revealed the expected spatiotemporal dynamics of ParB-cerulean (Fig. 4a), and all the considered predivisional cells with visible septum constriction contained two polar ParB foci (Fig. 4b). In cells of the *mVenus-rgsS parB-cerulean amiC* strain with mVenus-RgsS focus at the new cell pole (hence with normal cell polarity), wild type-like ParB-cerulean dynamics was observed (Fig. 4a). In the newborn *mVenus-rgsS parB-cerulean amiC* cells with inverted growth polarity, both the ParB-cerulean focus marking *oriC*_*1*_ and mVenus-RgsS were situated at the old, growing cell pole. This resulted in migration of the newly emerged *oriC*_*2*_ towards the non-growing pole. In a major part of these cells, migration of *oriC*_*2*_ was successfully completed (Fig. 4a, b and Supplementary Table 7), whereas in the remaining cells, it stayed partial until the end of the cell cycle (Fig. 4a, b).

After the cell division, *mVenus-rgsS parB-cerulean amiC* cells with incomplete *oriC*_*2*_ relocation towards the non-growing cell pole typically generated a mother cell (former non-growing compartment) with both mVenus-RgsS and ParB-cerulean foci located at the new cell pole (Fig. 4a, c). In a major fraction of such cells, the mVenus-RgsS focus positioned to the old cell pole, which constituted the former non-growing pole (Fig. 4a and Supplementary Table 8). This resulted in a cell with inverted growth polarity, which restored the opposite arrangement of the polar growth zone and *oriC*_*1*_. This implies that not only segregation of *oriC*_*2*_ is affected by the position of the growing cell pole, but also positioning of the elongasome complex may be affected by the localization of *oriC*_*1*_. Time-lapse microscopy of *mVenus-rgsS amcA* and *mVenus-rgsS*_1−863_-*mCherry* strains revealed defects in ParB-cerulean dynamics, similar to the ones of the *mVenus-rgsS amiC* strain (Fig. 4b, Supplementary Figs. 19 and 20, and Supplementary Tables 7 and 8). In rare cases, the septum was formed before *oriC*_*2*_ relocation reached beyond mid-cell, which resulted in formation of one progeny cell containing *oriC*_*1*_ and *oriC*_*2*_, and the other *oriC*-free progeny cell that did not proliferate further (Supplementary Fig. 21). Thus, inverted growth polarity partially interfered with relocation of the daughter chromosome towards the non-growing cell compartment.

### AmiC and SPOR_RgsS_ stabilize the positioning of mVenus-RgsS molecules at the growing cell pole

**Fig. 5:**
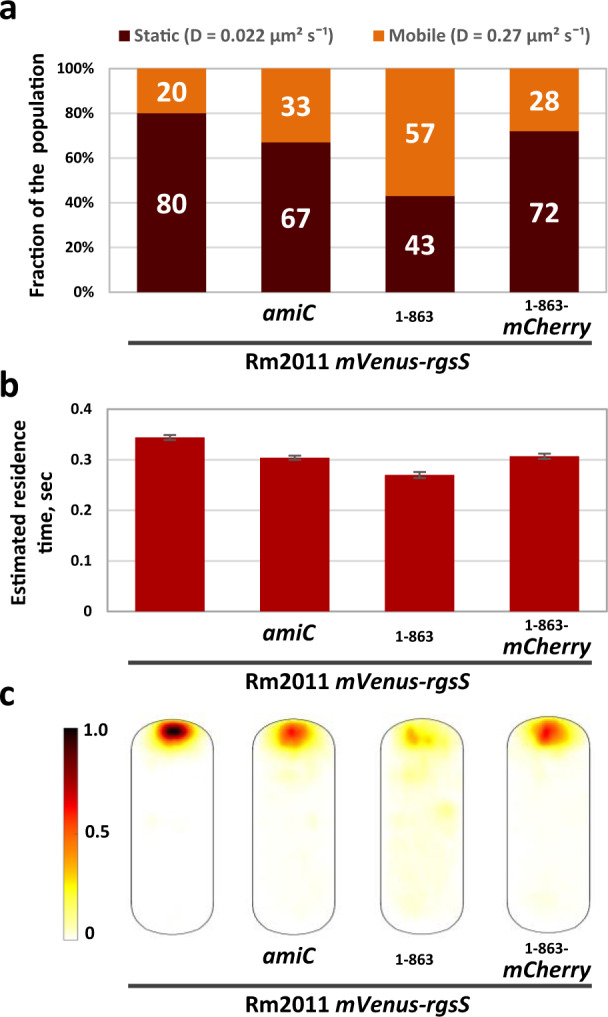
AmiC and SPOR_RgsS_ facilitate stable positioning of mVenus-RgsS molecules at the cell pole. Single-molecule tracking of mVenus-RgsS, mVenus-RgsS_1–__863_, and mVenus- RgsS_1–__863_-mCherry molecules in cells from exponential phase TY cultures. Single molecule tracks were collected from cells, originating from three independent biological replicates. Numbers of single molecule tracks analyzed: Rm2011 *mVenus-rgsS*, *n* = 7556; Rm2011 *mVenus-rgsS amiC*, *n* = 5439; Rm2011 *mVenus-rgsS*_1-863_, *n* = 2296; Rm2011 *mVenus-rgsS*_1-863_*-mCherry*, *n* = 5469. **a** Relative fraction size of molecule populations with two different normalized diffusion constants, determined by Gaussian-mixture model (GMM) fit. Statistics analysis and numerical data are presented in Supplementary Table 9. **b** Estimated average residence times (time a molecule stays in a radius of 128.7 nm for more than nine 20 ms intervals), calculated from single molecule tracking data. Data presented as mean values of residence times calculated for each trajectory. Error bars indicate the standard deviation of the mean. Numerical data is presented in Supplementary Table 9. **c** Probability heatmaps of mVenus-RgsS, mVenus-RgsS_1–__863_, and mVenus-RgsS_1–__863_-mCherry molecules trajectory distribution in a normalized cell. The color code on the left indicates the probability of the molecules to be detected in the given cell area.

Single-molecule tracking (SMT) microscopy was applied to analyze dynamics of mVenus-tagged proteins in *mVenus-rgsS* wild type*, mVenus-rgsS amiC, mVenus-rgsS*_1−863_, and *mVenus-rgsS*_1−863_-*mCherry* cells with one polar mVenus fluorescence focus, hence undergoing cell elongation. Regarding the diffusion coefficient (*D*), the mVenus-RgsS molecules fell into static (*D* = 0.022 µm^2^ s^−1^) and mobile (*D* = 0.27 µm^2^ s^−1^) populations (Fig. 5a, Supplementary Fig. 22, and Supplementary Table 9). An average molecule residence time of 344 ms was estimated from the distribution of dwell events, which could be best fitted with a mixture of two single exponential decay distributions, suggesting two distinct molecule populations with two different residence times (Supplementary Fig. 23). Note that the actual residence times are longer because our analysis involves a convolution of bleaching and mobility of the molecules.

In the *mVenus-rgsS amiC* and *mVenus-rgsS*_1−863_-*mCherry* cells, the size of the static fraction of the tracked molecules as well as their estimated average residence time decreased relative to *mVenus-rgsS* wild type cells (Fig. 5a, b and Supplementary Table 9).

This implies that mVenus-RgsS binding to AmiC-processed PG via its SPOR domain reduces the overall mobility of the protein and therefore stabilizes its position. In *mVenus-rgsS* wild-type cells, the tracked molecules were strongly enriched at the pole, whereas in *mVenus-rgsS amiC* and *mVenus-rgsS*_1−863_-*mCherry* cells, they were more often detected in the remaining cell area (Fig. 5c). In *mVenus-rgsS*_1−863_ cells, the polar enrichment of the protein molecules, the size of the static fraction and the residence time were strongly reduced (Fig. 5a–c). This is consistent with our previous observations of weaker or even non-detectable polar mVenus-RgsS_1−863_ foci (Fig. 1a and Supplementary Figs. 5 and 11). Since removal of the SPOR domain destabilized RgsS (Fig. 1d), it is likely that part of the mVenus-RgsS_1−863_ molecules detected during tracking represented degraded protein.

To further characterize the spatial dynamics of mVenus-RgsS, mVenus-RgsS_1−863_ and mVenus-RgsS_1−863_-mCherry molecules, the tracks were classified regarding their ability to leave the defined confinement area (Supplementary Fig. 24 and Supplementary Table 9). In the *mVenus-rgsS* wild type strain, both confined (29%) and free (68%) tracks were almost exclusively detected at the pole (Supplementary Fig. 24). The large proportion of free tracks concentrated in the narrow polar zone may indicate high on/off rates for binding of mVenus-RgsS to its polar target sites, possibly due to a highly dynamic molecular environment in the polar cell wall elongation zone. In the *mVenus-rgsS amiC* and *mVenus-rgsS*_1−863_-*mCherry* cells, the proportion of free tracks increased to 73% and the proportion of confined tracks decreased to 22% and 23%, respectively, whereas both track types were more abundant outside the polar region (Supplementary Fig. 24). The polar area, containing confined tracks, was extended towards mid-cell (Supplementary Fig. 24). This implies that in the absence of SPOR_RgsS_ or AmiC-processed PG, other factors, which are not spatially restricted to the polar area, could bind RgsS and therefore effectuate confinement of RgsS molecules.

## Discussion

Asymmetric cell growth and division is a widespread phenomenon in prokaryotes and eukaryotes^48^. A prerequisite for asymmetric cell growth in unipolarly growing bacteria, such as *S. meliloti*, is polarization of the elongating cell. We showed that in *S. meliloti*, faithful positioning of the polar growth zone requires the FtsN-like protein RgsS with its SPOR domain as well as peptidoglycan amidase AmiC and its EnvC-like putative cofactor AmcA.

**Fig. 6:**
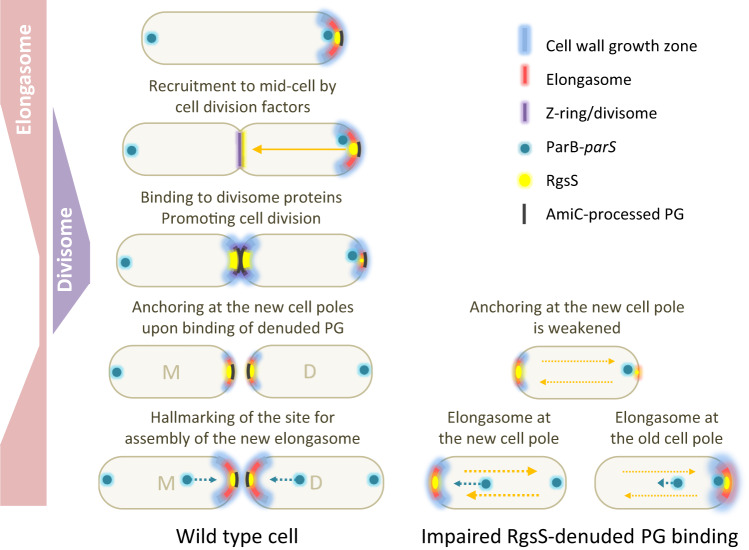
Proposed role of RgsS-denuded PG interaction in faithful positioning of the polar cell elongation complex at the new cell pole. During cell elongation, RgsS is situated at the growing cell pole and its position is suggested to be stabilized by interaction with AmiC-processed PG. During divisome assembly, RgsS is recruited to mid-cell possibly by one or more divisome components. During cell division, AmiC-processed PG is formed and probably provides binding sites for RgsS. This is assumed to ensure stabilization of RgsS localization at the new cell pole. We propose that RgsS hallmarks the site for assembly of the new elongasome. In the absence of AmiC-processed PG-RgsS binding, anchoring of RgsS at the new cell pole is weakened. Thus, RgsS might accumulate and promote formation of the new elongasome at the old cell pole.

We observed that mCherry-SPOR_RgsS_ accumulated at the septum, similarly to the isolated SPOR domain of *C. crescentus* FtsN-like CC2007^26^. This suggests the ability of RgsS to bind denuded PG via its SPOR domain, reminiscent of FtsN_Ec_^32,49^. However, in *S. meliloti*, the impact of this binding extends beyond the cell division process (Fig. 6). We showed that both AmiC and an intact SPOR_RgsS_ are important for confinement of the RgsS molecules in the polar region where the elongasome can be expected to reside. AmiC-processed PG, enriched in this area, could constitute a high-affinity binding target for mVenus-RgsS molecules, whereas lower-affinity binding sites may be provided by RgsS protein interaction partners, present in- and outside the polar area. We propose a hypothetical model of RgsS function, in which during cell elongation, RgsS is localized at the growing cell pole, anchored to denuded PG generated by AmiC. It probably is embedded into the Tol-Pal-Rgs complex and possibly interacts with other elongasome components. In the course of divisome assembly, RgsS is recruited to mid-cell by a yet-unknown potent binding partner and may fulfill a FtsN-like role in cell division. AmiC-processed PG, generated during cell division, could serve to anchor RgsS at the new cell pole where accumulated RgsS is suggested to hallmark the site for assembly of a new elongasome (Fig. 6).

In *E. coli*, prior to cell division, FtsN_Ec_ is recruited to the septum by FtsA in a SPOR domain-independent manner^43^. Likewise, decoupling of the functional link between RgsS and denuded PG in *S. meliloti* did not abolish septal and polar localization of RgsS. However, it resulted in increased mVenus-RgsS mobility and allowed for accumulation of the protein at the old cell pole, accompanied by positioning of the cell wall growth zone (Fig. 6). Thus, positioning of the polar assembly of the elongasome machinery appears to be flexible and hallmarked by RgsS accumulation. Anchoring of RgsS at the new cell pole, dependent on the local AmiC activity, could represent a robust mechanism of continuous propagation of the cell growth polarity, which ensures a uniform opposite arrangement of the old cell pole containing *oriC*_*1*_ and the polar growth zone at the newborn pole in both progeny cells.

The reasons for erroneous RgsS accumulation at the old cell pole are still an open question. *S. meliloti* cell division is asymmetric, with a slightly smaller daughter cell^50^. The onset of *oriC*_2_ migration is delayed in the daughter cell compared to the mother cell in *S. meliloti* as well as in the closely related *A. tumefaciens*^8,51^. After cell division, the mother cell retains its growth polarity, which may provide a head start for the new cell cycle. In contrast, the daughter cell undergoes a growth polarity switch, converting the growing pole into a non-growing pole. It cannot be excluded that this process is not completed at the time point of cell division. In cells lacking AmiC or SPOR_RgsS_, RgsS is not confined at the new cell pole and may be recruited to the remnants of the old elongasome and landmark the old cell pole for elongasome reassembly. This could explain why aberrant growth polarity preferentially arose in the daughter cells.

Our results show that although RgsS is not strictly required for cell elongation^28^, its localization correlates with the localization of polar growth zone proteins and PG synthesis enzymes. Previously, we identified RgsE, a homolog of *A. tumefaciens* pole-forming protein GPR^27^, as RgsS interaction partner^28^. RgsE and GPR are promising candidates for cell elongation scaffold proteins. RgsE is absent from the cell division zone but is detectable at the new cell pole immediately after the cell division. It is tempting to speculate, that RgsE is recruited by RgsS and this initiates the assembly of the new elongasome.

In many bacteria with polarly anchored *oriC*, such as *C. crescentus*, chromosome segregation relies on movement of the new *oriC* towards the new cell pole, enabled by interaction of *oriC*-bound ParB with a ParA gradient^52^. In *C. crescentus*, polar localization of the ParB-ParS complex alone determines the orientation of the ParA gradient, required for *oriC* migration, independent of the pole descendance^53^. Our observation of successful *oriC*_*2*_ migration in a major part of the *S. meliloti* cells with inverted growth polarity suggests that in *S. meliloti* the ParA gradient may be formed independent of the old-new pole arrangement and localization of the cell wall growth zone. However, we also observed delayed and incomplete *oriC*_*2*_ migration towards the non-growing cell pole. In *Streptomyces coelicolor*, the polar protein Scy, implicated in elongation of hyphae, recruits ParA to the hyphal tips and regulates ParA polymerization^54^. Our results imply that in *S. meliloti*, some components of the polar growth complex may influence ParA gradient formation.

PG amidases are known cell separation factors implicated in cell division in bacteria elongating by dispersed cell wall growth. Our results show that in contrast to PG amidases in *E. coli*, *H. pylori*, and *C. crescentus*^20,55,56^, *S. meliloti* AmiC may rather have an accessory function in septum splitting and an additional role in cell wall synthesis. The enzymatic activity of AmiC is probably required for the cell wall integrity-related function and binding targets for mCherry-SPOR_RgsS_ seem to be present at the growth pole. It is therefore likely that PG processing by AmiC takes place during cell elongation. PG synthesis processivity was suggested as an important factor in maintaining the straight rod cell morphology^57^, whereas spatial patterning of PG defects was predicted to define various bacterial cell shapes^58^. Recently, a *Staphylococcus aureus* PG amidase was suggested to constrain PG synthase activity during cell growth^59^. Its absence resulted in spatially dysregulated PG synthesis and strong cell enlargement, reminiscent of *S. meliloti amiC* cells growing in LB^59^. It could be argued that increased mobility of RgsS in the absence of denuded PG might affect the spatiotemporal dynamics of cell elongation zone proteins, leading to a disturbed pattern of PG incorporation. It is likely that in polarly growing *S. meliloti*, PG processing by AmiC is a part of the cell elongation process, required for spatial or functional fine-tuning of the elongasome.

Collectively, our results suggest a pivotal role for the FtsN-like protein RgsS in faithfully positioning of the cell wall growth zone at the new cell pole of *S. meliloti*. Rgs proteins, including RgsS, are conserved in Rhizobiales^28^. These proteins form an interaction network involving components of the Tol-Pal system^28^. We therefore assume that positioning of the growth pole in *S. meliloti* and other unipolarly growing members of the Rhizobiales is determined in a similar manner. The proposed model of growth pole determination involving RgsS and AmiC-processed PG is a striking example how cellular asymmetry can be established in bacteria to enable asymmetric cell elongation.

## Methods

### Bacterial strains and growth conditions

Bacterial strains and plasmids used in this study are shown in the Supplementary Table 10.

*S. meliloti* was grown at 30 °C in TY medium (5 g/L tryptone, 3 g/L yeast extract, 0.4 g CaCl_2_×2H_2_O), LB medium (10 g/L tryptone, 5 g/L yeast extract, 5 g/L NaCl) or LB medium with added 2.5 mM CaCl_2_. For time-lapse microscopy, modified MOPS-buffered minimal medium (MM) (10 g/L MOPS, 10 g/L mannitol, 3.55 g/L sodium glutamate, 0.246 g/L MgSO_4_×7H_2_O, 0.25 mM CaCl_2_, 2 mM K_2_HPO_4_, 10 mg/L FeCl_3_×6H2O, 1 mg/L biotin, 3 mg/L H_3_BO_3_, 2.23 mg/L MnSO_4_×H_2_O, 0.287 mg/L ZnSO_4_×7H_2_O, 0.125 mg/L CuSO_4_×5H_2_O, 0.065 mg/L CoCl_2_×6H_2_O, 0.12 mg/L NaMoO_4_×2H_2_O, pH 7.2) was used. When required, antibiotics were added to agar media at following concentrations: streptomycin, 600 mg/L, kanamycin, 200 mg/L, gentamicin, 30 mg/L, spectinomycin, and 200 mg/L. IPTG was added to 100 µM when using pWBT-NF based constructs.

*E. coli* was grown at 37 °C on LB and antibiotics were added at following concentrations: kanamycin, 50 mg/L, gentamicin, 8 mg/L, spectinomycin, and 100 mg/L. IPTG was added to 100 µM. For liquid cultures, antibiotic concentrations were reduced to the half. For microscopy analysis, *E. coli* strains were grown in M9 medium (3 g/L KH_2_PO_4,_, 12.8 g/L Na_2_HPO_4_ ⋅7H_2_O, 0.5 g/L NaCl, 1.0 g/L NH_4_Cl, 0.05 g/L MgSO_4_) with 0.2% casamino acids and 0.2% maltose. Expression of *mCherry-SPOR*_RgsS_ was induced with 500 µM IPTG for 8 hours.

For western blot analysis, fluorescence microscopy of liquid culture samples and single molecule tracking microscopy, the *S. meliloti* strains were grown in glass tubes with 3 ml medium with shaking at 200 RPM and harvested at OD_600_ between 0.4 and 0.8.

For growth assays on agar plates, the stationary cultures were adjusted to OD_600_ of 2.5, serial dilutions were prepared and 10 µl of each dilution was spotted onto the agar plates. Plates were grown for 48 hours and photographed.

For growth assays in liquid cultures, the precultures were grown in TY medium in glass tubes to stationary phase. In all, 150 µl volume cultures in 96-well microtiter plate were inoculated at OD_600_ = 0.01 and grown with 800 RPM shaking at 30 °C. OD_600_ was recorded every hour in a Biotek Synergy H1 Spectrophotometer. Four independent transconjugant colonies of each strain were used as biological replicates except for the 2011 *mVenus-rgsS amiC amiD* pABC-Psyn strain for which three transconjugant colonies were used.

### Construction of strains and plasmids

Cloning was performed using PCR, restriction digestion, ligation, and *E. coli* transformation. The strains and plasmids generated are listed in the Supplementary Table 5. Primers used in this study are shown in the Supplementary Table 11. The constructs were verified by sequencing.

Plasmid pSRKGm-SP-mCherry-SPOR was constructed by inserting the mCherry coding sequence, N-terminally extended with the RgsB signal peptide encoding sequence (codons 1–25), and the SPOR domain encoding sequence (codons 831–945 of *rgsS*) into pSRKGm under the control of the *lac* promoter.

To generate the strains with markerless *amiC* and *amcA* deletions, the gene flanking regions were cloned into the sucrose selection plasmid pK18mobsacB. The resulting plasmids were introduced into *S. meliloti* by conjugation and subsequently, the double recombinants were selected on LB agar plates containing 2.5 mM CaCl_2_ and 10% sucrose as described previously^60^.

To construct the *amiC* and *amcA* complementation plasmids, the genes including the promoter regions of 368 and 401 bp, respectively, were cloned into shuttle vector pABC-Psyn. To introduce point mutations into the *amiC* sequence, overlap extension PCR was applied. 3xFLAG-tagged versions of native and mutated AmiC complementation constructs were constructed by insertion of *amiC* including the promoter region and missing the stop codon into vector pABC-Psyn-CF.

To generate strain Rm2011 *mVenus-rgsS*_1−863_, the DNA region encoding amino acids 622-863 of RgsS, followed by a stop codon, was cloned into the non-replicative vector pK18mob2 and introduced into Rm2011 *mVenus-rgsS* by conjugation. Homologous recombination resulted in truncation of *mVenus-rgsS* after *rgsS* codon 863.

To generate strain Rm2011 *mVenus-rgsS*_1−863_-*mCherry*, the DNA region, encoding amino acids 622-863 of RgsS was cloned into non-replicative vector pK18mob2-mCherry and introduced into Rm2011 *mVenus-rgsS* by conjugation. Homologous recombination resulted in truncation of *mVenus-rgsS* after *rgsS* codon 863 and its fusion to *mCherry*.

To generate C-terminal fusions of *rgsS* or its derivatives to the 3xFLAG tag-encoding sequence at the native genomic location, the sequence in the range of 500 to 800 bp encoding the C-terminal portion of RgsS was cloned in frame into the non-replicative vector pG18mob-CF and resulting plasmids were introduced into *S. meliloti* by conjugation. Homologous recombination resulted in replacement of the native gene with a tagged gene copy. To generate the ectopic N-terminal fusions of native or mutated RgsS to the 3XFLAG-tag, corresponding coding sequences were inserted into plasmid pWBT-NF under the control of *lac* and T5 promoters.

To generate the strains carrying C-terminal mCherry fusions to *rgsA*, *rgsB*, *rgsD*, *rgsE*, *rgsP*, and *pal* at the native genomic location, the corresponding non-replicative constructs based on pK18mob-mCherry were introduced into *S. meliloti* by conjugation. Homologous recombination resulted in replacement of the native gene with the *mCherry*-fused version. To construct the strain carrying a 3′ *mCherry* fusion to *tolQ* at the native genomic location, the corresponding non-replicative construct was introduced by conjugation. Homologous recombination resulted in insertion of the *tolQ*-*mCherry* fusion, including the native promoter, upstream of the *tolQRAB* operon promoter region.

To obtain the *E. coli amiABC* triple deletion mutant, the *amiA* deletion marked with a kanamycin resistance cassette in *E. coli* strain JW2428, was introduced into the *E. coli* MG1655 strain using P1 transduction^61^. The kanamycin resistance cassette was removed using transformation with plasmid pCP20^62^. Subsequently, the *amiC* deletion, marked by the kanamycin resistance cassette in strain JW4127 was introduced using P1 transduction. The resulting double *amiA amiC* deletion mutant was cured of the kanamycin resistance marker using transformation with plasmid pCP20 and simultaneously, the *amiA amiC* strain was co-transformed with plasmid pSRKGm-SP-mCherry-SPOR. Finally, the *amiB* deletion, marked by the kanamycin resistance cassette in strain JW5449, was introduced into the *amiA amiC* strain carrying pSRKGm-SP-mCherry SPOR using P1 transduction.

### Fluorescence microscopy

Microscopy was performed using the Nikon microscope Eclipse Ti-E equipped with a differential interference contrast (DIC) CFI Apochromat TIRF oil objective (100x; numerical aperture of 1.49) and a phase-contrast Plan Apo l oil objective (100x; numerical aperture, 1.45) with the AHF HC filter sets F36-513 DAPI (excitation band pass [ex bp] 387/11 nm, beam splitter [bs] 409 nm, and emission [em] bp 447/60 nm), F36-504 mCherry (ex bp 562/40 nm, bs 593 nm, and em bp 624/40 nm), F36-525 EGFP (ex bp 472/30 nm, bs 495 nm, and em bp 520/35 nm) and F36-528 YFP (ex bp 500/24 nm, bs 520 nm, and em bp 542/27 nm). Images were acquired with an Andor iXon3 885 electron-multiplying charge-coupled device (EMCCD) camera.

For microscopy of exponentially growing cultures, 2 µl of TY cultures at OD_600_ of 0.4–0.8 were spotted onto 1% Molecular biology grade agarose (Eurogentec) pads, let dry for 2–3 min, closed with cover glass and microscoped. For time-lapse microscopy, bacteria from exponential growth phase TY cultures were diluted 1:20 and 2 µl were spread by gravity flow on the MM agarose pad and let dry for 14 min. The pads were closed air-tight with a cover slip and images were acquired every 20 min in an incubation chamber at 30 °C.

Staining of *S*. *meliloti* cells with fluorescently-labeled D-amino acid HADA was performed as follows: 1 μl of 36 mM HADA dissolved in dimethylsulphoxid was added to 80 μl of the exponentially growing TY culture. The culture was grown further for 3 min at 30 °C with shaking at 800 rpm. Cells were fixed with 186 μl of 100% ethanol for 10 min at room temperature, washed three times with 0.9% NaCl and microscoped.

Quantification of fluorescent protein localization patterns in snapshots and time-lapse images was performed manually using NIS software (Nikon). To evaluate the presence and localization of fluorescence foci within cells grown in liquid cultures, 5–15 images of the same sample were visually evaluated and cells with particular fluorescence foci arrangements were counted. In time-lapse microscopy analysis, tiles of images containing fluorescence signals merged with phase contrast were generated and visually evaluated for fluorescence foci dynamics, foci persistence in septum and cell cycle duration. Cell morphology analysis was performed using the MicrobeJ 5.13 l plugin to the ImageJ software (detection settings for cell length: 1–6 µm; for cell width: 0.5–1 µm, for cell curvature: 0-max).

### Single molecule tracking microscopy and data analysis

The single molecule tracking (SMT) data were obtained with a customized “slim field” microscope (Nikon Eclipse Ti microscope; 100x oil-immersion objective, NA = 1.49) with a 514-nm laser diode beam line of 100 mW maximal power (~250 W/m^2^ were usually employed). The fluorophores were bleached to single molecule level by the laser (representative data is shown in Movie S1 and Supplementary Fig. 25) to be able to follow the single molecules that can be identified as single bleaching events. All the movies were acquired with 20 ms streams, 3000 frames) and acquired by an EMCCD camera (ImageEM X2 EM-CCD, Hamamatsu).

Movies were cropped to only include the frames containing single molecules using Fiji software^63^. To obtain trajectories of single molecule movements, the cropped movies were analyzed by Utrack 2.2.1^64^. The gathered trajectories were evaluated by SMTracker^65^ (https://sourceforge.net/projects/singlemoleculetracker/). The Gaussian Mixture Model (GMM) was used to determine normalized diffusion coefficients and fraction sizes of molecules fitting into either mobile or static populations. For visualization of molecules and their tracks, these were projected into a normalized cell. Molecules were classified as confined if they did not leave the confinement radius of 120 nm over a period of 180 ms or longer. Molecules were classified as free if they left the confinement radius of 120 nm at each of the 20 ms steps over a period of 180 ms or longer. The confinement radius corresponds to three times the localization error.

### Western blot

*S*. *meliloti* strains were grown in TY, supplemented with corresponding antibiotics and IPTG when indicated, to OD_600_ of 0.4–0.8. Cells were collected by centrifugation, resuspended in protein loading dye (50 mM Tris-HCl pH 6.8, 2% SDS, 10% glycerol, 12.5 mM EDTA, 0.02% bromophenol blue) to an OD_600_ of 10, frozen in liquid nitrogen and stored at −20 °C until use. Cells were lysed for 10 min at 95 °C and 10 μl of cell lysates were loaded to SDS-PAGE gel (separating gel: 0.375 M Tris-HCl pH 8.8 0.1% SDS, 8% acrylamide:bisacrylamide 37.5:1 (Fig. 1d), 12% acrylamide:bisacrylamide 37.5:1 (Supplementary Fig. 7) or 6% acrylamide:bisacrylamide 37.5:1 (Supplementary Fig. 9), ammonium persulfate 0.1% Temed, 0.001%; stacking gel: 0.125 M Tris-HCl pH 6.8 0.1% SDS, 6% acrylamide:bisacrylamide 37.5:1 (Fig. 1d and Supplementary Fig. 7) or 4% acrylamide:bisacrylamide 37.5:1 (Supplementary Fig. 9), ammonium persulfate 0.1% Temed, 0.001%) that was run in the running buffer (Tris base 3.03 g/L, Glycine 14,4 g/L, SDS 1 g/L) and separated proteins were transferred to a PVDF membrane (Thermo Fisher Scientific) using a semidry blotting procedure in transfer buffer (0.025 M Tris base, 0.192 M glycine, 20% methanol). The membranes were blocked for one hour at room temperature in PBSTM (1.44 g/L Na_2_HPO_4_*2H_2_O, 0.24 g/L KH_2_PO_4_, 0.2 g/L KCl, 8 g/L NaCl, 1 ml/L TWEEN-20, 2% milk powder, pH 7.2), washed one time with PBST (1.44 g/L Na_2_HPO_4_*2H_2_O, 0.24 g/L KH_2_PO_4_, 0.2 g/L KCl, 8 g/L NaCl, 1 ml/L TWEEN-20, pH 7.2) and hybridized overnight at 4 °C in 15 ml PBST supplemented with Monoclonal ANTI-FLAG® M2-Peroxidase (HRP) antibody produced in mouse (Sigma-Aldrich; 1:1000 dilution) in 50 ml falcon tubes on a rolling shaker. Membranes were washed 4 times for 10–15 min with PBST at 4 °C, developed with Pierce ECL Western Blotting Substrate (Thermo Fisher Scientific) according to manufacturer instructions and imaged using the luminescence image analyzer LAS-4000 (Fujifilm).

### Protein sequence analysis

Protein sequence analyses were conducted using the online tools BLASTP (https://blast.ncbi.nlm.nih.gov/Blast.cgi), Phobius^66^ (https://phobius.sbc.su.se/), RaptorX^67^ (http://raptorx.uchicago.edu/), SWISS-MODEL^68^ (https://swissmodel.expasy.org/), and Coiled-coils^69^ (https://npsa-prabi.ibcp.fr/cgi-bin/npsa_automat.pl?page=/NPSA/npsa_lupas.html).

### Reporting summary

Further information on research design is available in the Nature Research Reporting Summary linked to this article.

## Supplementary information

Supplementary Information

Description of Additional Supplementary Files

Supplementary Movie 1

Reporting Summary

## Data Availability

The authors declare that the main data supporting the findings of this study are available within the article and its Supplementary Information files. All other data are available from the corresponding author upon reasonable request. Source data are provided with this paper.

## Source data

Source data
