# Supplementary material for: Stable inheritance of Sinorhizobium meliloti cell growth polarity requires an FtsN-like protein and an amidase
Source: Nat Commun. 2021 Jan 22;12:545. doi: 10.1038/s41467-020-20739-3 (PMC7822825; doi:10.1038/s41467-020-20739-3)
Supplement: Supplementary file 1 — Supplementary Information [file 41467_2020_20739_MOESM1_ESM.pdf]

Supplementary material

Stable inheritance of *Sinorhizobium meliloti* cell growth polarity requires an FtsN-like protein and an amidase

Elizaveta Krol, Lisa Stuckenschneider, Joana M. Kästle Silva, Peter L. Graumann, Anke Becker

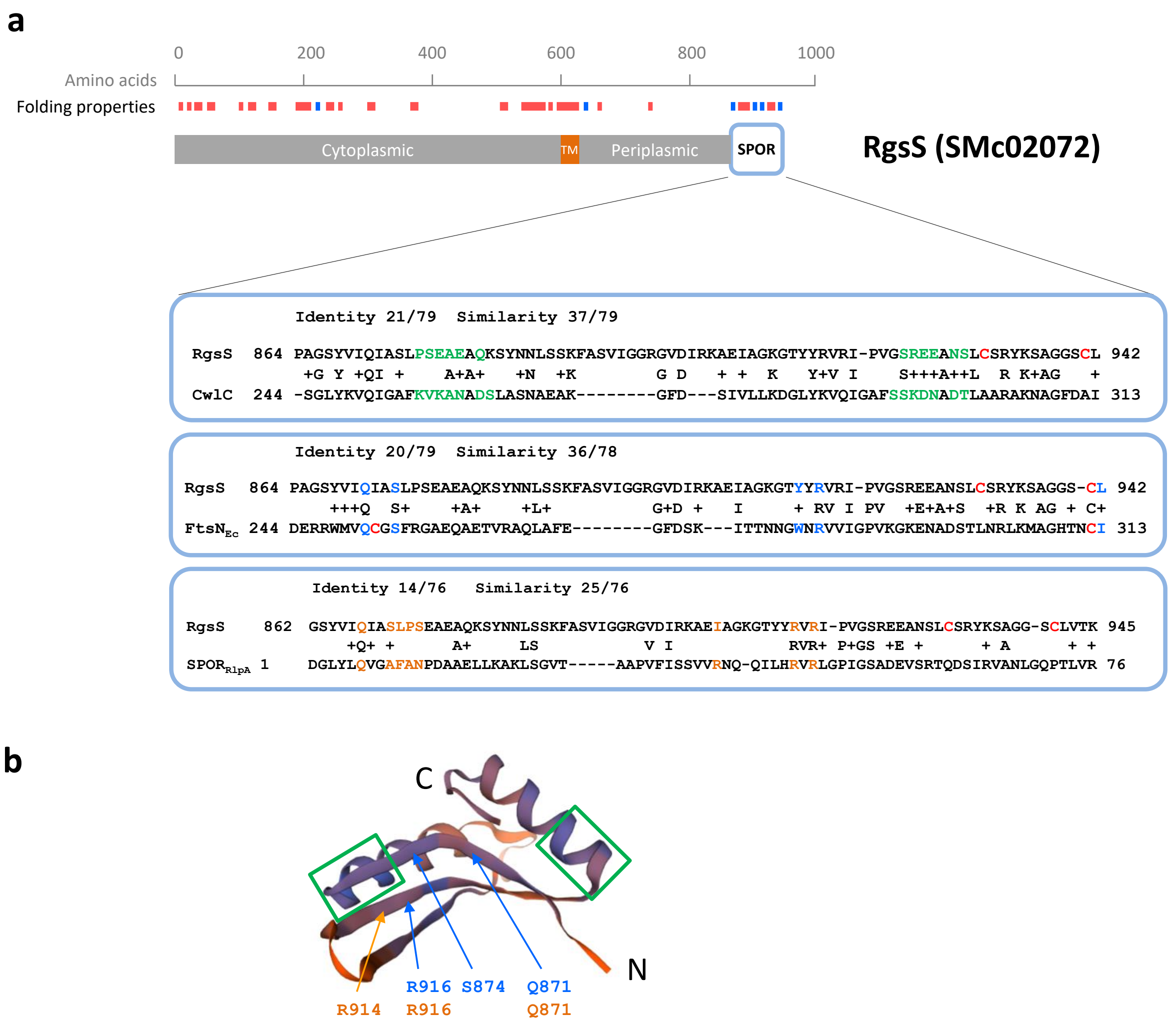

Supplementary Figure 1. Main protein sequence features of *S. meliloti* RgsS. A. Transmembrane segment (TM) was identified using Phobius online tool[1]. Predicted folding properties were determined using RaptorX online tool[2] and are drawn approximately to scale. Red bars indicate alpha-helices, and blue bars indicate β-strands. Conserved SPOR (pfam05036, Sporulation related domain) domain was determined using BLASTP. Similarity to *Bacillus subtilis* ClwC and *E. coli* FtsN and *P. aeruginosa* RlpA was determined using SWISS-MODEL online tool[3], as a result of structure modelling upon either structure template. Cysteine residues are shown in red. Green highlights CwlC residues, identified within PG binding regions[4], and corresponding RgsS residues. Blue highlights FtsN residues, identified as necessary for PG binding[5], and corresponding RgsS residues. Orange highlights RlpA residues, identified as involved in PG binding[6], and corresponding RgsS residues. **B.** Structure model of RgsS SPOR domain generated using SWISS-MODEL online tool with *B. subtilis* CwlC as a template. The regions suggested to be involved in the contacts with denuded PG in CwlC are shown in green boxes. Arrows point to predicted location of the residues identical to the FtsN or Rlp residues involved in binding of denuded PG.

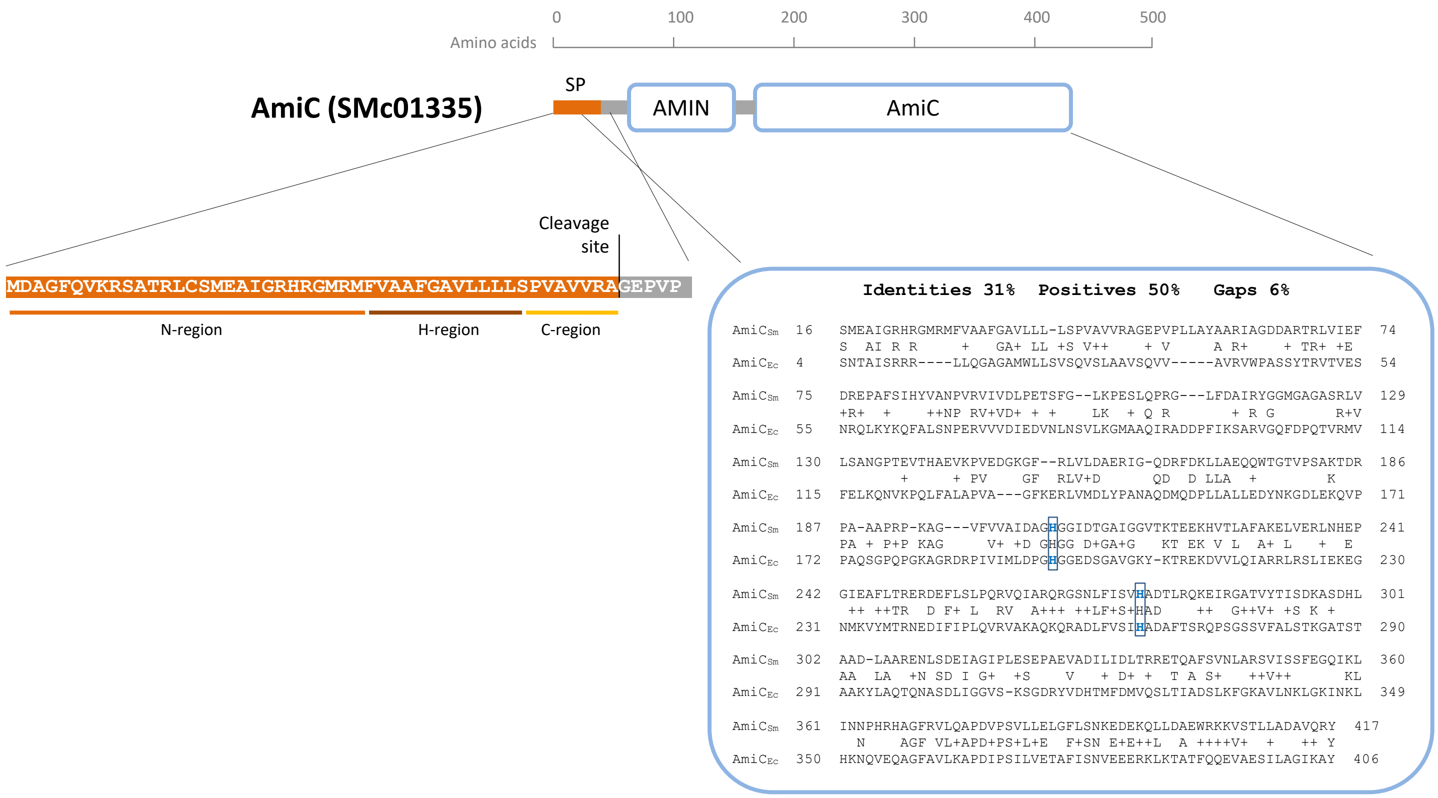

Supplementary Figure 2. Main protein sequence features of *S. meliloti* AmiC. Signal peptide sequence (SP) and its subregions as identified using the Phobius online tool. Conserved AMIN (pfam11741) and AmiC (COG0860, N-acetylmuramoyl-L-alanine amidase) domains were determined using BLASTP. Homology to *E. coli* AmiC (AGX34823.1) is shown. Histidine residues important for *E. coli* AmiC enzymatic activity and corresponding residues in *S. meliloti* AmiC are shown in blue font and boxed.

AmiD (SMc01854)

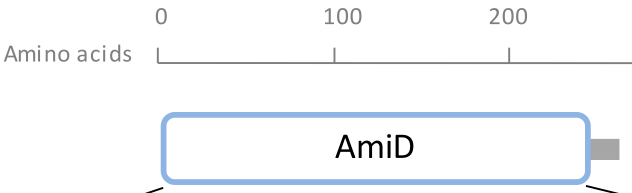

Identities 38%   Positives 50%   Gaps 7%

```
AmiDSm  22   RAGGRRPDMIILHYTGMETAASALDWLCREESQVSCHYFV-------DEEGRIAQLVPEE   74
             +A   R   ++++HYT  +    S+L   L    + QVS  HY  V        + + RI  QLVPE+
AmiDEc  38   QAAYPRIKVLVIHYTA-DDFDSSLATLT--DKQVSSHYLVPAVPPRYNGKPRIWQLVPEQ   94

AmiDSm  75   RRAWHAGKGVWKGETDINSCSIGIEIANAGHPGG-----LPDFPEAQIEAVAELCLDCGE   129
               AWHAG   W+G T +N   SIGIE+ N G                F  AQI+A+   L  D
AmiDEc  95   ELAWHAGISAWRGATRLNDTSIGIELENRGWQKSAGVKYFAPFEPAQIQALIPLAKDIIA   154

AmiDSm  130  RWQITPERVLAHSDIAPIRKVDPGENFPWDVLFRRGVGHWVEPAPVRGGRFFQRGDSGQP   189
             R+  I  PE  V+AH+DIAP  RK DPG   FPW   L ++G+G W   P   R   +        P
AmiDEc  155  RYHIKPENVVAHADIAPQRKDDPGPLFPWQQLAQQGIGAW--PDAQRVNFYLAGRAPHTP   212

AmiDSm  190  VEALQSMLLLYGYGVEITGDFCARTE-GVVAAFQRHFRQSRVDGIADVSTIDTLHRLL   246
             V+     + LL   YG ++   D    R +  V+ AFQ HFR +   +G AD   T       LL
AmiDEc  213  VDTASLLELLARYGYDVKPDMTPREQRRVIMAFQMHFRPTLYNGEADAETQAIAEALL   270
```

Supplementary Figure 3. Main protein sequence features of *S. meliloti* AmiD. No signal peptide sequence or transmembrane domains were identified using the Phobius online tool. Homologies to *E. coli* AmiD (AGX32997.1) and the conserved AmiD (COG3023, N-acetyl-anhydromuramyl-L-alanine amidase AmpD) domain were determined using BLASTP.

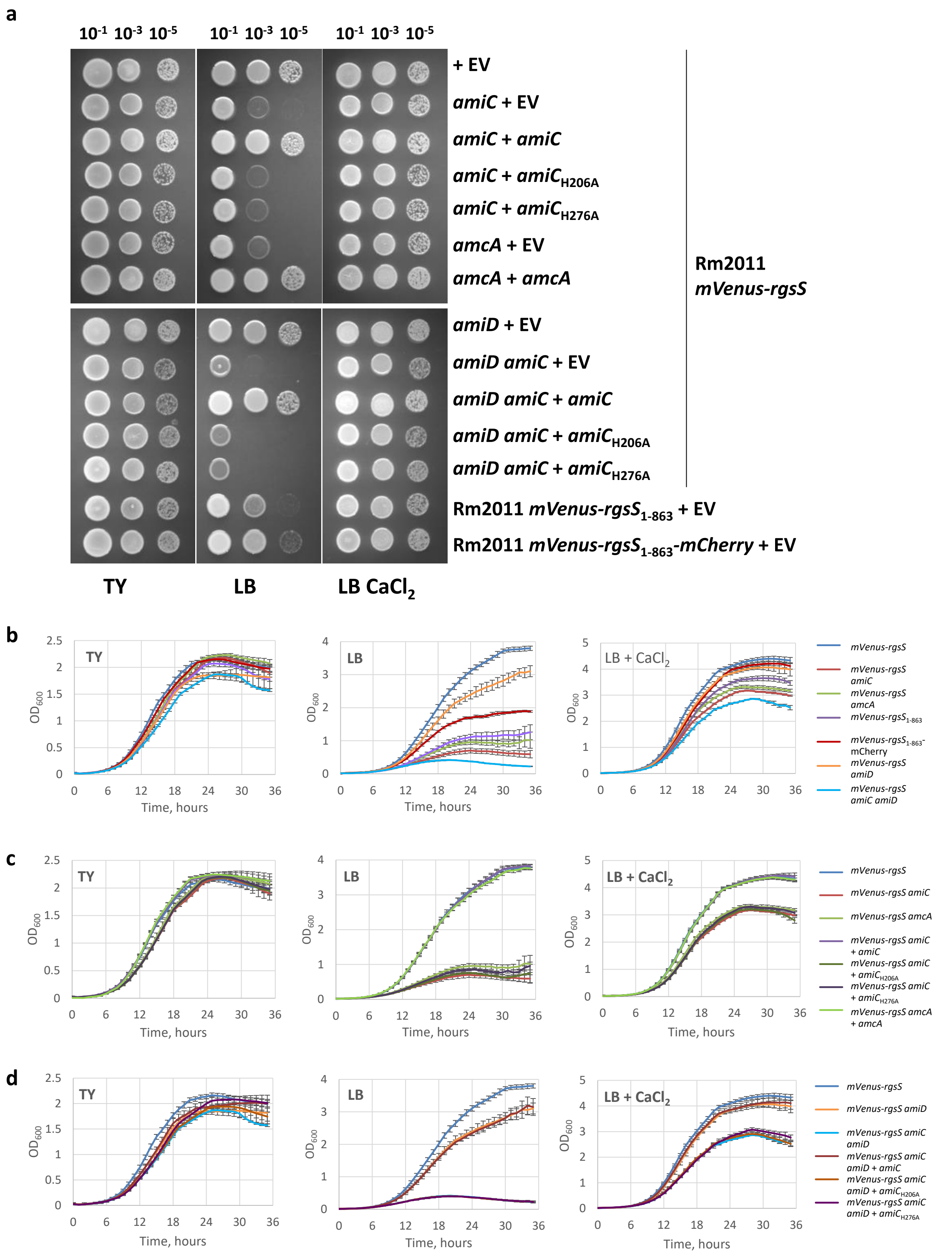

Supplementary Figure 4. Effects of *amiD*, *amiC, amcA* or SPOR$_{RgsS}$ defects on *S. meliloti* growth on TY, LB or LB with added $CaCl_2$. Growth of Rm2011 *mVenus-rgsS* and its mutant derivatives, carrying either the empty single-copy vector pABC-Psyn (+ EV) or the respective complementation construct. **A.** Growth on agar plates. Stationary cultures were adjusted to OD_{600} of 2.5 and 10 µl of the indicated serial dilution were spotted. Plates were imaged after 48 hours of growth. **B-D.** Growth in liquid media. 100 µl media were inoculated with corresponding strains at OD_{600} of 0.01 in four replicates (three replicates for Rm2011 *mVenus-rgsS amiC amiD* + EV), representing independent transconjugant colonies, and the growth was recorded for 35 hours. Data are presented as mean OD_{600} values. Error bars indicate the standard deviation of the mean OD_{600} values. **B.** Growth of indicated strains, carrying empty vector pABC-Psyn. **C.** Growth of *amiC* and *amcA* mutant strains, carrying either empty vector pABC-Psyn or the indicated complementation construct. **D**. Growth of *amiD* and *amiD amiC* mutant strains, carrying either empty vector pABC-Psyn or the indicated complementation construct.

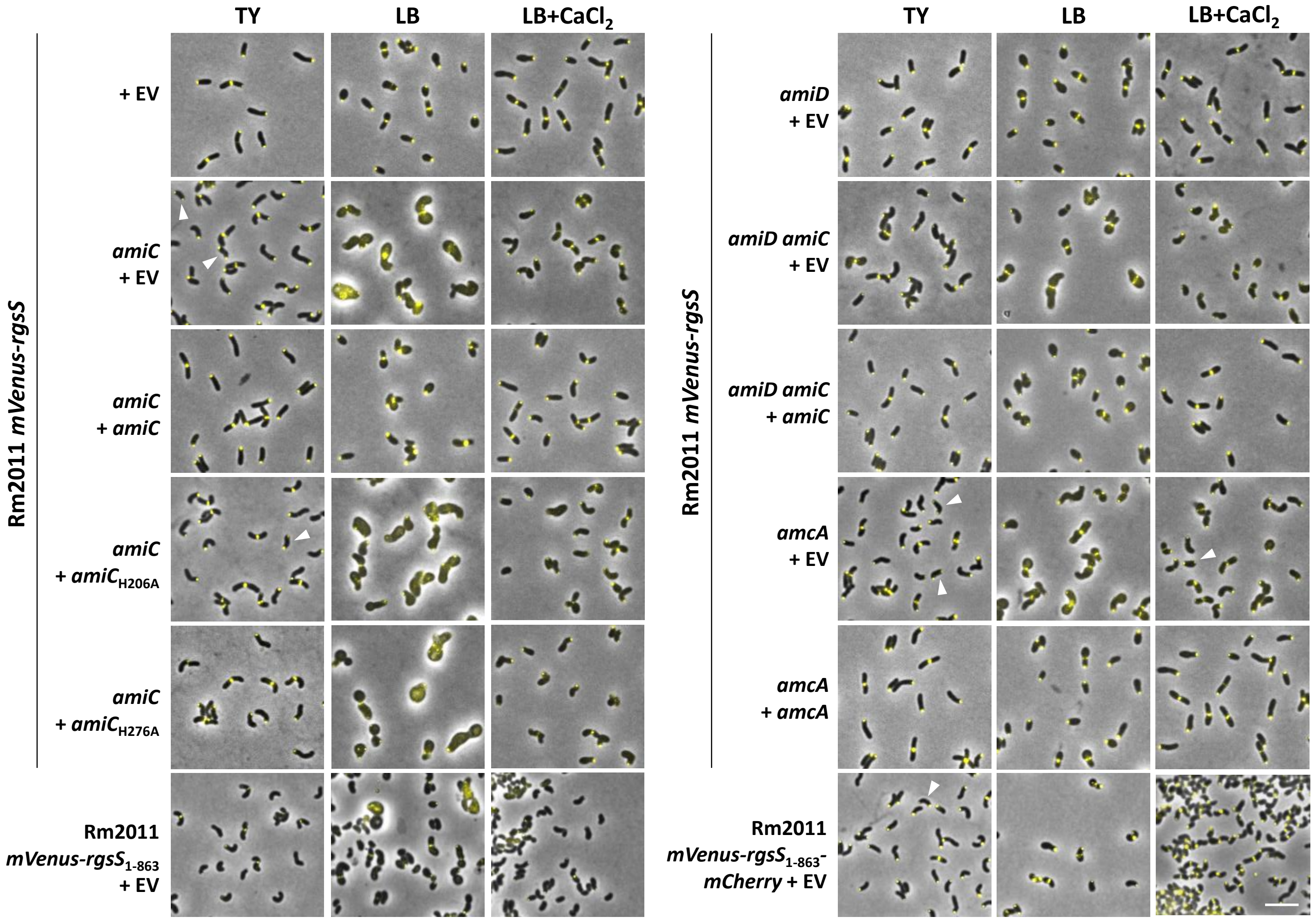

Supplementary Figure 5. Effect of *amiC* and *amcA* deletions and removal of the $SPOR_{RgsS}$-encoding part of *rgsS* on cell morphology in TY, LB and LB with added $CaCl_2$. Merged phase contrast and mVenus fluorescence microscopy images of Rm2011 *mVenus-rgsS* and its mutant derivatives, carrying either the empty single-copy vector pABC-Psyn (+ EV) or the respective complementation construct. White arrowheads point to cells with bipolar mVenus-RgsS signal. Strains were grown in TY, LB or LB with added 2.5 mM $CaCl_2$ to exponential growth phase. Scale bar, 5 µm. The images are representative of two independent cultivations and microscopy analyses.

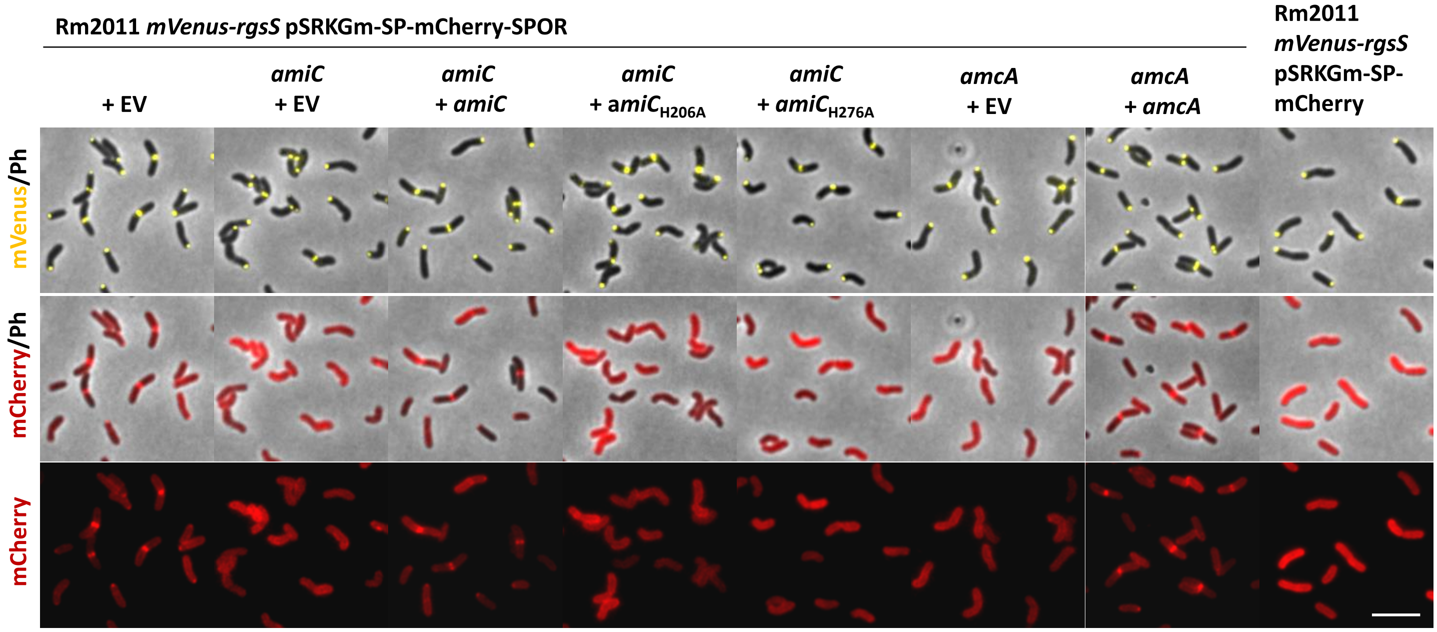

Supplementary Figure 6. AmiC activity, required for promoting mCherry-SPOR$_{\mathrm{RgsS}}$ localization, is abolished by mutations of conserved catalytic histidine residues H206 and H276. Fluorescence microscopy images of Rm2011 *mVenus-rgsS* and its *amiC* mutant derivative, carrying pSRKGm-SP-mCherry-SPOR and either the empty single-copy vector pABC-Psyn (+ EV) or the respective complementation construct. Samples were taken from exponential phase TY cultures. Scale bar, 5 µm; Ph, phase contrast. The images are representative of two independent cultivations and microscopy analyses.

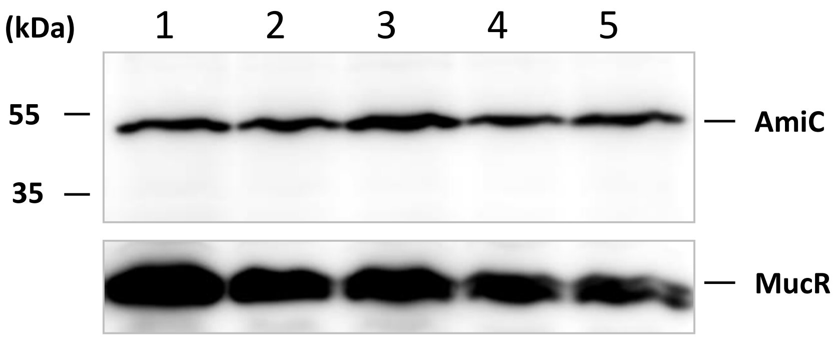

1 Rm2011 *3xflag-mucR* pABC-amiC-3xflag
2 Rm2011 *3xflag-mucR amiC* pABC-amiC-3xflag
3 Rm2011 *3xflag-mucR amcA* pABC-amiC-3xflag
4 Rm2011 *3xflag-mucR amiC* pABC-amiC$_{H206A}$-3xflag
5 Rm2011 *3xflag-mucR amiC* pABC-amiC$_{H276A}$-3xflag

Supplementary Figure 7. AmiC protein abundance is not influenced by mutations in the conserved active site histidine residues or by deletion of *amcA*. Western blot analysis of AmiC-3×FLAG using α-FLAG antibody. Native and mutated AmiC variants were produced ectopically from the native promoter on the single-copy plasmid pABC-Psyn in Rm2011 *flag-mucR* and its *amiC* or *amcA* derivatives, exponentially growing in TY supplemented with spectinomycin. 3×FLAG-MucR produced from *3xflag-mucR* at the native genomic location was used as a loading control. The result is representative of three biological replicates.

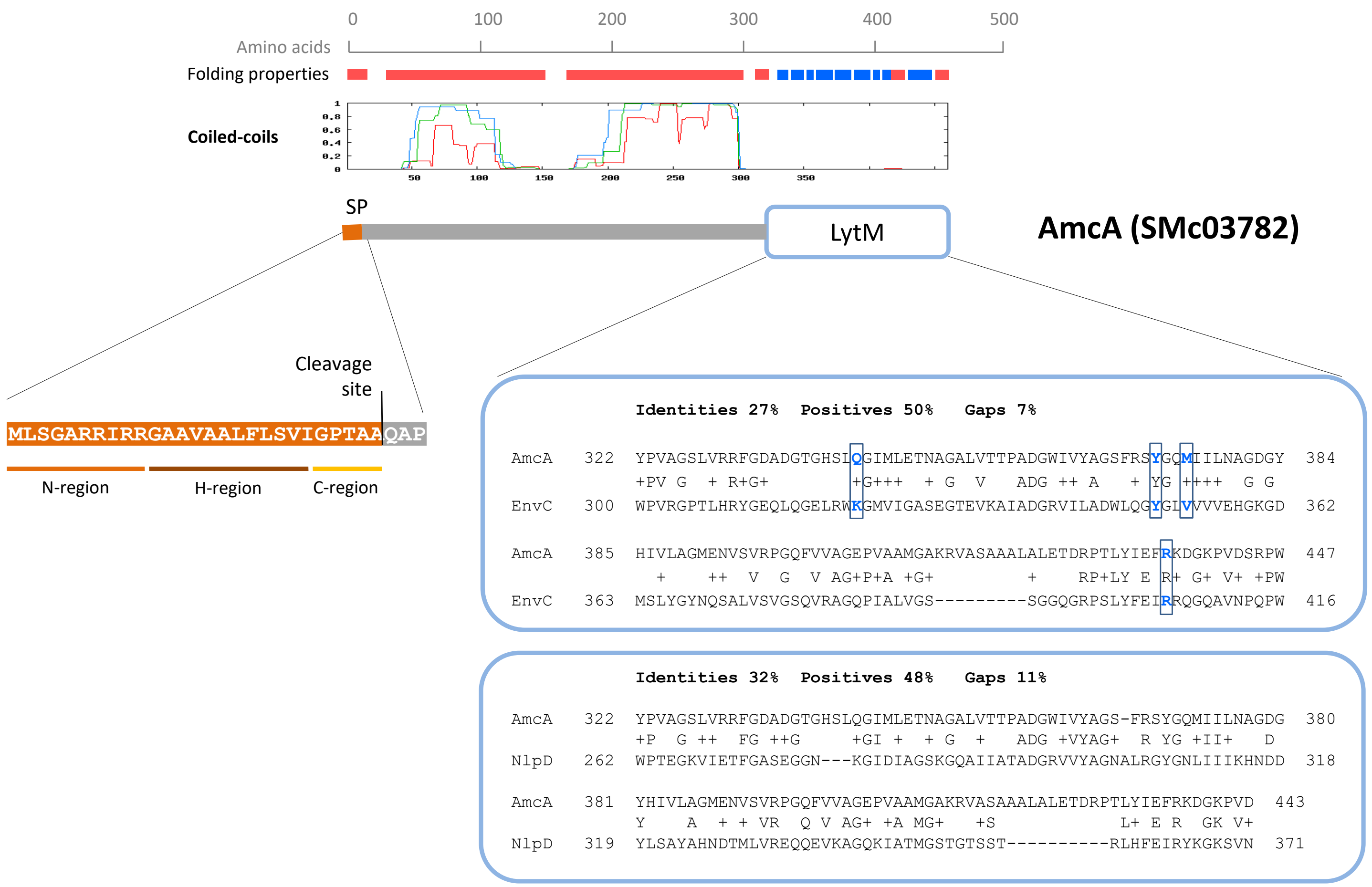

Supplementary Figure 8. Main protein sequence features of AmcA. Signal peptide sequence (SP) and its subregions as identified using the Phobius online tool. Predicted folding properties were determined using the RaptorX online tool and are drawn approximately to scale. Red bars indicate alpha-helices, and blue bars indicate β-strands. Probability of coiled-coil formation was determined by the Coiled-coils online tool[7]. The X-axis shows the amino acid position and the Y-axis shows the calculated probability of coiled-coil formation. Blue, green and red correspond to a 28, 21 or 14 amino acids search window. Homology of the conserved LytM domain (pfam01551, Peptidase family M23) to *E. coli* EnvC (AGX35565) and NlpD (AGX34751) was determined using BLASTP. Blue highlights EnvC residues identified as necessary for AmiB activation by EnvC[8] and corresponding AmcA residues.

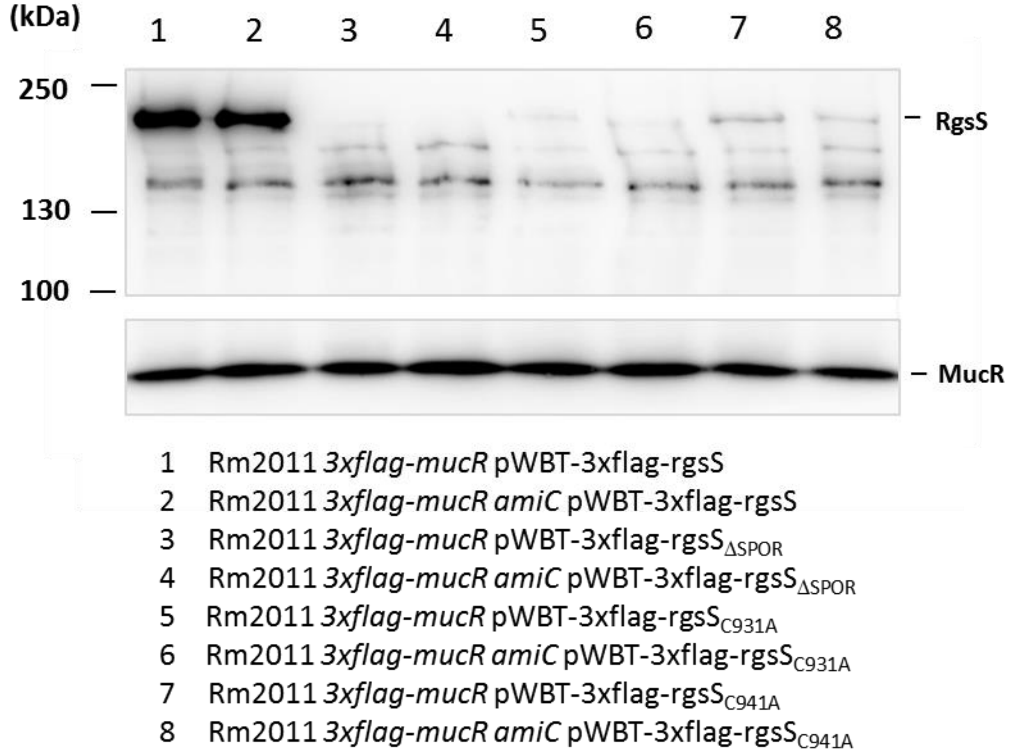

Supplementary Figure 9. Mutation in *amiC* does not affect abundance of RgsS, whereas truncation of the SPOR domain as well as C931A or C941A mutations strongly reduce RgsS levels. Western blot analysis of RgsS with α-FLAG antibody. Native and mutated 3xFLAG-RgsS was ectopically produced from multicopy expression vector pWBT in *amiC*-sufficient and *amiC*-deficient strains, exponentially growing in TY supplemented with gentamicin and 100 µM IPTG. 3×FLAG-MucR produced from *3xflag-mucR* at the native genomic location was used as a loading control. The result is representative of three biological replicates.

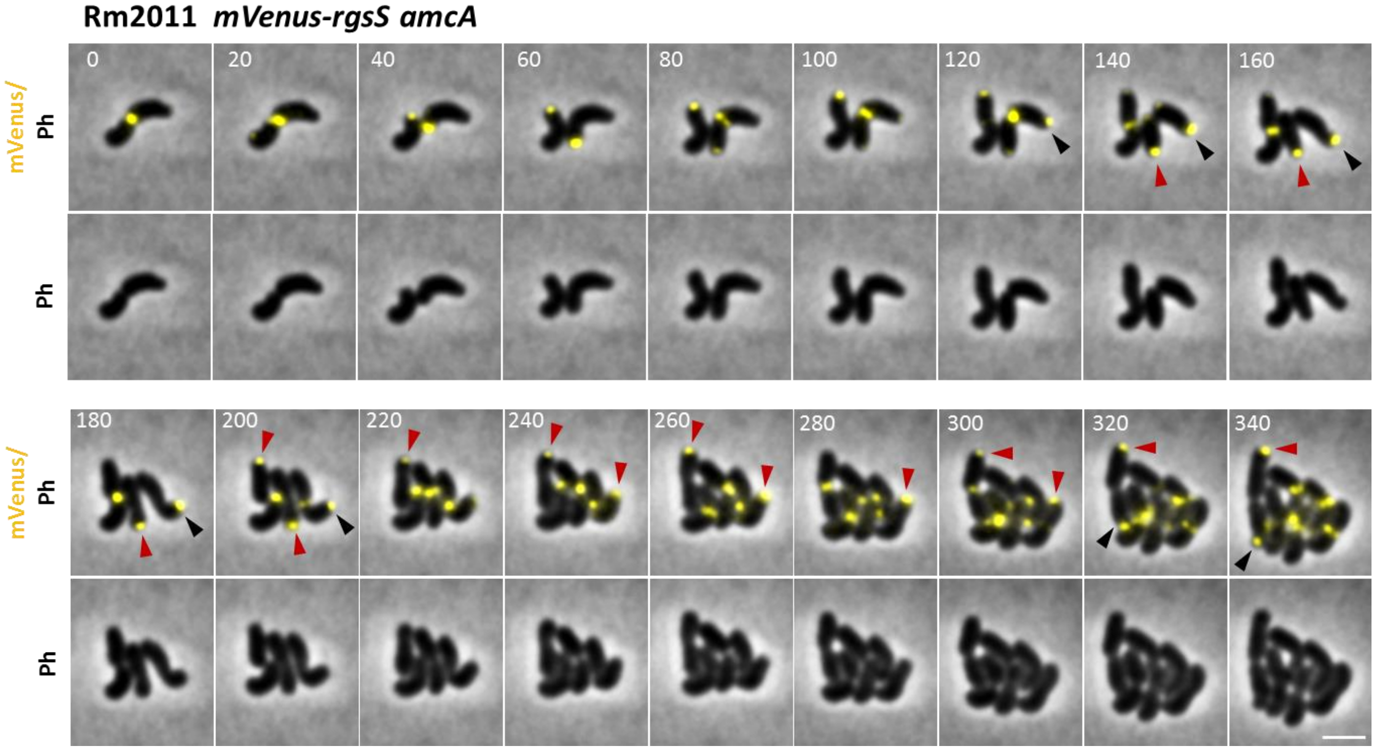

Supplementary Figure 10. Time lapse microscopy of Rm2011 *mVenus-rgsS amcA*, growing on MM-agarose pad. Red arrowheads point to cells with localization of an mVenus-RgsS focus at the old pole of the daughter cell and black arrowheads point to cells with localization of an mVenus-RgsS focus at the old pole of the mother cell. Time is shown in minutes. Scale bar, 2 µm. The images are representative of three independent cultivations and microscopy analyses, with at least 10 microcolonies imaged in parallel in each replicate.

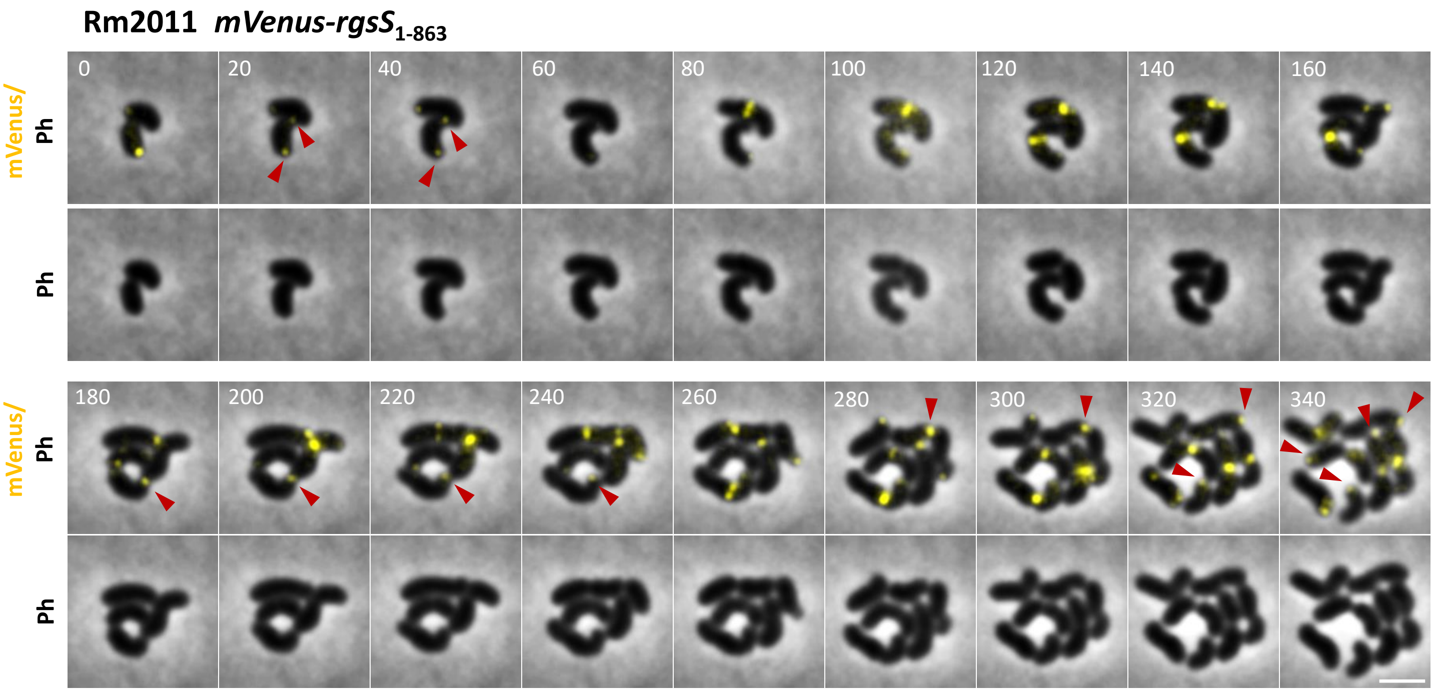

Supplementary Figure 11. Time lapse microscopy of Rm2011 *mVenus-rgsS*$_{1\text{-}863}$, growing on MM-agarose pad. Red arrowheads point to cells with mVenus-RgsS$_{1\text{-}863}$ focus localization at the old pole of the daughter cell or bipolar focus localization. Time is shown in minutes. Scale bar, 2 µm. The images are representative of three independent cultivations and microscopy analyses, with at least 10 microcolonies imaged in parallel in each replicate.

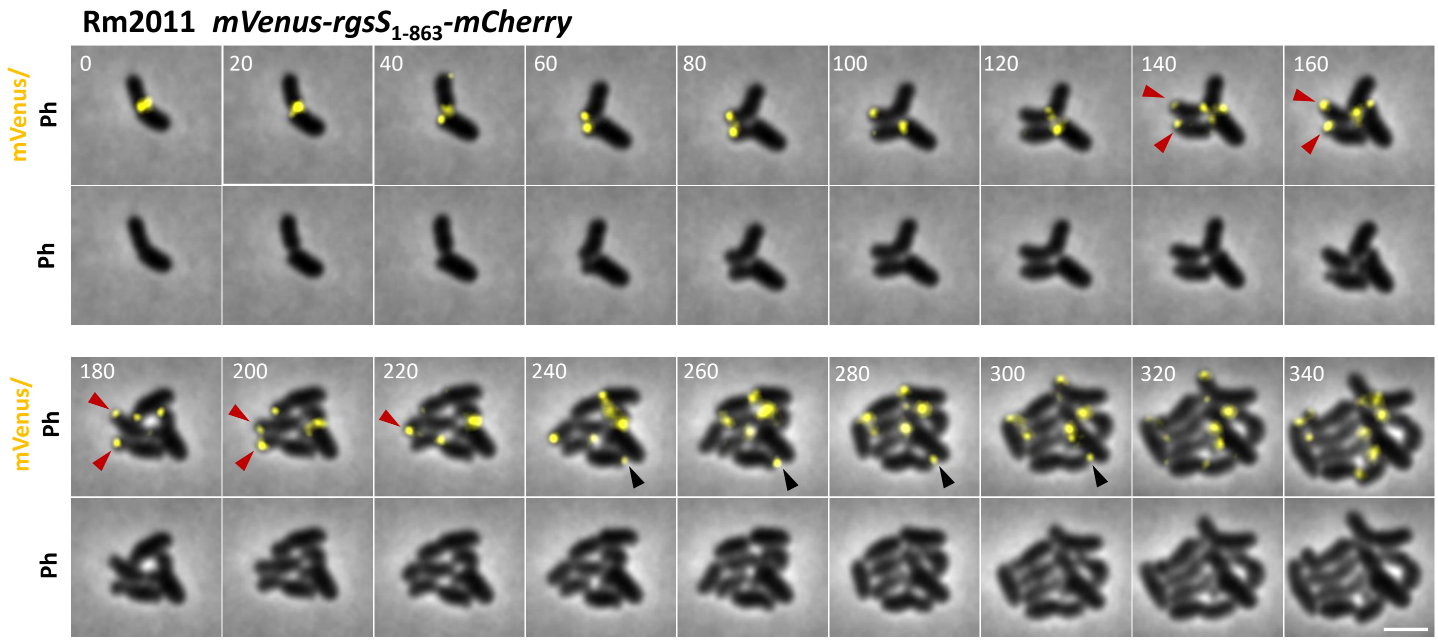

Supplementary Figure 12. Time lapse microscopy of Rm2011 *mVenus-rgsS*$_{1\text{-}863}$*-mCherry*, growing on MM-agarose pad. Red arrowheads point to cells with localization of an mVenus-RgsS$_{1\text{-}863}$-mCherry focus at the old pole of the daughter cell and black arrowheads point to cells with localization of an mVenus-RgsS$_{1\text{-}863}$-mCherry focus at the old pole of the mother cell. Time is shown in minutes. Scale bar, 2 µm. The images are representative of three independent cultivations and microscopy analyses, with at least 10 microcolonies imaged in parallel in each replicate.

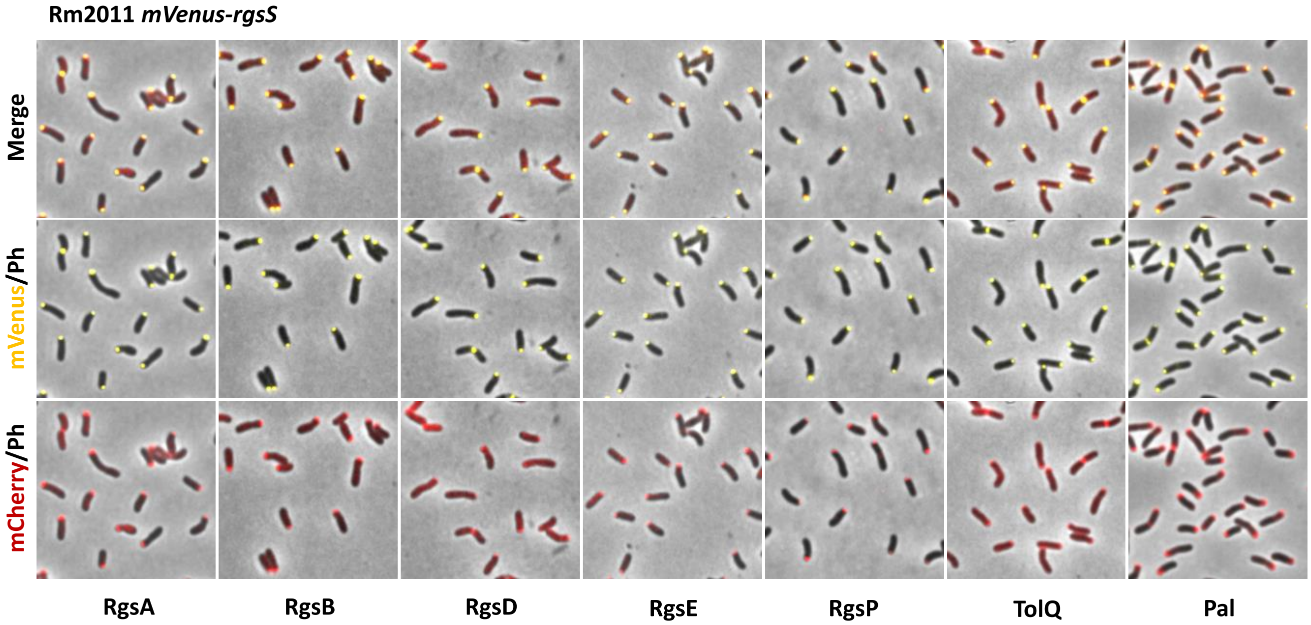

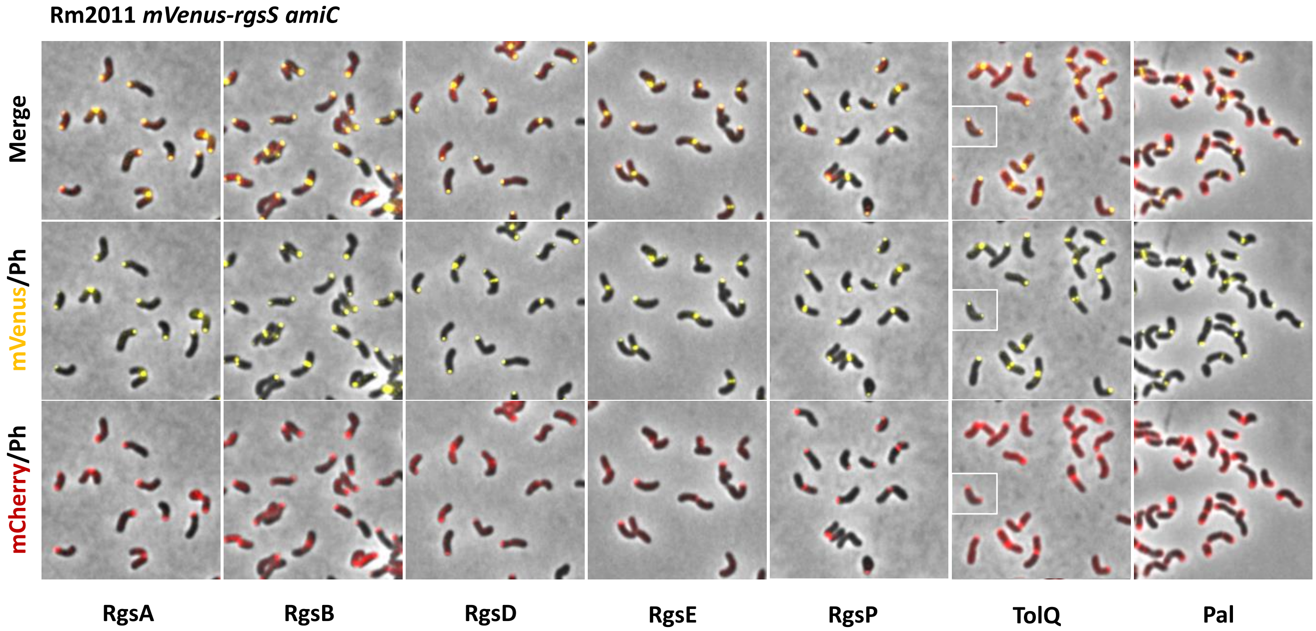

Supplementary Figure 13. Rgs proteins and TolQ, but not Pal colocalize with RgsS independent of *amiC*. Fluorescence microscopy images of Rm2011 *mVenus-rgsS* wild type and Rm2011 *mVenus-rgsS amiC* cells producing C-terminal mCherry fusions of the indicated proteins from gene fusions at the native genome locations. Cell samples were taken from exponential phase TY cultures. Scale bar, 5 µm; Ph, phase contrast. The insert shows an additional cell of the same strain representative of cells with bipolar colocalization of mVenus-RgsS and TolQ-mCherry fluorescence foci. The images are representative of two independent cultivations and microscopy analyses.

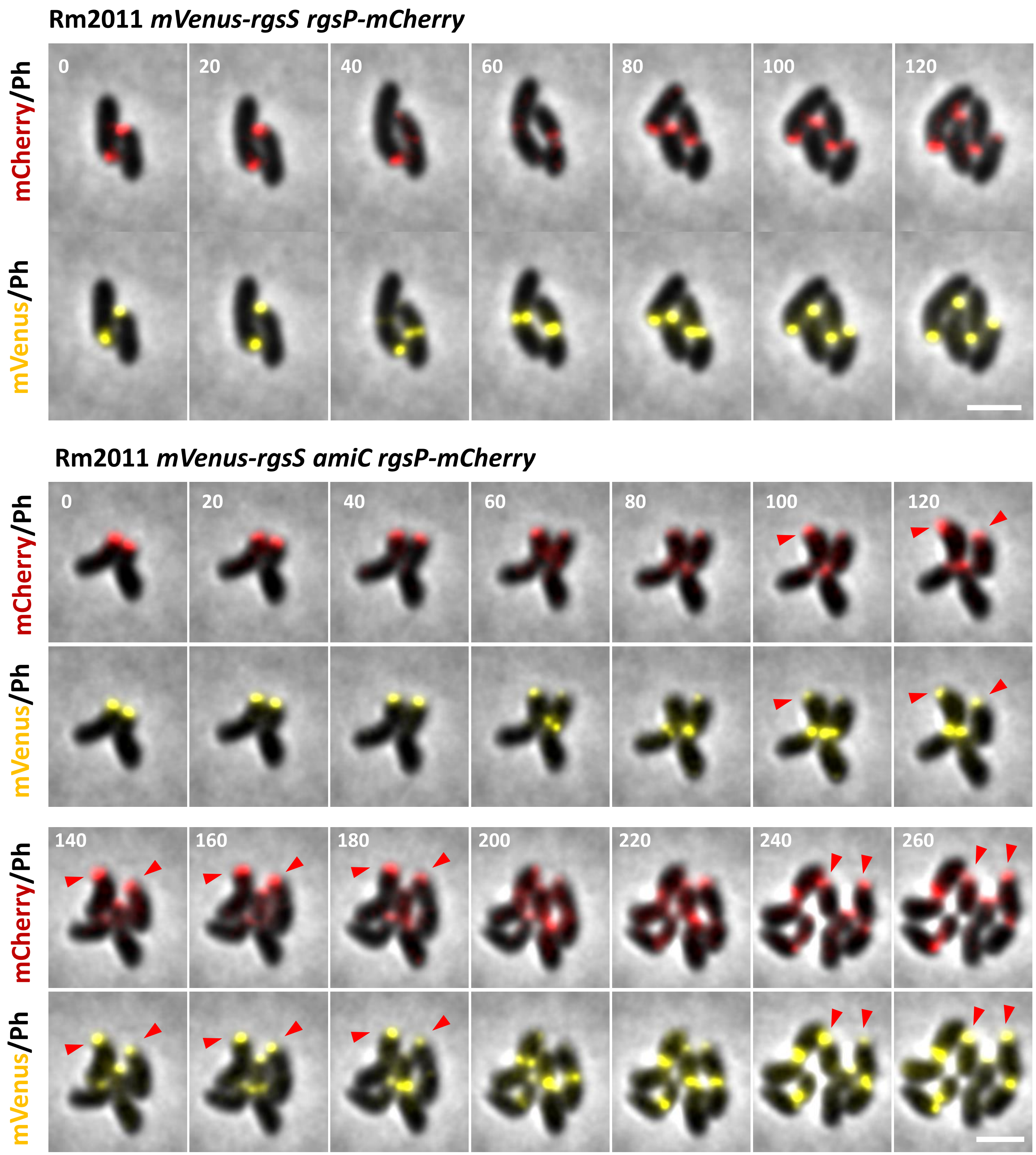

Supplementary Figure 14. RgsS and RgsP colocalize in both *amiC*-sufficient and *amiC*-deficient cells. Time-lapse fluorescence microscopy images of Rm2011 *mVenus-rgsS* wild type and its *amiC* mutant derivative carrying a *rgsP-mCherry* fusion at the native genome location. Cells were grown on MM-agarose. Red arrowheads indicate accumulation of mVenus-RgsS and RgsP-mCherry at the old cell pole of the daughter cell. Time is shown in minutes. Scale bar, 2 µm; Ph, phase contrast. The images are representative of two independent cultivations and microscopy analyses, with at least 8 microcolonies imaged in parallel in each replicate.

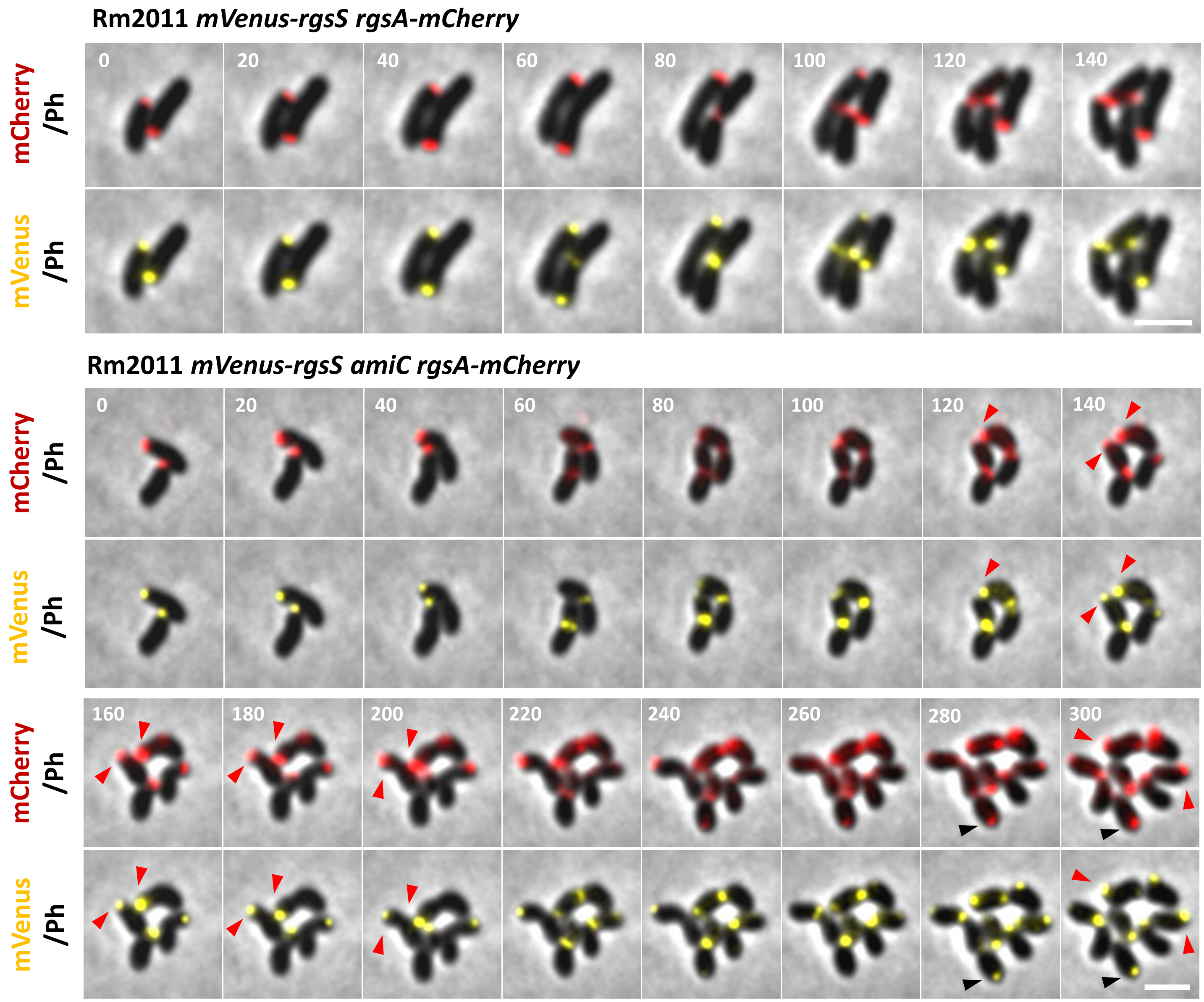

Supplementary Figure 15. RgsS and RgsA colocalize in both *amiC*-sufficient and *amiC*-deficient cells. Time-lapse fluorescence microscopy images of Rm2011 *mVenus-rgsS* wild type and its *amiC* mutant derivative carrying a *rgsA-mCherry* fusion at the native genome location. Cells were grown on MM-agarose. Red arrowheads indicate accumulation of mVenus-RgsS and RgsA-mCherry at the old cell pole of the daughter cell. Black arrowheads indicate accumulation of mVenus-RgsS and RgsA-mCherry at the old cell pole of the mother cell. Time is shown in minutes. Scale bar, 2 µm; Ph, phase contrast. The images are representative of two independent cultivations and microscopy analyses, with at least 8 microcolonies imaged in parallel in each replicate.

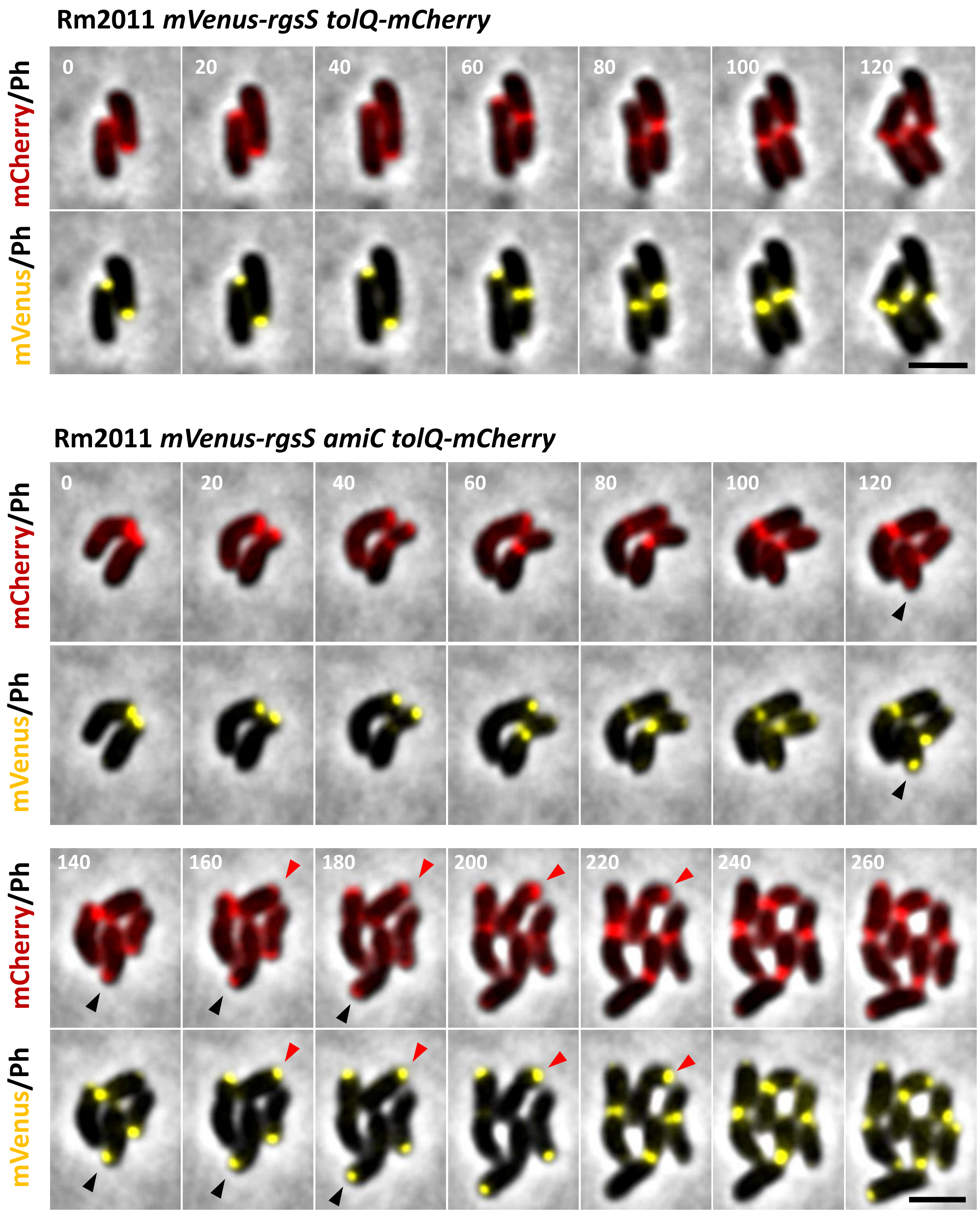

Supplementary Figure 16. RgsS and TolQ colocalize in both *amiC*-sufficient and *amiC*-deficient cells. Time-lapse fluorescence microscopy images of Rm2011 *mVenus-rgsS* wild type and its *amiC* mutant derivative carrying a *tolQ-mCherry* fusion at the native genome location. Cells were grown on MM-agarose. Red arrowheads indicate accumulation of mVenus-RgsS and TolQ-mCherry at the old cell pole of the daughter cell. Black arrowheads indicate accumulation of mVenus-RgsS and TolQ-mCherry at the old cell pole of the mother cell. Time is shown in minutes. Scale bar, 2 µm; Ph, phase contrast. The images are representative of two independent cultivations and microscopy analyses, with at least 8 microcolonies imaged in parallel in each replicate.

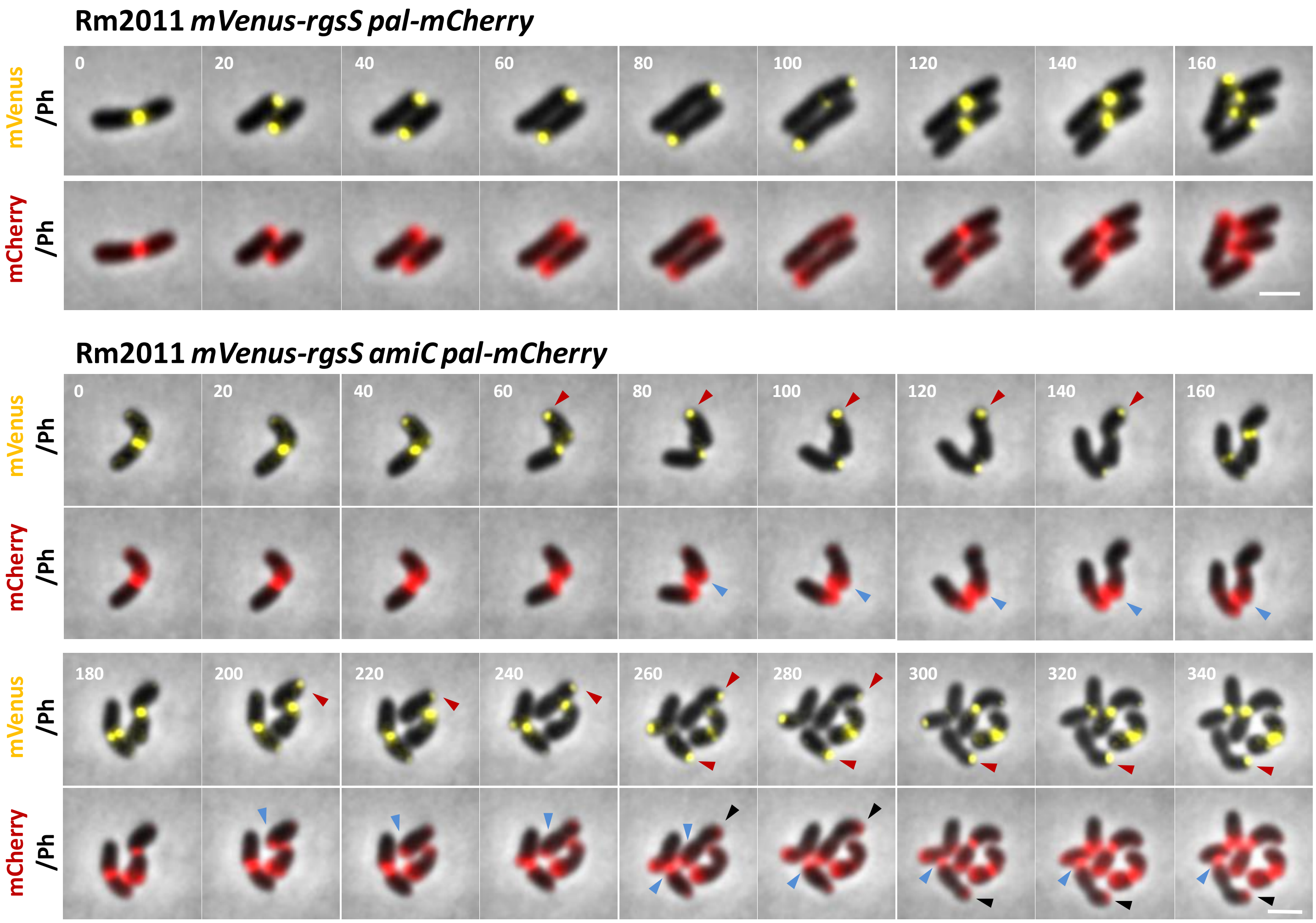

Supplementary Figure 17. RgsS and Pal consistently colocalize in *amiC*-sufficient, but not in *amiC*-deficient cells. Time-lapse fluorescence microscopy images of Rm2011 *mVenus-rgsS* wild type and its *amiC* mutant derivative carrying a *pal-mCherry* fusion at the native genome location. Cells were grown on MM-agarose. Red arrowheads indicate aberrant localization of mVenus-RgsS. Blue arrowheads indicate cells that retained Pal-mCherry foci at the new cell pole despite accumulation of mVenus-RgsS at the old cell pole. Black arrowheads denote Pal-mCherry foci that emerged *de novo* at the old cell pole containing mVenus-RgsS focus. Time is shown in minutes. Scale bar, 2 µm; Ph, phase contrast. The images are representative of two independent cultivations and microscopy analyses, with at least 8 microcolonies imaged in parallel in each replicate.

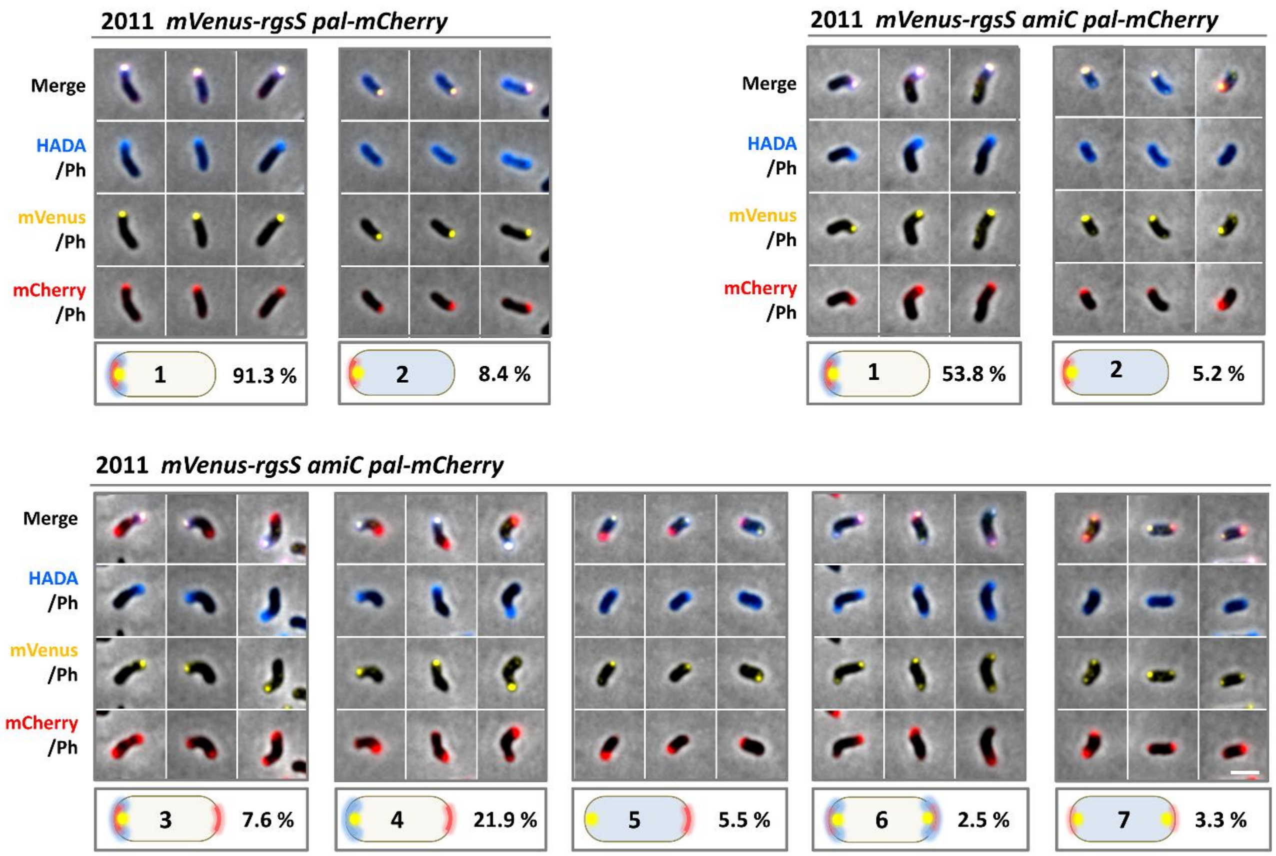

Supplementary Figure 18. Representative fluorescence microscopy images of HADA pulse-labeled Rm2011 *mVenus-rgsS* wild type and its *amiC* mutant derivative carrying a *pal-mCherry* fusion at the native genome location. The cell types are shown as sketches with numbering according to Supplementary Table 6. Types 6 and 7 include cells with either unipolar or bipolar Pal-mCherry localization. Percentage values were calculated considering only cells with polar fluorescence foci and represent the mean values of three biological replicates. The standard deviation values and statistic comparison between *amiC*-sufficient and *amiC*-deficient strains are shown in Supplementary Table 6. Ph, phase contrast. Scale bar, 2 µm. The images are representative of three independent cultivations, HADA staining and microscopy analyses.

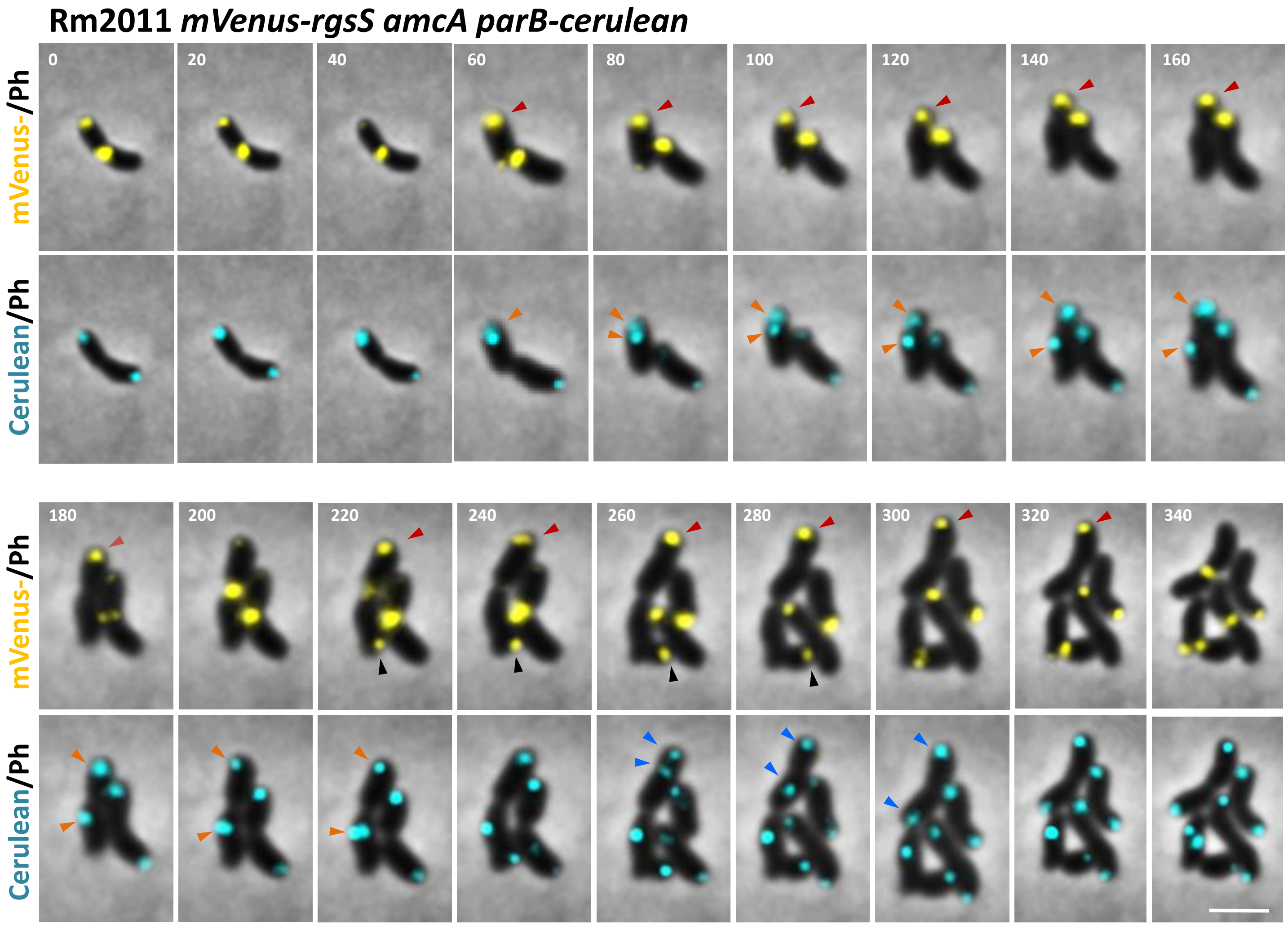

Supplementary Figure 19. Effects of inverted cell growth polarity on segregation of chromosomal origins. Time-lapse fluorescence microscopy of Rm2011 *mVenus-rgsS amcA*, carrying *parB-cerulean* at the native genome location, growing on MM-agarose. Red and black arrowheads indicate accumulation of mVenus-RgsS at the old cell pole of the daughter cell (red) or a mother cell (black). Blue and orange arrowheads show complete (blue) or incomplete (orange) ParB-cerulean focus relocation to the opposite cell pole of a cell with inverted growth polarity. In the frames at 200 and 220 minutes, occurrence of an mVenus-RgsS focus at the older cell pole and vanishing of the mVenus-RgsS focus at the newly formed cell pole can be observed in a cell that inherited the ParB-cerulean focus at the new cell pole. Time is indicated in minutes. Scale bar, 2 µm; Ph, phase contrast. The images are representative of four independent cultivations and microscopy analyses, with at least 10 microcolonies imaged in parallel in each replicate.

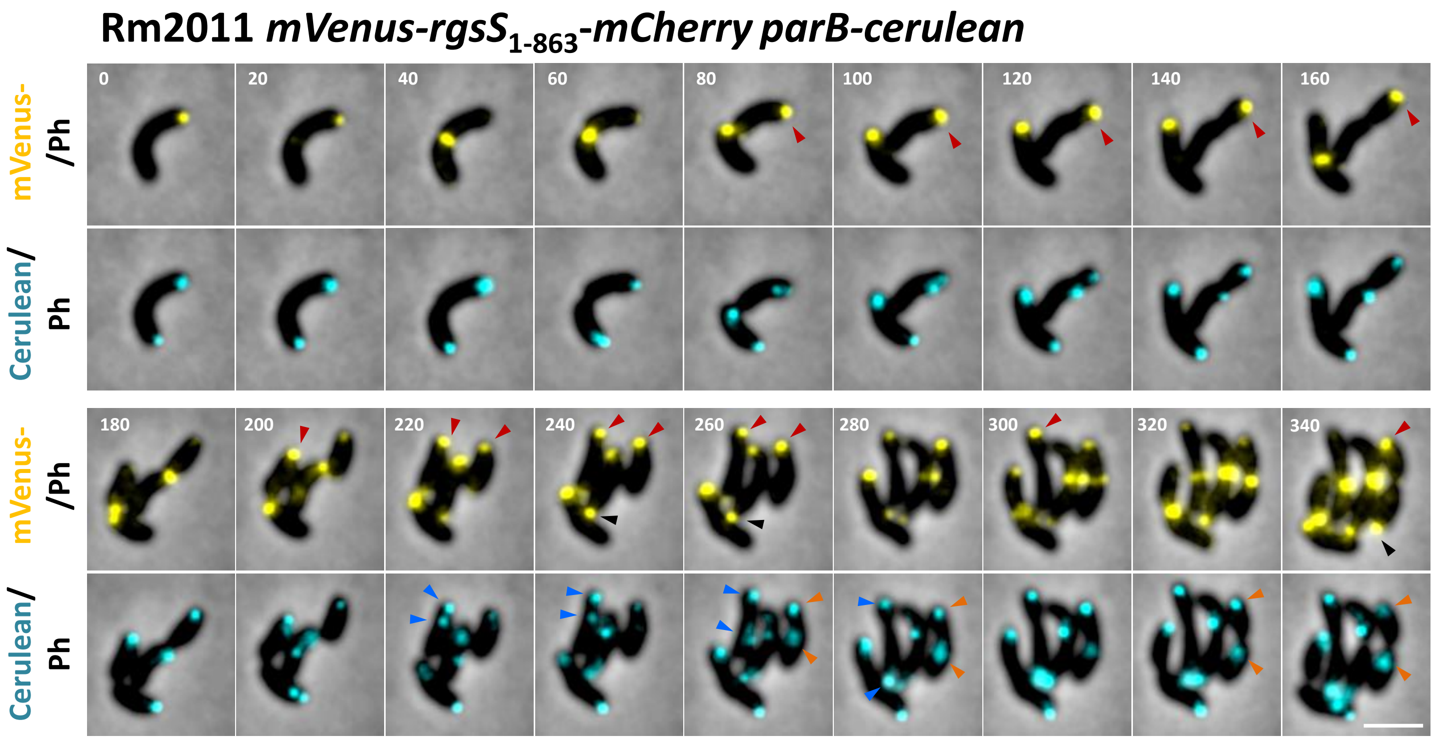

Supplementary Figure 20. Effects of inverted cell growth polarity on segregation of chromosomal origins. Time-lapse fluorescence microscopy of Rm2011 *mVenus-rgsS*$_{1\text{-}863}$*-mCherry*, carrying *parB-cerulean* at the native genome location, growing on MM-agarose pad. Red and black arrowheads indicate occurrence of the mVenus-RgsS focus at the old cell pole of the daughter cell (red) or a mother cell (black) after cell division. Blue and orange arrowheads show complete (blue) or incomplete (orange) ParB-cerulean focus relocation to the opposite cell pole of a cell with inverted growth polarity. In the frames at 200/240 and 320/340 minutes, occurrence of an mVenus-RgsS focus at the older cell pole and vanishing of the mVenus-RgsS focus at the newly formed cell pole can be observed in a cell that inherited the ParB-cerulean focus at the new cell pole. Time is indicated in minutes. Scale bar, 2 µm; Ph, phase contrast. The images are representative of four independent cultivations and microscopy analyses, with at least 10 microcolonies imaged in parallel in each replicate.

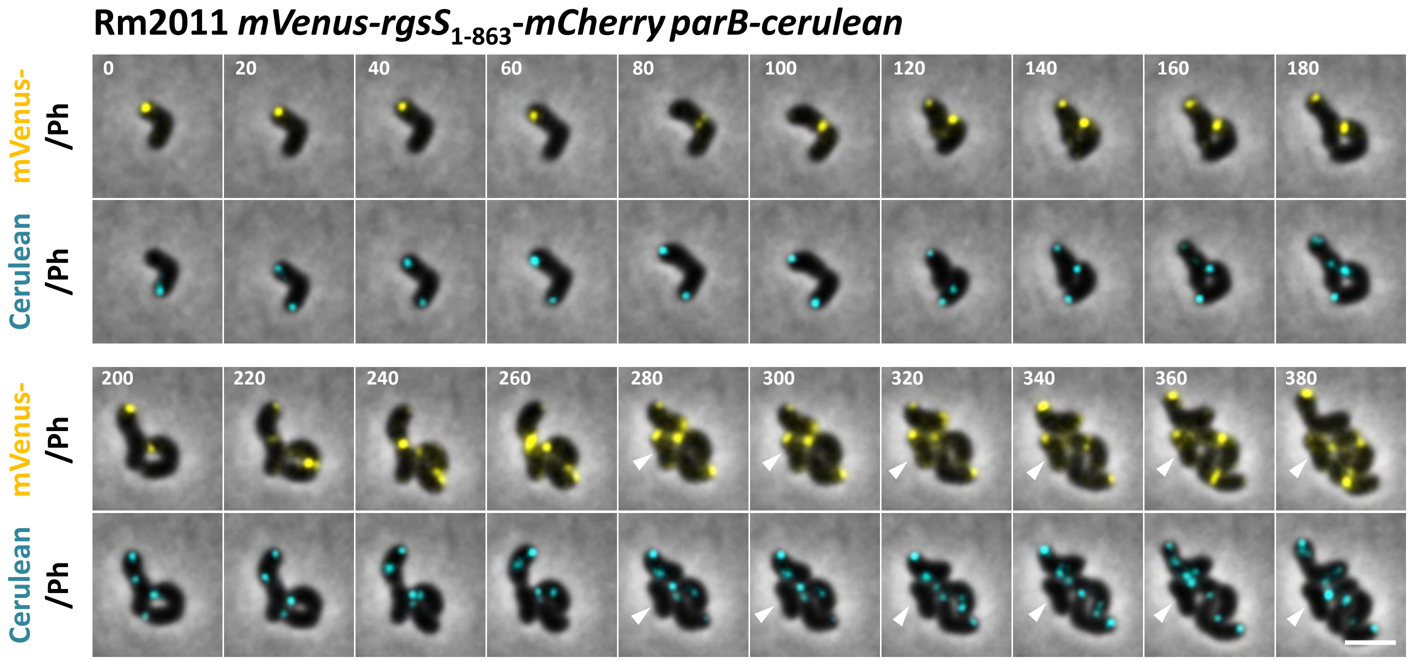

Supplementary Figure 21. Formation of a cell lacking a ParB-cerulean focus upon incomplete relocation of *oriC*$_2$ towards the non-growing pole. Time-lapse fluorescence microscopy images of Rm2011 *mVenus-rgsS*$_{1\text{-}863}$*-mCherry* carrying *parB-cerulean* at the native genome location growing on MM-agarose. Arrowheads indicate the cell lacking a ParB-cerulean focus. This cell did not elongate and lost the mVenus-RgsS focus. Time is shown in minutes. Scale bar, 2 µm; Ph, phase contrast. The images are representative of four microcolonies in which the formation of a cell without ParB-cerulean focus was observed, among a total of 85 analyzed microcolonies.

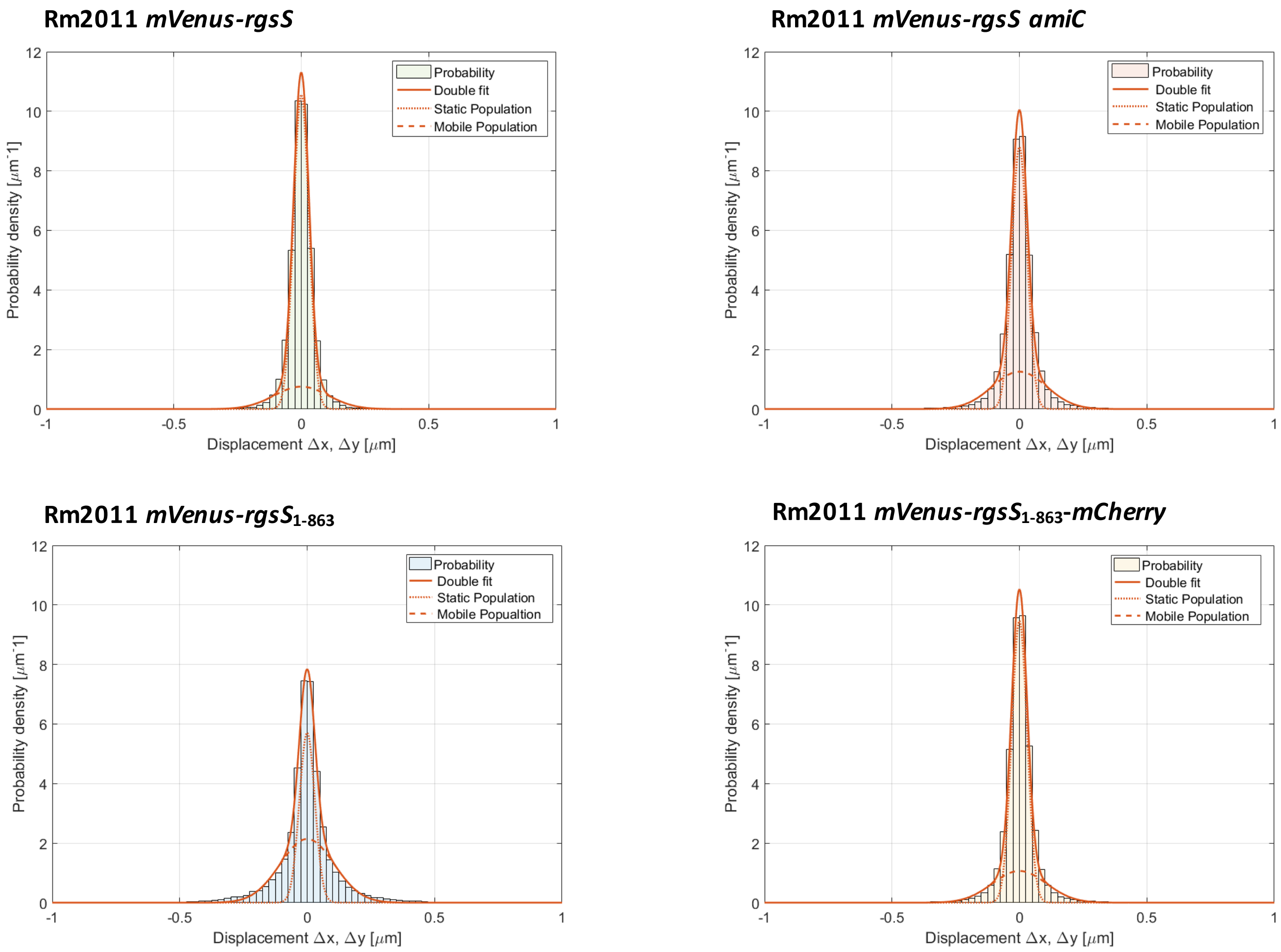

Supplementary Figure 22. Fitting of the single-molecule tracking data into Gaussian mixture model revealed two molecule fractions with distinct diffusion coefficients. The distribution of probability density of frame-to-frame molecule displacement is shown. The displacement illustrates the directionality of trajectories in x and y direction of the cell axis relative to the cell center. Solid red line indicates the Gaussian fit of two populations. A dashed line indicates the fit of the mobile fraction and a dotted line the static fraction of the trajectories.

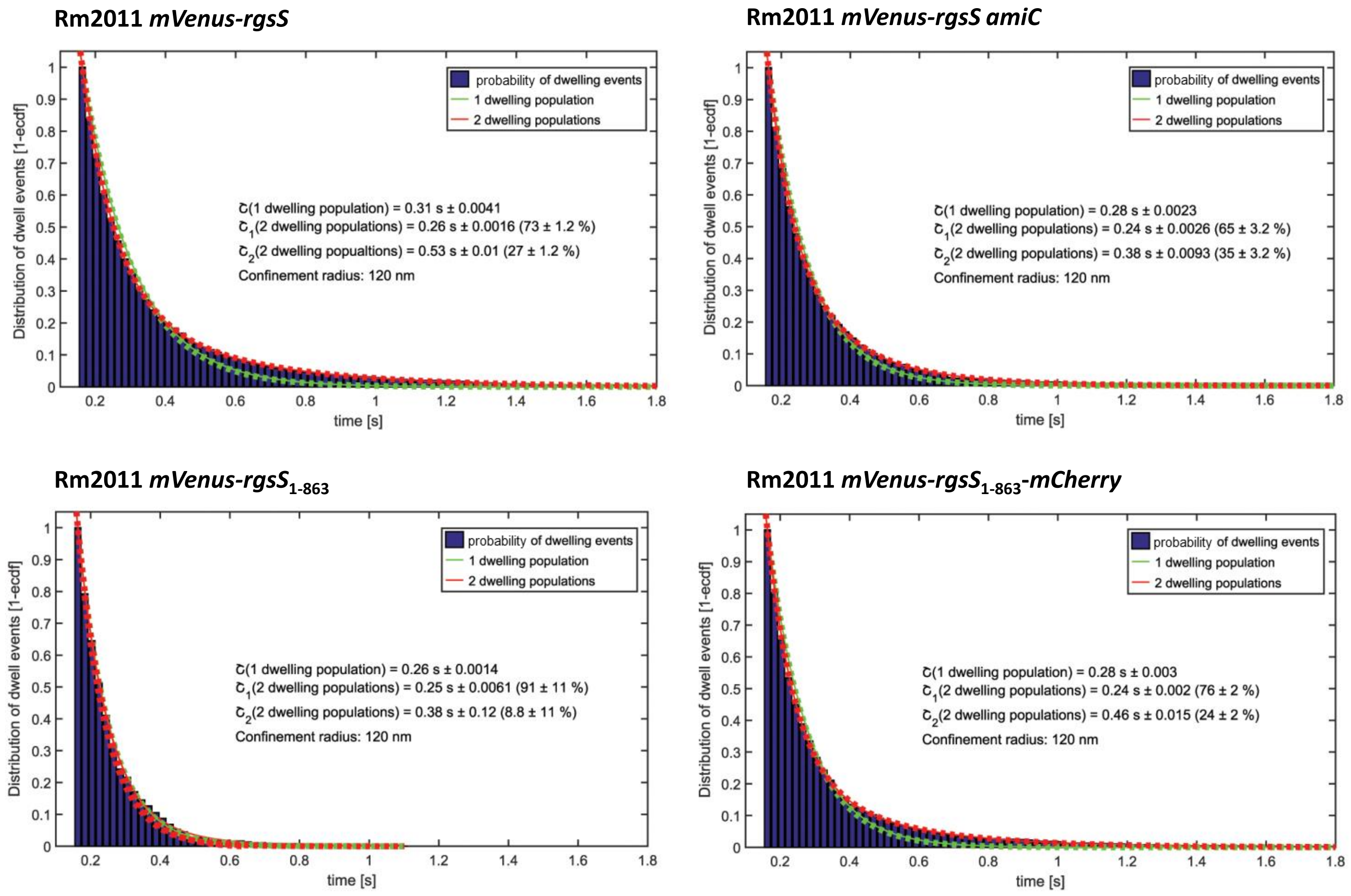

Supplementary Figure 23. Determination of residence times. Fitting of the empirical cumulative distribution of dwell events into the decay curves identified two dwelling populations. One-component fit corresponding to one dwelling population is shown in green and a two-component fit corresponding to two dwelling populations is shown in red. ecdf, empirical cumulative distribution function. τ, residence time. Dwell events were defined by trajectories staying in the confinement radius of 120 nm for a minimum of 9 steps.

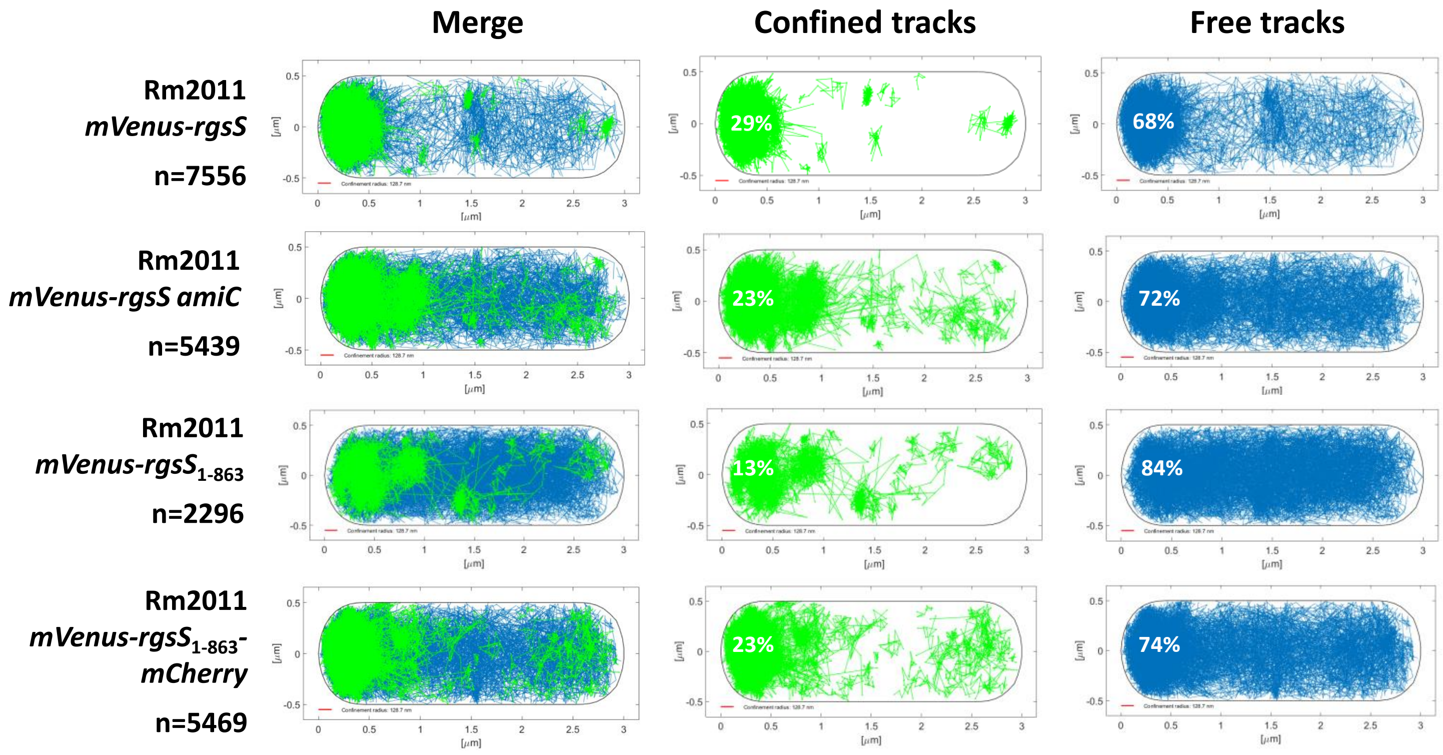

Supplementary Figure 24. Single-molecule tracking identified molecules with different spatiotemporal behavior. Projections of single molecule trajectories in a normalized cell are shown. Cell samples were taken from exponential phase TY cultures. n, number of single molecule tracks analyzed. Confined tracks represent molecules that were detected within the confinement radius of 120 nm for 9 consecutive 20 msec steps or longer. Free tracks show molecules that left the confinement radius at each of the 9 or more consecutive 20 msec steps. The percentage of tracks with each behavior is indicated. Tracks with a transitional behavior are not shown. Numerical data is shown in Supplementary Table 9.

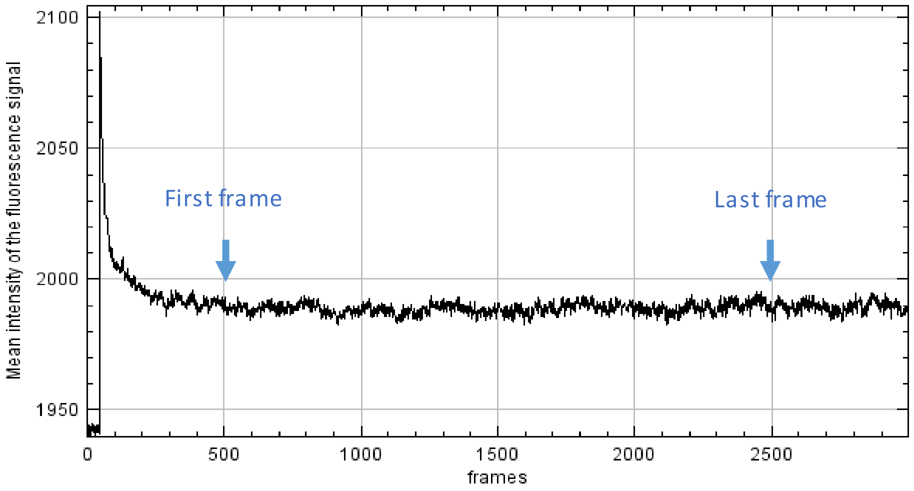

Supplementary Figure 25. Representative bleaching curve generated during single molecule tracking microscopy of mVenus-RgsS. The data originate from the set of images constituting the Supplementary movie 1. Arrows indicate the range of frames used for analysis.

Supplementary Table 1. Localization pattern of mVenus-RgsS and mCherry-SPOR$_{RgsS}$ in Rm2011 *mVenus-rgsS* pSRKGm-SP-mCherry-SPOR cells from exponential TY cultures. Diffuse mCherry fluorescence was detected in all the considered cells. Percentage values from three independent biological replicates (373-491 cells each) were used to determine the mean percentage values. SD, standard deviation of the mean.

**Strain**		**Rm2011** ***mVenus-rgsS***
**mVenus-RgsS and mCherry-SPOR$_{RgsS}$ fluorescence foci at the same cell pole, % (± SD)**	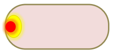	50.02 (± 4.78)
**mVenus-RgsS fluorescence focus at one cell pole, no mCherry-SPOR$_{RgsS}$ fluorescence focus, % (± SD)**	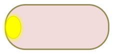	35.30 (± 3.93)
**mVenus-RgsS and mCherry-SPOR$_{RgsS}$ fluorescence foci at the septum, mVenus-RgsS fluorescence focus at the pole, % (± SD)**	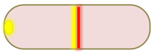	4.57 (± 1.77)
**mVenus-RgsS and mCherry-SPOR$_{RgsS}$ fluorescence foci at the septum, % (± SD)**	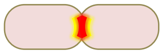	10.11 (± 2.08)
**n (cells)**		1314

Supplementary Table 2. Cell shape features of the Rm2011 *mVenus-rgsS* strain and its derivatives in exponential TY cultures. Average values from three independent biological replicates (500 cells each) were used to determine the mean values. SD, standard deviation of the mean. The significance of the differences between the strains was determined in a two-tailed t-test with unequal variance. P≥0.05, non-significant (ns); P<0.05, *; P<0.01, **; P<0.001, ***.

**Strain**	**Rm2011 *mVenus-rgsS***	**Rm2011 *mVenus-rgsS amiD***	**Rm2011 *mVenus-rgsS amiC***	**Rm2011 *mVenus-rgsS amcA***	**Rm2011 *mVenus-* $rgsS_{1-863}$**	**Rm2011 *mVenus-* $rgsS_{1-863}$*-mCherry***
**Average length, µm (± SD)**	2.80 (± 0.07)	2.69 (± 0.08)	2.65 (± 0.06)	2.66 (± 0.06)	2.53 (± 0.09)	2.64 (± 0.11)
**Average width, µm (± SD)**	0.70 (± 0.01)	0.72 (± 0.02)	0.76 (± 0.01)	0.76 (± 0.01)	0.79 (± 0.01)	0.74 (± 0.01)
**Average curvature (± SD)**	0.18 (± 0.01)	0.19 (± 0.04)	0.44 (± 0.02)	0.44 (± 0.02)	0.57 (± 0.02)	0.48 (± 0.02)
**n (cells)**	1500	1500	1500	1500	1500	1500

Significance of differences in average cell length

	**P-value/significance level**					
	**Rm2011 *mVenus-rgsS***	**Rm2011 *mVenus-rgsS amiD***	**Rm2011 *mVenus-rgsS amiC***	**Rm2011 *mVenus-rgsS amcA***	**Rm2011 *mVenus-* $rgsS_{1-863}$**	**Rm2011 *mVenus-* $rgsS_{1-863}$*-mCherry***
**Rm2011 *mVenus-rgsS***		1.53E-01/ns	5.44E-02/ns	5.51E-02/ns	1.58E-02/*	2.16E-02/*
**Rm2011 *mVenus-rgsS amiD***			4.13E-01/ns	5.68E-01/ns	7.11E-02/ns	1.10E-01/ns
**Rm2011 *mVenus-rgsS amiC***				7.73E-01/ns	1.37E-01/ns	5.07E-02/ns
**Rm2011 *mVenus-rgsS amcA***					1.08E-01/ns	1.19E-01/ns
**Rm2011 *mVenus-* $rgsS_{1-863}$**						4.29E-01/ns

Significance of differences in average cell width

	**P-value/significance level**					
	**Rm2011 *mVenus-rgsS***	**Rm2011 *mVenus-rgsS amiD***	**Rm2011 *mVenus-rgsS amiC***	**Rm2011 *mVenus-rgsS amcA***	**Rm2011 *mVenus-* $rgsS_{1-863}$**	**Rm2011 *mVenus-* $rgsS_{1-863}$*-mCherry***
**Rm2011 *mVenus-rgsS***		1.90E-01/ns	2.16E-04/***	2.84E-03/**	3.29E-03/**	1.83E-02/*
**Rm2011 *mVenus-rgsS amiD***			6.30E-02/ns	4.85E-02/*	6.41E-03/**	1.62E-01/ns
**Rm2011 *mVenus-rgsS amiC***				9.86E-01/ns	4.17E-02/*	1.28E-01/ns
**Rm2011 *mVenus-rgsS amcA***					2.89E-02/*	1.23E-01/ns
**Rm2011 *mVenus-* $rgsS_{1-863}$**						1.33E-02/*

Significance of differences in average cell curvature

	**P-value/significance**					
	**Rm2011 *mVenus-rgsS***	**Rm2011 *mVenus-rgsS amiD***	**Rm2011 *mVenus-rgsS amiC***	**Rm2011 *mVenus-rgsS amcA***	**Rm2011 *mVenus-* $rgsS_{1-863}$**	**Rm2011 *mVenus-* $rgsS_{1-863}$*-mCherry***
**Rm2011 *mVenus-rgsS***		6.25E-01/ns	7.83E-03/**	1.75E-03/**	1.48E-04/***	2.49E-05/***
**Rm2011 *mVenus-rgsS amiD***			1.85E-03/**	1.55E-03/**	1.05E-03/**	6.30E-03/**
**Rm2011 *mVenus-rgsS amiC***				8.44E-01/ns	2.20E-02/*	2.98E-01/ns
**Rm2011 *mVenus-rgsS amcA***					3.11E-03/**	1.85E-01/ns
**Rm2011 *mVenus-* $rgsS_{1-863}$**						8.88E-03/**

Supplementary Table 3. Localization of mVenus-RgsS and its truncated variants mVenus-RgsS$_{1\text{-}863}$ and mVenus-RgsS$_{1\text{-}863}$-mCherry in cells growing exponentially in TY medium. Percentage values from three independent biological replicates (402 to 592 cells each) were used to determine the mean percentage values. SD, standard deviation of the mean. The significance of the differences between the strains was determined in a two-tailed t-test with unequal variance. P≥0.05, non-significant (ns); P<0.05, *; P<0.01, **; P<0.001, ***. In case of the Rm2011 *mVenus-rgsS*$_{1\text{-}863}$ strain, 21.44 % (± 2.90) of the cells did not contain detectable fluorescence foci.

**Strain**	**Rm2011 *mVenus-rgsS***	**Rm2011 *mVenus-rgsS amiC***	**Rm2011 *mVenus-rgsS amcA***	**Rm2011 *mVenus-rgsS*$_{1\text{-}863}$**	**Rm2011 *mVenus-rgsS*$_{1\text{-}863}$*-mCherry***
**Unipolar localization, % (± SD)**	86.69 (± 0.24)	74.68 (± 1.39)	75.04 (± 1.45)	59.65 (± 2.98)	77.12 (± 0.23)
**Septal and unipolar localization, % (± SD)**	4.14 (± 0.07)	4.09 (± 0.51)	5.16 (± 0.46)	1.65 (± 0.23)	4.55 (± 1.01)
**Septal localization, % (± SD)**	9.17 (± 0.22)	15.98 (± 1.24)	15.46 (± 1.75)	15.86 (± 0.65)	11.87 (± 1.77)
**Bipolar localization, % (± SD)**	0.00 (± 0.00)	5.13 (± 0.95)	4.34 (± 0.13)	1.40 (± 0.67)	6.47 (± 1.50)
**n (cells)**	1451	1614	1682	1639	1638

Significance of differences in average unipolar localization

	**P-value/significance level**				
	**Rm2011 *mVenus-rgsS***	**Rm2011 *mVenus-rgsS amiC***	**Rm2011 *mVenus-rgsS amcA***	**Rm2011 *mVenus- rgsS*$_{1\text{-}863}$**	**Rm2011 *mVenus-rgsS*$_{1\text{-}863}$*-mCherry***
**Rm2011 *mVenus-rgsS***		3.57E-03/**	4.20E-03/**	3.82E-03/**	1.05E-06/***
**Rm2011 *mVenus-rgsS amiC***			7.69E-01/ns	5.13E-03/**	8.94E-02/ns
**Rm2011 *mVenus-rgsS amcA***				4.54E-03/**	1.27E-01/ns
**Rm2011 *mVenus- rgsS*$_{1\text{-}863}$**					9.20E-03/**

Significance of differences in average septal and polar localization

	**P-value/significance level**				
	**Rm2011 *mVenus-rgsS***	**Rm2011 *mVenus-rgsS amiC***	**Rm2011 *mVenus-rgsS amcA***	**Rm2011 *mVenus- rgsS*$_{1\text{-}863}$**	**Rm2011 *mVenus-rgsS*$_{1\text{-}863}$*-mCherry***
**Rm2011 *mVenus-rgsS***		8.64E-01/ns	5.83E-02/ns	1.35E-03/**	5.59E-01/ns
**Rm2011 *mVenus-rgsS amiC***			5.38E-02/ns	6.00E-03/**	5.30E-01/ns
**Rm2011 *mVenus-rgsS amcA***				1.36E-03/**	4.16E-01/ns
**Rm2011 *mVenus- rgsS*$_{1\text{-}863}$**					3.27E-02/*

Significance of differences in average septal localization

	**P-value/significance level**				
	**Rm2011 *mVenus-rgsS***	**Rm2011 *mVenus-rgsS amiC***	**Rm2011 *mVenus-rgsS amcA***	**Rm2011 *mVenus- rgsS*$_{1\text{-}863}$**	**Rm2011 *mVenus-rgsS*$_{1\text{-}863}$*-mCherry***
**Rm2011 *mVenus-rgsS***		9.16E-03/**	2.34E-02/*	1.32E-03/**	1.17E-01/ns
**Rm2011 *mVenus-rgsS amiC***			7.01E-01/ns	8.95E-01/ns	3.55E-02/*
**Rm2011 *mVenus-rgsS amcA***				7.40E-01/ns	6.67E-02/ns
**Rm2011 *mVenus- rgsS*$_{1\text{-}863}$**					4.65E-02/*

Supplementary Table 4. Doubling time and duration of septal localization of mVenus-RgsS estimated in cells growing on MM agarose pad in time-lapse analysis. Average values from three independent biological replicates (79 to 228 cells/cell divisions each) were used to determine the mean values. SD, standard deviation of the mean. The significance of the differences between the strains was determined in a two-tailed t-test with unequal variance. P≥0.05, non-significant (ns); P<0.05, *; P<0.01, **; P<0.001, ***.

**Strain**	**Rm2011 *mVenus-rgsS***	**Rm2011 *mVenus-rgsS amiC***	**Rm2011 *mVenus-rgsS amcA***	**Rm2011 *mVenus- rgsS*$_{1\text{-}863}$**	**Rm2011 *mVenus-rgsS*$_{1\text{-}863}$*-mCherry***
**Doubling time, min (± SD)**	117 (± 0.89)	131 (± 0.32)	131 (± 0.36)	124 (± 3.15)	121 (± 2.62)
**n (cell divisions)**	432	388	532	355	425
**Septal mVenus-RgsS localization, min (± SD)**	31 (± 0.99)	51 (± 1.64)	51 (± 0.81)	37 (± 0.9)	38 (± 0.82)
**n (cells)**	426	395	561	352	448

Significance of differences in doubling time

	**p-value/significance level**				
	**Rm2011 *mVenus-rgsS***	**Rm2011 *mVenus-rgsS amiC***	**Rm2011 *mVenus-rgsS amcA***	**Rm2011 *mVenus- rgsS*$_{1\text{-}863}$**	**Rm2011 *mVenus-rgsS*$_{1\text{-}863}$*-mCherry***
**Rm2011 *mVenus-rgsS amiC***		4.20E-04/***	2.96E-04/***	5.73E-02/ns	1.34E-01/ns
**Rm2011 *mVenus-rgsS amiC***			2.64E-01/ns	5.48E-02/ns	1.88E-02/ns
**Rm2011 *mVenus-rgsS amcA***				4.97E-02/ns	1.73E-02/ns
**Rm2011 *mVenus- rgsS*$_{1\text{-}863}$**					2.53E-01/ns

Significance of differences in duration of mVenus-RgsS septal localization

	**p-value/significance level**				
	**Rm2011 *mVenus-rgsS***	**Rm2011 *mVenus-rgsS amiC***	**Rm2011 *mVenus-rgsS amcA***	**Rm2011 *mVenus- rgsS*$_{1\text{-}863}$**	**Rm2011 *mVenus-rgsS*$_{1\text{-}863}$*-mCherry***
**Rm2011 *mVenus-rgsS amiC***		2.13E-04/***	1.60E-05/***	1.80E-03/**	1.01E-03/**
**Rm2011 *mVenus-rgsS amiC***			9.50E-01/ns	8.19E-04/***	1.24E-03/**
**Rm2011 *mVenus-rgsS amcA***				3.81E-05/***	3.70E-05/***
**Rm2011 *mVenus- rgsS*$_{1\text{-}863}$**					2.74E-01/ns

Supplementary Table 5. Polar localization of mVenus-RgsS and its truncated variants mVenus-RgsS$_{1-863}$ and mVenus-RgsS$_{1-863}$-mCherry in cells growing on a MM agarose pad observed in time-lapse analysis. Percentage values from three independent biological replicates (15 to 32 microcolonies each) were used to determine the mean percentage values. SD, standard deviation of the mean. The significance of the differences between the strains was determined in a two-tailed t-test with unequal variance. P≥0.05, non-significant (ns); P<0.05, *; P<0.01, **; P<0.001, ***.

**Strain**	**Rm2011 *mVenus-rgsS***	**Rm2011 *mVenus-rgsS amiC***	**Rm2011 *mVenus-rgsS amcA***	**Rm2011 *mVenus - rgsS$_{1-863}$***	**Rm2011 *mVenus-rgsS$_{1-863}$-mCherry***
**Daughter cells with the fluorescence focus at the new cell pole, mean % (± SD)**	50.00 (± 0.00)	22.26 (± 2.01)	24.62 (± 2.79)	20.54 (± 1.81)	24.23 (± 1.54)
**Daughter cells with the fluorescence focus at both cell poles, mean % (± SD)**	0.00 (± 0.00)	1.50 (± 0.25)	1.27 (± 0.83)	0.12 (± 0.21)	2.67 (± 0.79)
**Daughter cells with the fluorescence focus at the old cell pole, mean % (± SD)**	0.00 (± 0.00)	26.24 (± 1.76)	24.11 (± 2.32)	29.34 (± 1.91)	23.10 (± 0.88)
**Mother cells with mVenus fluorescence focus at the new cell pole, mean % (± SD)**	50.00 (± 0.00)	40.81 (± 0.72)	42.24 (± 1.84)	43.26 (± 1.37)	43.49 (± 0.65)
**Mother cells with the fluorescence focus at both cell poles, mean % (± SD)**	0.00 (± 0.00)	0.51 (± 0.19)	0.33 (± 0.35)	0.00 (± 0.00)	0.53 (± 0.71)
**Mother cells with mVenus fluorescence focus at the old cell pole, mean % (± SD)**	00.00 (± 0.00)	8.69 (± 0.66)	7.43 (± 1.80)	6.74 (± 1.37)	5.98 (± 1.31)
**n (microcolonies)**	56	81	67	60	67
**n (cells)**	868	998	984	678	846

Significance of differences in signal localization at the old cell pole in daughter cells

	**P-value/significance level**				
	**Rm2011 *mVenus-rgsS***	**Rm2011 *mVenus-rgsS amiC***	**Rm2011 *mVenus-rgsS amcA***	**Rm2011 *mVenus-rgsS$_{1-863}$***	**Rm2011 *mVenus-rgsS$_{1-863}$-mCherry***
**Rm2011 *mVenus-rgsS***		1.50E-03/**	3.09E-03/**	1.41E-03/**	4.87E-04/***
**Rm2011 *mVenus-rgsS amiC***			2.80E-01/ns	1.08E-01/ns	7.19E-02/ns
**Rm2011 *mVenus-rgsS amcA***				4.15E-02/*	5.41E-01/ns
**Rm2011 *mVenus- rgsS$_{1-863}$***					1.67E-02/*

Significance of differences in signal localization at the old cell pole in mother cells

	**P-value/significance level**				
	**Rm2011 *mVenus-rgsS***	**Rm2011 *mVenus-rgsS amiC***	**Rm2011 *mVenus-rgsS amcA***	**Rm2011 *mVenus-rgsS$_{1-863}$***	**Rm2011 *mVenus-rgsS$_{1-863}$-mCherry***
**Rm2011 *mVenus-rgsS***		1.91E-03/**	1.91E-02/*	1.34E-02/*	1.57E-02/*
**Rm2011 *mVenus-rgsS amiC***			3.55E-01/ns	1.16E-01/ns	5.10E-02/ns
**Rm2011 *mVenus-rgsS amcA***				6.25E-01/ns	3.27E-01/ns
**Rm2011 *mVenus- rgsS$_{1-863}$***					5.25E-01/ns

Supplementary Table 6. Polar localization of mVenus-RgsS, Pal-mCherry and HADA-stained zones in cells exponentially growing in TY medium. Only cells with polar mVenus-RgsS foci were considered. Percentage values from three independent biological replicates (500 to 546 cells each) were used to determine the mean percentage values. SD, standard deviation of the mean. The significance of the differences between the two strains was determined in a two-tailed t-test with unequal variance. P≥0.05, non-significant (ns); P<0.05, *; P<0.01, **; P<0.001, ***. Categories 4 and 7 summarize cells with either unipolar or bipolar allocation of Pal-mCherry fluorescence foci, respectively.

**Strain**		**Rm2011 *mVenus-rgsS***	**Rm2011 *mVenus-rgsS amiC***	**p-value/significance level of difference between the two strains**
**mVenus-RgsS, HADA, Pal-mCherry at one cell pole, % (± SD)**	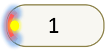 1	**91.29** (± 0.63)	**53.82** (± 1.77)	2.03E-04/***
**mVenus-RgsS, Pal the same pole, diffuse HADA signal, % (± SD)**	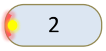 2	**8.38** (± 0.28)	**5.23** (± 0.55)	3.10E-03/***
**mVenus-RgsS and HADA at one cell pole, Pal-mCherry at both poles, % (± SD)**	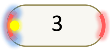 3	**0.26** (± 0.30)	**7.61** (± 1.04)	3.94E-03/**
**mVenus-RgsS and HADA at one cell pole, Pal-mCherry at the opposite, % (± SD)**	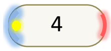 4	0.00 (± 0.00)	**21.93** (± 1.56)	1.67E-03/**
**mVenus-RgsS, Pal opposite poles, diffuse HADA signal, % (± SD)**	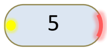 5	0.00 (± 0.00)	**5.52** (± 1.58)	2.62E-02/*
**mVenus-RgsS, HADA at both cell poles, % (± SD)**	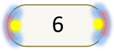 6	0.00 (± 0.00)	**2.50** (± 0.55)	1.57E-02/*
**mVenus-RgsS both poles, diffuse HADA signal, % (± SD)**	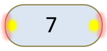 7	0.00 (± 0.00)	**3.32** (± 1.28)	4.61E-02/*
**n (cells)**		**1564**	**1527**	

Supplementary Table 7. Relocation of the second ParB-cerulean fluorescence focus from the old cell pole, containing mVenus-RgsS focus, to the new cell pole devoid of mVenus-RgsS focus. Relocation of ParB-cerulean focus was considered incomplete if it did not reach the opposite cell pole before the cell constriction became apparent. Percentage values from three to four independent time-lapse experiments were used to determine the mean percentage values. 26 to 72 former daughter cells with mVenus-RgsS focus at the old cell pole were analyzed in each time-lapse experiment. SD, standard deviation of the mean. The significance of the differences between the strains was determined in a two-tailed t-test with unequal variance. P≥0.05, non-significant (ns); P<0.05, *; P<0.01, **; P<0.001, ***.

**Strain**	**Rm2011 *mVenus-rgsS amiC***	**Rm2011 *mVenus-rgsS amcA***	**Rm2011 *mVenus-rgsS*$_{1-863}$*-mCherry***
**Cells with incomplete ParB-cerulean relocation, % (± SD)**	38.56 (± 0.29)	39.18 (± 4.52)	42.03 (± 8.82)
**Cells with complete ParB-cerulean relocation, % (± SD)**	61.44 (± 0.29)	60.82 (± 4.52)	57.97 (± 8.82)
**n (cells)**	158	137	128

Significance of differences in ParB-cerulean relocation

	**P-value/significance level**		
	**Rm2011 *mVenus-rgsS amiC***	**Rm2011 *mVenus-rgsS amcA***	**Rm2011 *mVenus- rgsS*$_{1-863}$*-mCherry***
**Rm2011 *mVenus-rgsS amiC***		8.02E-01/ns	4.89E-01/ns
**Rm2011 *mVenus-rgsS amcA***			5.39E-01/ns

Supplementary Table 8. Positioning of mVenus-RgsS fluorescence focus in cells that inherited ParB-cerulean at the new cell pole. Percentage values from three to four independent time-lapse experiments were used to determine the mean percentage values. 9 to 30 cells that inherited ParB-cerulean focus at the new cell pole were analyzed in each time-lapse experiment. SD, standard deviation of the mean. The significance of the differences between the strains was determined in a two-tailed t-test with unequal variance. P≥0.05, non-significant (ns); P<0.05, *; P<0.01, **; P<0.001, ***.

**Strain**	**Rm2011 *mVenus-rgsS amiC***	***mVenus-rgsS amcA***	**Rm2011 *mVenus-rgsS*$_{1-863}$*-mCherry***
**mVenus-RgsS stayed at the new cell pole, % (± SD)**	39.71 (± 12.89)	46.84 (± 8.12)	53.73 (± 11.90)
**mVenus-RgsS relocated to the old cell pole, % (± SD)**	60.29 (± 12.89)	53.16 (± 8.12))	46.27 (± 11.90)
**n (cells)**	68	58	85

Significance of differences in localization of mVenus-RgsS fluorescence focus

	**P-value/significance level**		
	**Rm2011 *mVenus-rgsS amiC***	**Rm2011 *mVenus-rgsS amcA***	**Rm2011 *mVenus-rgsS*$_{1-863}$*-mCherry***
**Rm2011 *mVenus-rgsS amiC***		4.59E-01/ns	2.12E-01/ns
**Rm2011 *mVenus-rgsS amcA***			3.81E-01/ns

Supplementary Table 9. Numerical data from single-molecule tracking of mVenus-RgsS and mVenus-RgsS-mCherry molecules as determined using SMTracker. lv, Levene test; tt, Student's t-test ch, Pearson's Chi-square significance test. *** indicate p<0.01, high level of significance. P≥0.01, not significant (ns). Ꞇ values were obtained in the analysis presented on the Supplementary Figure 23.

**Strain**	**Rm2011 *mVenus-rgsS***	**Rm2011 *mVenus-rgsS amiC***	**Rm2011 *mVenus-rgsS*$_{1-863}$**	**Rm2011 *mVenus-rgsS*$_{1-863}$*-mCherry***
**# movies**	47	30	35	25
**# cells**	383	350	322	393
**av. cell length [µm]**	2.72	2.35	2.25	1.6
**# tracks**	7556	5439	2296	5469
**av. lifetime [frames / [s]**	10 / 0.21	9.5 / 0.19	8 / 0.16	9.4 / 0.19
**#tracks/cell**	19.7285	15.54	7.1304	13.916
**#steps/cell**	200.4085	130.6066	46.6121	114.7839
**dwell radius cut [nm]**	120	120	120	120
**min dwell time [s]**	0.1836	0.1836	0.1836	0.1836
**Steps distribution free vs confinement [%]**	45 / 55	54 / 46	71 / 29	53 / 47
**confined tracks [%]**	28.1	21.7	13.2	23.2000
**mobile tracks [%]**	71.9	78.3	86.8	76.8
**free [%]**	68.3	73.2	83.8	72.9
**mixed behavior [%]**	3.6	5.2	2.9	3.9
**average residence time ± sd [s]**	0.344 ± 0.005	0.304 ± 0.004	0.270 ± 0.006	0.307 ± 0.005
**Ꞇ (1-comp.) ± sd [s]**	0.31 ± 0.0041	0.28 ± 0.0023	0.26 ± 0.0014	0.28 ± 0.003
**Ꞇ1 (2-comp.) ± sd [s]**	0.26 ± 0.0016	0.24 ± 0.0026	0.25 ± 0.0061	0.24 ± 0.002
**Ꞇ1 ± sd [%]**	72.6 ± 1.24	64.4 ± 3.22	91.2 ± 11	76.2 ± 2.03
**Ꞇ2 (2-comp.) ± sd [s]**	0.53 ± 0.01	0.38 ± 0.0093	0.38 ± 0.12	0.46 ± 0.015
**Ꞇ2 ± sd [%]**	27.4 ± 1.24	35.6 ± 3.22	8.83 ± 11	23.8 ± 2.03
**Diffusion constant**				
Static D ± sd [$\mu m^2 s^{-1}$]	0.022 ± 3.7e-05	0.022 ± 3.7e-05	0.022 ± 3.7e-05	0.022 ± 3.7e-05
Mobile D ± sd [$\mu m^2 s^{-1}$]	0.27 ± 0.0013	0.27 ± 0.0013	0.27 ± 0.0013	0.27 ± 0.0013
Static Fraction ± sd [%]	80 ± 0.08	67 ± 0.09	43 ± 0.13	72 ± 0.087
Mobile Fraction ± sd [%]	20 ± 0.08	33 ± 0.09	57 ± 0.13	28 ± 0.087
K-S GoF test	Rejected	Rejected	Rejected	Rejected
P-Value	2.70E-12	9.30E-10	7.30E-14	1.50E-05
R-Squared	1	1	0.999	1

Significance of differences in average residence times

**Significance / p-value**	**Rm2011 *mVenus-rgsS***	**Rm2011 *mVenus-rgsS amiC***	**Rm2011 *mVenus- rgsS*$_{1-863}$**	**Rm2011 *mVenus- rgsS*$_{1-863}$*-mCherry***
**Rm2011 *mVenus-rgsS***	-	(lv) *** / 4.7697e-13	(lv) *** / 4.5147e-14	(lv) *** / 3.2786e-08
**Rm2011 *mVenus-rgsS amiC***	-	-	(lv) *** / 8.7203e-07	(tt) ns / 0.68728
**Rm2011 *mVenus- rgsS*$_{1-863}$**	-	-	-	(lv) *** /

Supplementary Table 9. Continued.

Significance of differences in percentage of confined tracks				
**Significance / p-value**	**Rm2011 *mVenus-rgsS***	**Rm2011 *mVenus-rgsS amiC***	**Rm2011 *mVenus- rgsS*$_{1-863}$**	**Rm2011 *mVenus- rgsS*$_{1-863}$*-mCherry***
**Rm2011 *mVenus-rgsS***	-	(ch) *** /3.05e-10	(ch) *** /0	(ch) *** /7.73e-12
**Rm2011 *mVenus-rgsS amiC***	-	-	(ch) *** /0	(ch) ns /9.13e-1
**Rm2011 *mVenus- rgsS*$_{1-863}$**	-	-	-	(ch) *** /0

Significance of differences in percentage of free tracks				
**Significance / p-value**	**Rm2011 *mVenus-rgsS***	**Rm2011 *mVenus-rgsS amiC***	**Rm2011 *mVenus- rgsS*$_{1-863}$**	**Rm2011 *mVenus- rgsS*$_{1-863}$*-mCherry***
**Rm2011 *mVenus-rgsS***	-	(ch) *** /0	(ch) *** /0	(ch) *** /0
**Rm2011 *mVenus-rgsS amiC***	-	-	(ch) *** /0	(ch) ns /2.26e-1
**Rm2011 *mVenus- rgsS*$_{1-863}$**	-	-	-	(ch) *** /0

Significance of differences in diffusion constant				
**Significance / p-value**	**Rm2011 *mVenus-rgsS***	**Rm2011 *mVenus-rgsS amiC***	**Rm2011 *mVenus- rgsS*$_{1-863}$**	**Rm2011 *mVenus- rgsS*$_{1-863}$*-mCherry***
**Rm2011 *mVenus-rgsS***	-	(lv) *** / 0	(lv) *** / 0	(lv) *** / 2.31e-262
**Rm2011 *mVenus-rgsS amiC***	-	-	(lv) *** / 0	(lv) *** / 2.04e-31
**Rm2011 *mVenus- rgsS*$_{1-863}$**	-	-	-	(lv) *** / 0

Supplementary Table 10. Strains and plasmids used in this study.

Strain or plasmid	Properties	Reference
S. meliloti		
Rm2011	Wild type, Str^r	9
Rm2011 *mVenus-rgsS*	Rm2011 expressing *mVenus*-tagged *rgsS*, markerless insertion	10
Rm2011 *mVenus-rgsS amiC*	Rm2011 *mVenus-rgsS*, markerless *amiC* deletion	This work
Rm2011 *mVenus-rgsS amiD*	Rm2011 *mVenus-rgsS amiD*::pK18mob2-amiDplint, Km^r	This work
Rm2011 *mVenus-rgsS amcA*	Rm2011 *mVenus-rgsS*, markerless *smc03782* deletion	This work
Rm2011 *mVenus-rgsS amiC amiD*	Rm2011 *mVenus-rgsS amiC amiD*::pK18mob2-amiDplint, Km^r	This work
Rm2011 *mVenus-rgsS*$_{1-863}$	Rm2011 *mVenus-rgsS*::pK18mob2-rgsS$_{1-863}$, Km^r	This work
Rm2011 *mVenus- rgsS*$_{1-863}$*-mCherry*	Rm2011 *mVenus-rgsS*::pK18mob2-mCherry-rgsSΔSPOR, Km^r	This work
Rm2011 *3×flag-mucR*	Rm2011 expressing *3×flag*-tagged *mucR*, markerless insertion	11
Rm2011 *3×flag-mucR*	Rm2011 *3×flag-mucR amiC,* markerless deletion	This work
Rm2011 *3×flag-mucR*	Rm2011 *3×flag-mucR amcA,* markerless deletion	This work
Rm2011 *3×flag-mucR rgsS-3xflag*	Rm2011 *3×flag-mucR rgsS*::pG18mob-rgsS-CF, Gm^r	This work
Rm2011 *3×flag-mucR rgsS-3xflag amiC*	Rm2011 *3×flag-mucR amiC rgsS*::pG18mob-rgsS-CF, Gm^r	This work
Rm2011 *3×flag-mucR rgsS-3xflag amcA*	Rm2011 *3×flag-mucR amcA rgsS*::pG18mob-rgsS-CF*,* Gm^r	This work
Rm2011 *3×flag-mucR rgsS*$_{1-863}$*-3xflag*	Rm2011 *3×flag-mucR rgsS*::pG18mob-rgsS$_{1-863}$-CF, Gm^r	This work
Rm2011 *3×flag-mucR mVenus- rgsS*$_{1-863}$*-mCherry-3xflag*	Rm2011 *3×flag-mucR rgsS*::pG8mob-rgsS$_{1-863}$-mCherry-CF, Gm^r	This work
Rm2011 *mVenus-rgsS parB-cerulean*	Rm2011 *mVenus-rgsS* expressing *cerulean*-tagged *paB*, markerless insertion	This work
Rm2011 *mVenus-rgsS parB-cerulean amiC*	Rm2011 *mVenus-rgsS amiC* expressing cerulean-tagged paB, markerless insertion	This work
Rm2011 *mVenus-rgsS parB-cerulean amcA*	Rm2011 *mVenus-rgsS amcA* expressing cerulean-tagged paB, markerless insertion	This work
Rm2011 parB-cerulean *mVenus-rgsS rgsS*$_{1-863}$*-mCherry*	Rm2011 *parB-cerulean mVenus-rgsS*::pK18mob2-mCherry-rgsSDSPOR, Km^r	This work
Rm2011 *mVenus-rgsS rgsA-mCherry*	Rm2011 *mVenus-rgsS rgsA*::pK18mob2-rgsA-mCherry, Km^r	This work
Rm2011 *mVenus-rgsS rgsB-mCherry*	Rm2011 *mVenus-rgsS rgsB*::pK18mob2-rgsB-mCherry, Km^r	This work
Rm2011 *mVenus-rgsS rgsD-mCherry*	Rm2011 *mVenus-rgsS rgsD*::pK18mob2-rgsD-mCherry, Km^r	This work
Rm2011 *mVenus-rgsS rgsE-mCherry*	Rm2011 *mVenus-rgsS rgsE*::pK18mob2-rgsE-mCherry, Km^r	This work
Rm2011 *mVenus-rgsS rgsP-mCherry*	Rm2011 *mVenus-rgsS rgsP*::pK18mob2-rgsP-mCherry, Km^r	This work
Rm2011 *mVenus-rgsS tolQ-mCherry*	Rm2011 *mVenus-rgsS tolQ*::pK18mob2-tolQprCDS-mCherry, Km^r	This work
Rm2011 *mVenus-rgsS pal-mCherry*	Rm2011 *mVenus-rgsS pal*::pK18mob2-pal-mCherry, Km^r	This work
Rm2011 *mVenus-rgsS amiC rgsA-mCherry*	Rm2011 *mVenus-rgsS amiC rgsA*::pK18mob2-rgsA-mCherry, Km^r	This work
Rm2011 *mVenus-rgsS amiC rgsB-mCherry*	Rm2011 *mVenus-rgsS amiC rgsB*::pK18mob2-rgsB-mCherry, Km^r	This work
Rm2011 *mVenus-rgsS amiC rgsD-mCherry*	Rm2011 *mVenus-rgsS amiC rgsD*::pK18mob2-rgsD-mCherry, Km^r	This work

Supplementary Table 10. Continued.

Strain or plasmid	Properties	Reference
Rm2011 *mVenus-rgsS amiC rgsE-mCherry*	Rm2011 *mVenus-rgsS amiC rgsE*::pK18mob2-rgsE-mCherry, Km^r	This work
Rm2011 *mVenus-rgsS amiC rgsP-mCherry*	Rm2011 *mVenus-rgsS amiC rgsP*::pK18mob2-rgsP-mCherry, Km^r	This work
Rm2011 *mVenus-rgsS amiC tolQ-mCherry*	Rm2011 *mVenus-rgsS amiC tolQ*::pK18mob2-tolQprCDS-mCherry, Km^r	This work
Rm2011 *mVenus-rgsS amiC pal-mCherry*	Rm2011 *mVenus-rgsS amiC rgsA*::pK18mob2-pal-mCherry, Km^r	This work
E. coli		
DH5α	F– *endA1 glnV44 thi-1 recA1 relA1 gyrA96 deoR nupG purB20* φ80d*lacZ*ΔM15 Δ(*lacZYA-argF*)U169, hsdR17(*rK*–*mK*+), λ–	12
S17-1	*E. coli* 294 Thi RP4-2-Tc::Mu-Km::Tn7 integrated into the chromosome	13
MG1655	K12, F- lambda- *ilvG- rfb*-50 *rph*-1	14
JW2428	*amiA* deletion strain, Km^r	15
JW4127	*amiB* deletion strain, Km^r	15
JW5449	*amiC* deletion strain, Km^r	15
MG1655 *amiABC*	*amiA, amiB* and *amiC* deletion strain, carrying pSRKGm-SP-mCherry-SPOR, Km^r, Gm^r	This work
**Plasmids**		
pABC-Psyn	Plasmid vector, single-copy in *S. meliloti*, $Spec^r$	9
pK18mob2	Suicide vector, *lacZ*, *mob*, Km^r	16
pK18mobsacB	Suicide vector, *lacZ*, *mob*, *sacB,* Km^r	16
pSRKGm	pBBR1MCS-5-derived broad-host-range expression vector containing *lac* promoter and *lacI*q, *lacZ*α+, Gmr	17
pWBT-NF	pSRKGm with T5 promoter and 3xFLAG tag coding sequence, Gm^r	J. Hawkins
pK18mob2-mCherry	pK18mob2 carrying mCherry sequence, Km^r	This work
pG18mob-3×FLAG	pG18mob carrying 3×flag including a stop codon cloned into the XbaI and HindIII restriction sites, Gm^r	11
pSRKGm-SP-mCherry-SPOR	pSRKGm carrying coding sequence consisting of $RgsB_{1\text{-}25}$, mCherry lacking start and stop codon and $RgsS_{831\text{-}945}$ under lac promoter, Gm^r	This work
pSRKGm-SP-mCherry	pSRKGm carrying coding sequence consisting of $RgsB_{1\text{-}25}$, mCherry lacking start codon under lac promoter, Gm^r	This work
pWBT-NF-RgsS	pWBT-NF carrying RgsS coding sequence, Gm^r	This work
pWBT-NF-$RgsS_{1\text{-}863}$	pWBT-NF carrying RgsS coding sequence, amino acids 1-863, Gm^r	This work
pWBT-NF-$RgsS_{C931A}$	pWBT-NF carrying RgsS coding sequence, point C931A mutation, Gm^r	This work
pWBT-NF-$RgsS_{C941A}$	pWBT-NF carrying RgsS coding sequence, point C941A mutation, Gm^r	This work
pABC-amiC	pABC-Psyn carrying *amiC* promoter and coding sequence, $Spec^r$	This work
pABC-$amiC_{H206A}$	pABC-Psyn carrying *amiC* promoter and coding sequence, point mutation H206A, $Spec^r$	This work
pABC-$amiC_{H276A}$	pABC-Psyn carrying *amiC* promoter and coding sequence, point mutation H276A, $Spec^r$	This work
pABC-amcA	pABC-Psyn carrying *amcA* promoter and coding sequence, $Spec^r$	This work
pABC-amiC-CF	pABC-Psyn carrying *amiC* promoter and coding sequence, C-termially fused to 3xFLAG tag, $Spec^r$	This work
pABC-$amiC_{H206A}$-CF	pABC-Psyn carrying *amiC* promoter and coding sequence, point mutation H206A, C-termially fused to 3xFLAG tag,$Spec^r$	This work
pK18m2-$rgsS_{1\text{-}863}$	pK18mob2 carrying 3' portion of *rgsS* truncated at codon 863, Km^r	This work

Supplementary Table 10. Continued.

Strain or plasmid	Properties	Reference
pK18m2-rgsS$_{1-863}$-mCherry	pK18mob2-mCherry carrying 3' portion of *rgsS* truncated at codon 863 without stop codon, Km^r	This work
pK18mob2-amiDplInt	pK18mob2 carrying internal fragment of *amiD* coding sequence, Km^r	This work
pK18mobsacB-amiCdel	pK18mobsacB carrying *amiC* flanking regions, Km^r	This work
pK18mobsacB-amcAdel	pK18mobsacB carrying *amcA* flanking regions, Km^r	This work
pK18mob2-rgsA-mCherry	pK18mob2-mCherry carrying 3' portion of *rgsA*, Km^r	10
pK18mob2-rgsB-mCherry	pK18mob2-mCherry carrying 3' portion of *rgsB*, Km^r	10
pK18mob2-rgsD-mCherry	pK18mob2-mCherry carrying 3' portion of *rgsD*, Km^r	10
pK18mob2-rgsE-mCherry	pK18mob2-mCherry carrying 3' portion of *rgsE*, Km^r	10
pK18mob2-tolQ-mCherry	pK18mob2-mCherry carrying promoter region and coding sequence of *tolQ*, Km^r	10
pK18mob2-pal-mCherry	pK18mob2-mCherry carrying 3' portion of *pal*, Km^r	10
pMW198	pK18mobsacB carrying 3' portion of *parB* excluding the stop codon, *cerulean* and *parB* downstream region, Km^r	M. Wagner
pG18mob-rgsS-3×flag	pG18mob-3×flag carrying 3' portion of *rgsS*, Gm^r	This work
pG18mob- rgsS$_{1-863}$-3×flag	pG18mob-3×flag carrying 3' portion of *rgsS* up to amino acid 863, followed by a stop codon, Gm^r	This work
pG18mob- rgsS$_{1-863}$-mCherry 3×flag	pG18mob-3×flag carrying 3' portion of *rgsS* up to amino acid 863, followed by mCherry coding sequence, Gm^r	This work
pCP20	*FLP*$^+$, λ *c*I857$^+$, λ p_R Rep^{ts}, Ap^r, Cm^r	18

Supplementary Table 11. Oligonucleotides used in this study.

Primer	Sequence	Cloning destination
mCh_SP_04006-Nde-f	CAATCATATGACGTTTGGATCCATCCTCCTGTTTTCCTTGG CAATTTCCGCTTCATTTCTCTCTGCCGGCGATGCCCGCGCC AGCAAGGGCGAGGAGGATAAC	mCherry-SPOR expression plasmid
EGFP/mCh-Xba-r-nostop	GTACTCTAGACTTGTACAGCTCGTCCATG	
Smc02072+2491-Xba-f	GGATCTAGACCGGCCGCTCAGCCGGAGG	
Smc02072-stop-Hind-r	CGACAAGCTTGTTTATTTCGTTACCAGGCAGC	
SMc02072+2589-X-st-r	GCGTCTAGATTAAACCGAAGCCGTCGCCAGCG	SPOR domain truncation plasmids
Smc02072+1864-Sal-f	CTAGGTCGACGGCAGCAACGCGGTGCTTTCC	
Smc02072+2589-Xba-r	GCGTCTAGAAACCGAAGCCGTCGCCAGCG	
amiCdel1-Sal-f	ATATGTCGACGAGTGAGACGCATCGATAAGC	Markerless deletion constructs
amiCdel1-Xba-r	ATATCTAGAGTCGCCGAACGCTTCACCTG	
amiCdel2-Xba-f	ATATCTAGAGCGCAGGGCGTTCCGGGATCC	
amiCdel2-Eco-r	GAAGGAATTCATGCCCGGATGCTGGTAGAAG	
Smc03782del1-Eco-f	ATATGAATTCGTTGCCATCGCCGATAAGGCG	
Smc03782del1-Xba-r2	ATATCTAGAAAAAGTTAGGAGACCCTAATC	
Smc03782del2-Xba-f2	ATATCTAGAGCAGAGGTTGGAAGGGCG	
Smc03782del2-Hind-r	GCTCAAGCTTACTTCCGCCTGGATCTTCTCG	
amiC-368-Not-f	GACGGCGGATGAATTCCTGCG	pABC-Psyn complementation constructs
amiC-stop-Kpn-r	ATATGGTACCTCAACCGCCGTTTGCAACCG	
03782-401-Pac-f	ACTGTTAATTAAAAAGCGCTCGCCGACGGCA	
03782-stop-Xba-r	ATATCTAGACTACGTATCATTTCGCGCCC	
amiC_H206A-f	GTTGCGATAGATGCCGGGGCCGGCGGAATCGATACCGG TGCG	Site-directed mutagenesis *amiC*
amiC_H206A-r	GGCCCCGGCATCTATCGCAAC	
amiC_H276A-f	AACCTGTTCATTTCGGTTGCCGCCGACACGTTGCGGCAAA AG	
amiC_H276A-r	GGCAACCGAAATGAACAGGTT	
EGFP/mCh-f-Xba	GAGTCTAGAATGGTGAGCAAGGGCGAGGAG	pK18mob2-mCherry
EGFP/mCh-endEco	GTACGAATTCTTACTTGTACAGCTCGTCCATG	
amiD+30-Hind-f	ATATAAGCTTCGCTCATCTCGTGCCCTCGCC	*amiD* mutation plasmid
amiD+350-Xba-r	ATATCTAGATCTGTGCCTCAGGGAAATCC	
Smc02072+1-Xba-f	CACTCTAGAATGGCAGACAAACAATTCGCAC	Ectopic N-terminally fused 3xFLAG tag consructs
Smc02072+2440-Hind-r	cgagaagcttaCTTCGGACGCTGCAGCGAGC	
Smc02072_C941A-Hind-r	ATATAAGCTTATTTCGTTACCAGGGCGCTTCCGCCGGCGC TTTTGTAG	
Smc02072_C931A-Hind-r	ATATAAGCTTATTTCGTTACCAGGCAGCTTCCGCCGGCGC TTTTGTAGCGAGAGGCAAGCGAATTGGCCTCCTCGC	
Smc02072-stop-Hind-r	CGACAAGCTTGTTTATTTCGTTACCAGGCAGC	

Supplementary References

1. Käll, L., Krogh, A., and Sonnhammer, E. L. L. A combined transmembrane topology and signal peptide prediction method. *J. Mol. Biol.* **338,** 1027-1036 (2004).
2. Källberg, M. et al. RaptorX server: a resource for template-based protein structure modeling. *Methods Mol. Biol*. **1137**, 17-27 (2014).
3. Waterhouse, A. et al. SWISS-MODEL: homology modelling of protein structures and complexes. *Nucleic Acids Res.* **46**, W296-W303 (2018).
4. Mishima, M. et al. Solution structure of the peptidoglycan binding domain of *Bacillus subtilis* cell wall lytic enzyme CwlC: characterization of the sporulation-related repeats by NMR. *Biochemistry*. **44**, 10153-10163 (2005).
5. Yahashiri, A. et al. The SPOR Domain, a widely conserved peptidoglycan binding domain that targets proteins to the site of cell division. *J. Bacteriol*. **199**, e00118-17 (2017).
6. Alcorlo, M. et al. Structural basis of denuded glycan recognition by SPOR domains in bacterial cell division. *Nat. Commun.* **10**, 5567 (2019).
7. Combet, C. et al. Network Protein Sequence Analysis. *Trends Biochem Sci*. **25**, 147-150 (2000).
8. Peters, N. T. et al. Structure-function analysis of the LytM domain of EnvC, an activator of cell wall remodelling at the *Escherichia coli* division site. *Mol. Microbiol*. 89, 690-701 (2013).
9. Casse, F. et al. Identification and characterization of large plasmids in *Rhizobium meliloti* using agarose gel electrophoresis. *Microbiology* **113**, 229-242 (1979).
10. Krol, E. et al. Tol-Pal system and Rgs proteins interact to promote unipolar growth and cell division in *Sinorhizobium meliloti*. *mBio* **11**, e00306-00320 (2020).
11. Schäper, S. et al. Seven-transmembrane receptor protein RgsP and cell wall-binding protein RgsM promote unipolar growth in Rhizobiales. PLoS Genet **14**, e1007594 (2018).
12. Grant, S. G et al. Differential plasmid rescue from transgenic mouse DNAs into *Escherichia coli* methylation-restriction mutants. *Proc. Natl. Acad. Sci. USA* **87**, 4645-4649 (1990).
13. Simon, R. et al. A broad host range mobilization system for in vivo genetic engineering: transposon mutagenesis in gram-negative bacteria. *Nature Biotechnology* **1**, 784-791 (1983).
14. Blattner, F. R. et al. The complete genome sequence of *Escherichia coli* K-12. *Science* **277**, 1453-1462 (1997).
15. Baba T. et al. Construction of *Escherichia coli* K-12 in-frame, single-gene knockout mutants: the Keio collection. *Mol. Syst. Biol.* **2**, 2006.0008 (2006).
16. Schäfer, A et al. Small mobilizable multi-purpose cloning vectors derived from the *Escherichia coli* plasmids pK18 and pK19: selection of defined deletions in the chromosome of *Corynebacterium glutamicum*. *Gene* **145**, 69-73 (1994).
17. Khan, S. R., Gaines, J., Roop, R. M. & Farrand, S. K. Broad-host-range expression vectors with tightly regulated promoters and their use to examine the influence of TraR and TraM expression on Ti plasmid quorum sensing. *Appl Environ Microbiol* **74**, 5053-5062 (2008).
18. Cherepanov, P.P. & Wackernagel, W. Gene disruption in *Escherichia coli*: Tc^R and Km^R cassettes with the option of Flp-catalyzed excision of the antibiotic-resistance determinant. *Gene* **158**, 9-14 (1995).
