# Supplementary material for: Stable inheritance of Sinorhizobium meliloti cell growth polarity requires an FtsN-like protein and an amidase
Source: Nat Commun. 2021 Jan 22;12:545. doi: 10.1038/s41467-020-20739-3 (PMC7822825; doi:10.1038/s41467-020-20739-3)
Supplement: Supplementary file 2 — Description of Additional Supplementary Files [file 41467_2020_20739_MOESM2_ESM.pdf]

Description of Additional Supplementary Files

File Name: Supplementary Movie 1
Description: **Representative raw data movie from single molecule tracking microscopy of Rm2011 *mVenus-rgsS*.** The movie (3000 frames, 20 msec interval) shows the bleaching of the mVenus-RgsS molecules and in later frames the single molecule movement of mVenus-RgsS used to generate the trajectories for subsequent analysis.
