# Supplementary material for: Stable inheritance of Sinorhizobium meliloti cell growth polarity requires an FtsN-like protein and an amidase
Source: Nat Commun. 2021 Jan 22;12:545. doi: 10.1038/s41467-020-20739-3 (PMC7822825; doi:10.1038/s41467-020-20739-3)
Supplement: Supplementary file 4 — Reporting Summary [file 41467_2020_20739_MOESM4_ESM.pdf]

nature research

Corresponding author(s): *Double-blind peer review submissions: write DBPR and your manuscript number here instead of author names.*

Last updated by author(s): *YYYY-MM-DD*

# Reporting Summary

Nature Research wishes to improve the reproducibility of the work that we publish. This form provides structure for consistency and transparency in reporting. For further information on Nature Research policies, see our Editorial Policies and the Editorial Policy Checklist.

## Statistics

For all statistical analyses, confirm that the following items are present in the figure legend, table legend, main text, or Methods section.

| n/a | Confirmed | |
|---|---|---|
| ☐ | ☒ | The exact sample size (*n*) for each experimental group/condition, given as a discrete number and unit of measurement |
| ☐ | ☒ | A statement on whether measurements were taken from distinct samples or whether the same sample was measured repeatedly |
| ☐ | ☒ | The statistical test(s) used AND whether they are one- or two-sided<br>*Only common tests should be described solely by name; describe more complex techniques in the Methods section.* |
| ☒ | ☐ | A description of all covariates tested |
| ☒ | ☐ | A description of any assumptions or corrections, such as tests of normality and adjustment for multiple comparisons |
| ☐ | ☒ | A full description of the statistical parameters including central tendency (e.g. means) or other basic estimates (e.g. regression coefficient) AND variation (e.g. standard deviation) or associated estimates of uncertainty (e.g. confidence intervals) |
| ☒ | ☐ | For null hypothesis testing, the test statistic (e.g. *F*, *t*, *r*) with confidence intervals, effect sizes, degrees of freedom and *P* value noted<br>*Give P values as exact values whenever suitable.* |
| ☒ | ☐ | For Bayesian analysis, information on the choice of priors and Markov chain Monte Carlo settings |
| ☒ | ☐ | For hierarchical and complex designs, identification of the appropriate level for tests and full reporting of outcomes |
| ☒ | ☐ | Estimates of effect sizes (e.g. Cohen's *d*, Pearson's *r*), indicating how they were calculated |

*Our web collection on statistics for biologists contains articles on many of the points above.*

## Software and code

Policy information about availability of computer code

| | |
|---|---|
| Data collection | NIS Elements 4.13.4 (Nikon), Utrack 2.2.1 |
| Data analysis | NIS Elements 4.13.4 (Nikon), SMTracker, BLASTP online tool, Phobius online tool, Raptorx online tool, Coiled-coils online tool, SWISS-MODEL online tool |

For manuscripts utilizing custom algorithms or software that are central to the research but not yet described in published literature, software must be made available to editors and reviewers. We strongly encourage code deposition in a community repository (e.g. GitHub). See the Nature Research guidelines for submitting code & software for further information.

## Data

Policy information about availability of data

All manuscripts must include a data availability statement. This statement should provide the following information, where applicable:

- Accession codes, unique identifiers, or web links for publicly available datasets
- A list of figures that have associated raw data
- A description of any restrictions on data availability

Numerical data underlying Figs. 2A, 2C and 4B, Supplementary Figs.4B-D, 18 and 24, as well as Supplementary Tables 1-8 are provided as Source Data file, The authors declare that the main data supporting the findings of this study are available within the article and its Supplementary information files. All other data are available from the corresponding author upon request.

# Field-specific reporting

Please select the one below that is the best fit for your research. If you are not sure, read the appropriate sections before making your selection.

- [x] Life sciences
- [ ] Behavioural & social sciences
- [ ] Ecological, evolutionary & environmental sciences

For a reference copy of the document with all sections, see nature.com/documents/nr-reporting-summary-flat.pdf

# Life sciences study design

All studies must disclose on these points even when the disclosure is negative.

| | |
|---|---|
| Sample size | For single cell fluorescence microscopy analyses of exponentially growing cultures, two to four independent cultivations and microscopy analyses were performed. In case of numeric evaluation of fluorescence patterns in cells in exponentially growing cultures, the cell number per replicate was aimed at the minimum of 300 and in case of time-lapse analysis, at the minimum of 100 cells. In most cases, the actual numbers of analyzed cells substantially exceeded the minimum. For growth curve analysis and Western blot analysis, either three or four replicates were used. |
| Data exclusions | No data was excluded from the analysis |
| Replication | All attempts at replication were successful. The experiments were replicated at least once. |
| Randomization | Not relevant to this study, since no experimental groups were generated |
| Blinding | Not relevant to this study, since no experimental groups were generated |

# Reporting for specific materials, systems and methods

We require information from authors about some types of materials, experimental systems and methods used in many studies. Here, indicate whether each material, system or method listed is relevant to your study. If you are not sure if a list item applies to your research, read the appropriate section before selecting a response.

## Materials & experimental systems

| n/a | Involved in the study | |
|---|---|---|
| ☐ | ☒ | Antibodies |
| ☒ | ☐ | Eukaryotic cell lines |
| ☒ | ☐ | Palaeontology and archaeology |
| ☒ | ☐ | Animals and other organisms |
| ☒ | ☐ | Human research participants |
| ☒ | ☐ | Clinical data |
| ☒ | ☐ | Dual use research of concern |

## Methods

| n/a | Involved in the study | |
|---|---|---|
| ☒ | ☐ | ChIP-seq |
| ☒ | ☐ | Flow cytometry |
| ☒ | ☐ | MRI-based neuroimaging |

## Antibodies

| | |
|---|---|
| Antibodies used | Monoclonal ANTI-FLAG® M2-Peroxidase (HRP) antibody produced in mouse (Sigma-Aldrich) |
| Validation | Commercial antibody against the FLAG tag, species-independent. The Monoclonal ANTI-FLAG M2-Peroxidase is a mouse IgG antibody covalently conjugated to horseradish peroxidase (HRP). The antibody binds to FLAG fusion proteins and recognizes the FLAG epitope at FLAG peptides. ANTI-FLAG is a registered trademark of Sigma-Aldrich Co. LLC |
